# Assembly and Functionality of 2D Protein Arrays

**DOI:** 10.1002/advs.202416485

**Published:** 2025-03-16

**Authors:** Mingming Du, Fanmeng Zeng, YueFei Wang, Ying Li, Guangcun Chen, Jiang Jiang, Qiangbin Wang

**Affiliations:** ^1^ CAS Key Laboratory of Nano‐Bio Interface Division of Nanobiomedicine and *i*‐Lab Suzhou Institute of Nano‐Tech and Nano‐Bionics Chinese Academy of Sciences Suzhou 215123 China; ^2^ School of Physical Science and Technology ShanghaiTech University Shanghai 201210 China; ^3^ College of Materials Sciences and Opto‐Electronic Technology University of Chinese Academy of Sciences Beijing 100049 China

**Keywords:** 2D protein arrays, biomedical applications, functional biomaterials, nanoparticles, self‐assembly

## Abstract

Among the unique classes of 2D nanomaterials, 2D protein arrays garner increasing attention due to their remarkable structural stability, exceptional physiochemical properties, and tunable electronic and mechanical attributes. The interest in mimicking and surpassing the precise architecture and advanced functionality of natural protein systems drives the field of 2D protein assembly toward the development of sophisticated functional materials. Recent advancements deepen the understanding of the fundamental principles governing 2D protein self‐assembly, accelerating the creation of novel functional biomaterials. These developments encompass biological, chemical, and templated strategies, facilitating the self‐organization of proteins into highly ordered and intricate 2D patterns. Consequently, these 2D protein arrays create new opportunities for integrating diverse components, from small molecules to nanoparticles, thereby enhancing the performance and versatility of materials in various applications. This review comprehensively assesses the current state of 2D protein nanotechnology, highlighting the latest methodologies for directing protein assembly into precise 2D architectures. The transformative potential of 2D protein assemblies in designing next‐generation biomaterials, particularly in areas such as biomedicine, catalysis, photosystems, and membrane filtration is also emphasized.

## Graphical abstract

2D protein arrays‐based outstanding properties, including their remarkable structural stability, exceptional physiochemical properties, and tunable electronic and mechanical attributes, have become a key functional platform for developing advanced functional materials. This review comprehensively assesses the latest advancements in 2D protein self‐assembly, covering biological, chemical, and templated strategies, and explores their potential function and applications in biomedicine, catalysis, photosystems, and membrane filtration.

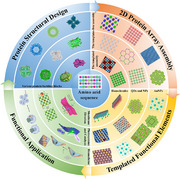

## 1 Introduction

Proteins, as highly adaptable biomolecules, exhibit a wide range of structural complexities, chemical diversities, and functional capabilities.^[^ ^1^, ^2^, ^3^, ^4^ ^]^ These characteristics enable them to form both static and dynamic structures, which play crucial roles in specialized cellular processes.^[^ ^5^, ^6^, ^7^, ^8^, ^9^, ^10^ ^]^ Drawing inspiration from natural biological systems, proteins have been recognized as optimal building blocks for the bottom–up construction of sophisticated multidimensional superstructures.^[^ ^11^, ^12^, ^13^, ^14^, ^15^, ^16^ ^]^ Notably, 2D protein arrays have emerged as a promising approach for developing advanced bionanomaterials with highly organized architectures.^[^ ^17^ ^]^ Engineered 2D protein arrays possess several advantageous properties, such as remarkable structural stability, exceptional physiochemical properties, tunable electronic and mechanical attributes, high‐density functional sites, and nanoscale tunability.^[^ ^18^ ^]^ These features enable the precise arrangement of target molecules or nanoparticles (NPs), facilitating the creation of periodic patterns with sub‐10‐nanometer precision and supporting the hierarchical assembly of intricate multicomponent structures, which exceed the capabilities of traditional self‐assembly methods.^[^ ^19^ ^]^ The high surface area‐to‐volume ratio of 2D protein crystals further enhances their potential for applications in the engineering of heterogeneous biocatalysts and as platforms for biomedicine.^[^ ^20^ ^]^ Additionally, these 2D arrays offer valuable systems for studying biological and enzymatic reactions.^[^ ^21^, ^22^, ^23^ ^]^

**Figure 1:**
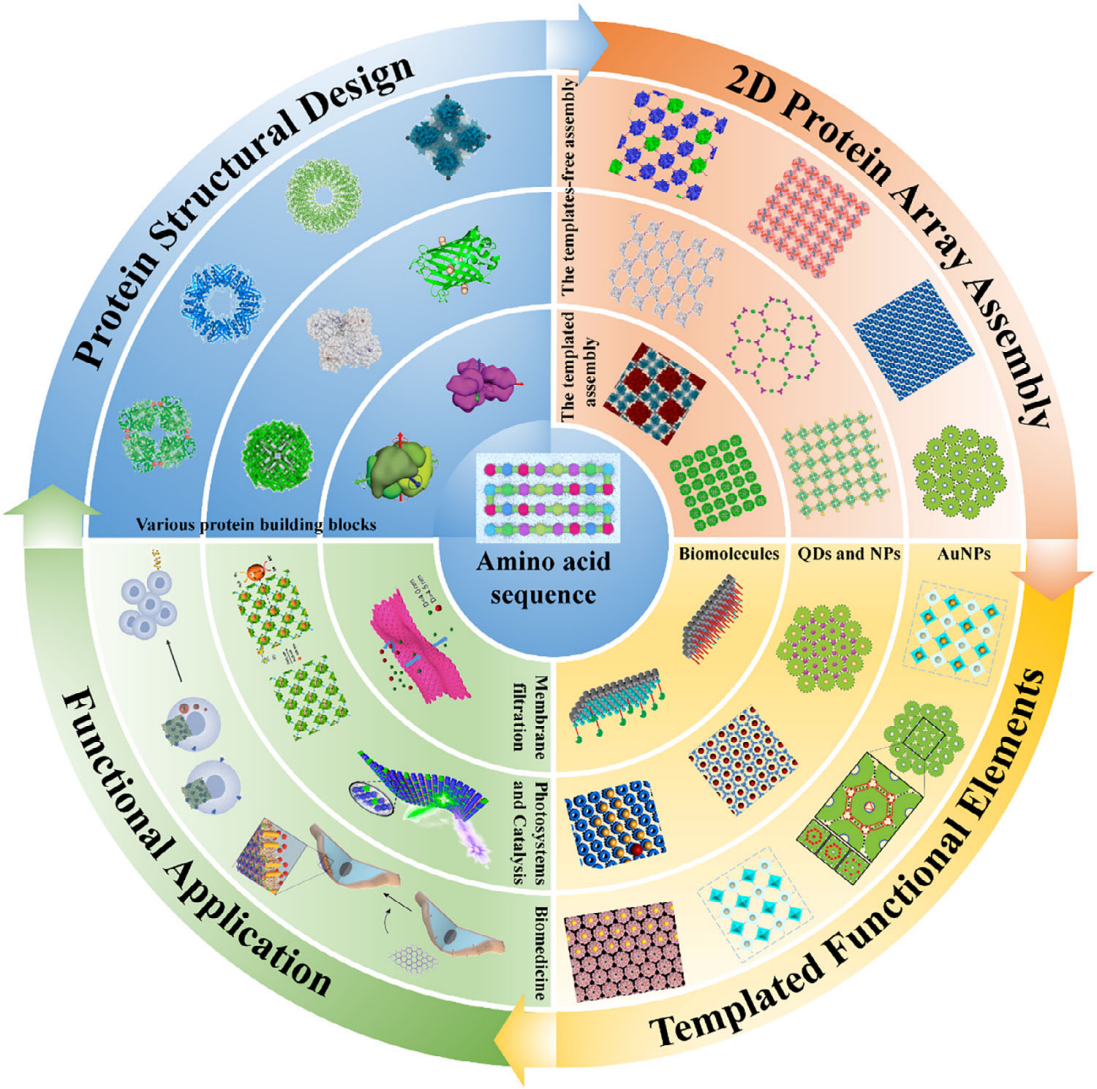
Schematic illustration of various strategies for 2D protein array assembly, incorporating templated functional elements and their diverse functional applications, including biomedicine, photosystem, catalysis, and membrane filtration.

Despite their significant potential, 2D protein arrays face several challenges, particularly in terms of structural complexity, conformational flexibility, low yield, poor uniformity, as well as limited scalability.^[^ ^24^, ^25^ ^]^ Thus, early research efforts primarily concentrated on biological assembly strategies, involving studying and engineering of protein interfaces and their structural characteristics, followed by exploring of their practical applications.^[^ ^26^, ^27^, ^28^, ^29^ ^]^ For example, natural 2D S‐layers were systematically exploited for many applications based on structural precision. Chemical cross‐linking was used to fabricate the active S‐layer ultrafiltration membranes for the filtration of a ferritin solution.^[^ ^30^ ^]^ Genetic fusion generated S‐layer‐streptavidin fusion proteins that served as templates for nanopatterned molecular arrays.^[^ ^31^ ^]^ However, the limited availability of structural data on S‐layer proteins hinders their manipulation into functional materials,^[^ ^32^ ^]^ necessitating innovative engineering strategy with greater control over the self‐assembly process. With advancements in supramolecular chemistry,^[^ ^33^, ^34^, ^35^ ^]^ chemical strategies have been introduced to fabricate protein assemblies with greater control over self‐assembly dynamics.^[^ ^36^ ^]^ To further advance the field, emerging engineering strategies of 2D protein arrays were developed (**Figure** **1**), displaying new functions and application potentials. For instance, genetic fusion offers a functional interface for macromolecule arrangement,^[^ ^37^, ^38^, ^39^ ^]^ computational design allows angstrom‐level precision for new structure determination,^[^ ^40^ ^]^ covalent bonds provide mechanical robustness for investigating the energy landscape of lattice dynamics,^[^ ^41^ ^]^ metal‐ion‐induced interactions regulate conformational states in response to environmental cues,^[^ ^42^, ^43^ ^]^ DNA origami templates assemble functional elements with precise spacing.^[^ ^44^, ^45^ ^]^ These advancements have broadened the potential applications of 2D protein arrays as functional platforms in catalysis, membrane filtration, and biomedicine.^[^ ^17^, ^46^ ^]^

This review aims to provide a comprehensive overview of the recent advancements and emerging trends in 2D protein nanotechnology, spanning design, assembly, function to applications. It will highlight reliable biological, chemical, and templated strategies to construct diverse 2D architectures. As protein assembly technologies continue to evolve, 2D protein arrays with well‐defined structures are increasingly being functionalized, opening up a multitude of applications in biomedicine, photosystems, catalysis, and membrane filtration. The review seeks to elucidate the exciting potential and future directions of this rapidly advancing field, underscoring both the opportunities and the challenges that lie ahead.

## 2 The Templates‐Free Assembly in Solution

Ordered 2D protein assemblies can form in solution in the absence of templates, by exploiting electrostatic interactions, *π–π* and hydrophobic interactions, covalent bonding, and metal coordination. Moreover, these strategies can integrate natural and synthetic elements, leveraging designed supramolecular interactions to create novel protein assemblies. By modifying the structural elements and functional groups within protein complexes, these approaches have substantially expanded the scope of protein assembly, opening new avenues for the design and fabrication of advanced biomaterial. In the following sections, research progresses are categorized according to the specific type of major interactions that drive 2D protein self‐assembly, as well as the design principle of the interacting units.

### 2.1 Electrostatic Interactions

Electrostatic interactions have become a fundamental force to consider in the field of protein assembly, primarily due to that protein–protein interactions (PPIs) can be regulated by their surface charges.^[^ ^47^, ^48^ ^]^ This strategy leverages the natural charge distribution on protein surfaces to enable the creation of stable protein assemblies with defined geometries and functions.^[^ ^49^, ^50^ ^]^ By adjusting parameters such as pH and ionic strength, researchers can fine‐tune the strength and directionality of electrostatic forces, thereby guiding the assembly process to produce assemblies with desired structural characteristics.^[^ ^51^ ^]^ The key advantages of using electrostatic interactions in protein assembly are their high specificity and reversibility. The dynamic nature of these interactions allows for the disassembly and reassembly of protein arrays in response to environmental changes, making this approach particularly suitable for applications that require adaptive or responsive biomaterials.^[^ ^52^ ^]^

**Figure 2:**
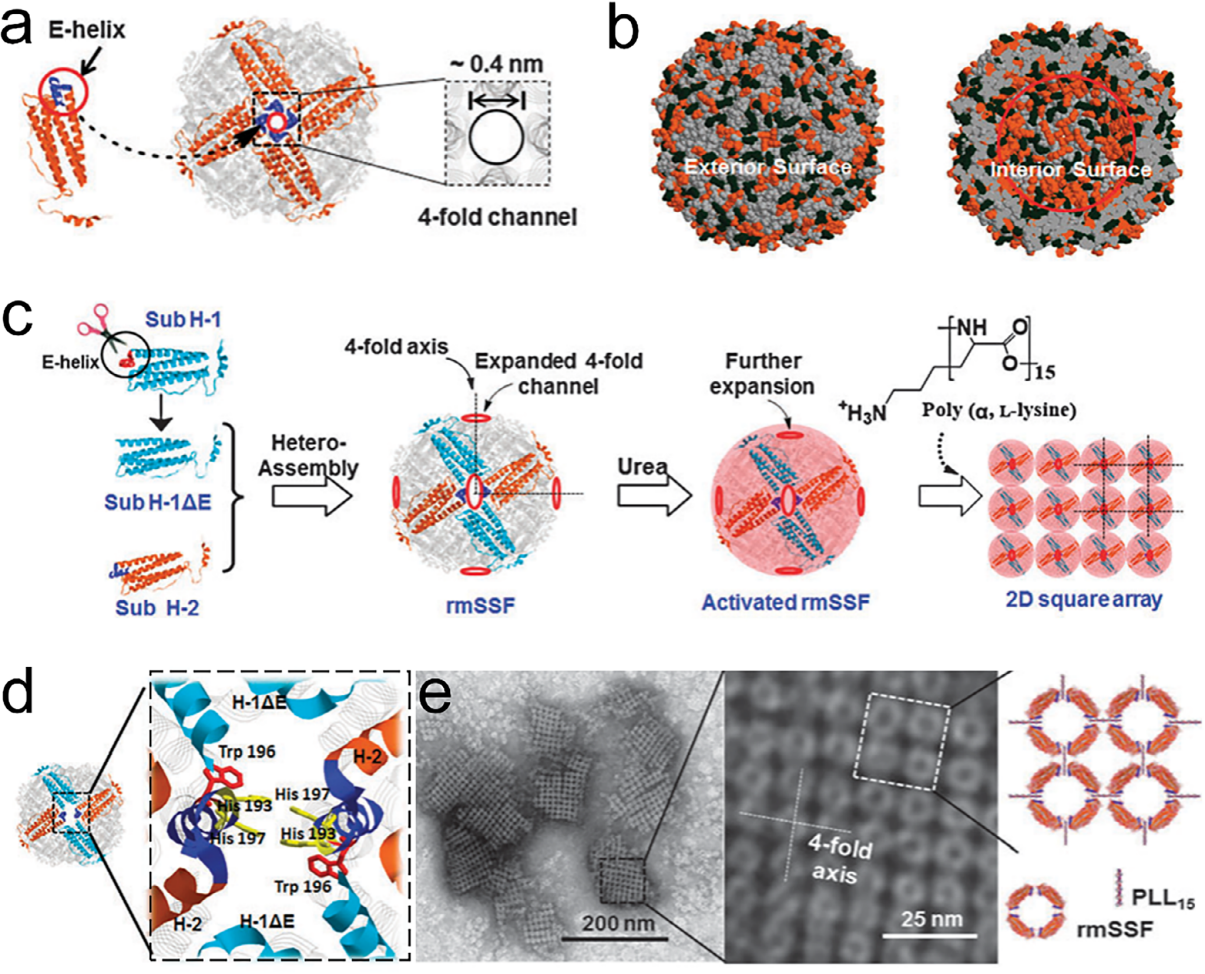
a) Schematic of the subunit and spherical shell of plant ferritin. b) Distribution of negative (orange) and positive (dark green) charges on the interior and exterior surfaces of plant ferritin at pH 7.0. c) Schematic representation of 2D soybean seed ferritin (rmSSF) array formation. d) Key amino acids around the fourfold channel of rmSSF. e) TEM images of rmSSF lattices induced by PLL_15_ (poly‐L‐lysine with a polymerization degree of 15) in the presence of urea (10 mm) at pH 7.0, with a schematic for the disposition of rmSSF and PLL_15_. Reproduced with permission.^[^ ^55^ ^]^ Copyright 2014, Royal Society of Chemistry.

Symmetrical proteins can be engineered to carry specific charges, which can either attract or repel other charged entities, facilitating the formation of ordered 2D arrays.^[^ ^53^ ^]^ For instance, given the high negative charges in the interior cavity of ferritin, Yang et al. developed a strategy to assemble ferritin cages into 2D arrays through electrostatic interactions.^[^ ^54^, ^55^ ^]^ They first deleted all E‐helices from the 12 H‐1 subunits (**Figure** **2**a,b) to expand the fourfold channels of the reconstructed soybean seed ferritin (rmSSF). The addition of 10 mm urea significantly expanded the fourfold channels of rmSSF while maintaining the integrity of the shell‐like structure (Figure 2c). On this structural foundation, introducing poly(α, L‐lysine) with a polymerization degree of 15 (PLL_15_) into the fourfold channels of rmSSF at pH 7.0, the ferritin cages self‐assembled into highly organized, well‐defined 2D square arrays, which were confirmed by transmission electron microscopy (TEM) (Figure 2c–e).^[^ ^55^ ^]^ Using ferritin as a model protein, the researchers demonstrated that electrostatic interactions between the protein surface and the PLL_15_ can guide the assembly of nanocages into highly organized 2D arrays. This study provided critical insights into how electrostatic forces can be manipulated to control the spatial arrangement of protein nanostructures, offering a robust method for the fabrication of novel biomaterials with tailored properties.^[^ ^56^ ^]^

### 2.2 π–π and Hydrophobic Interactions

**Figure 3:**
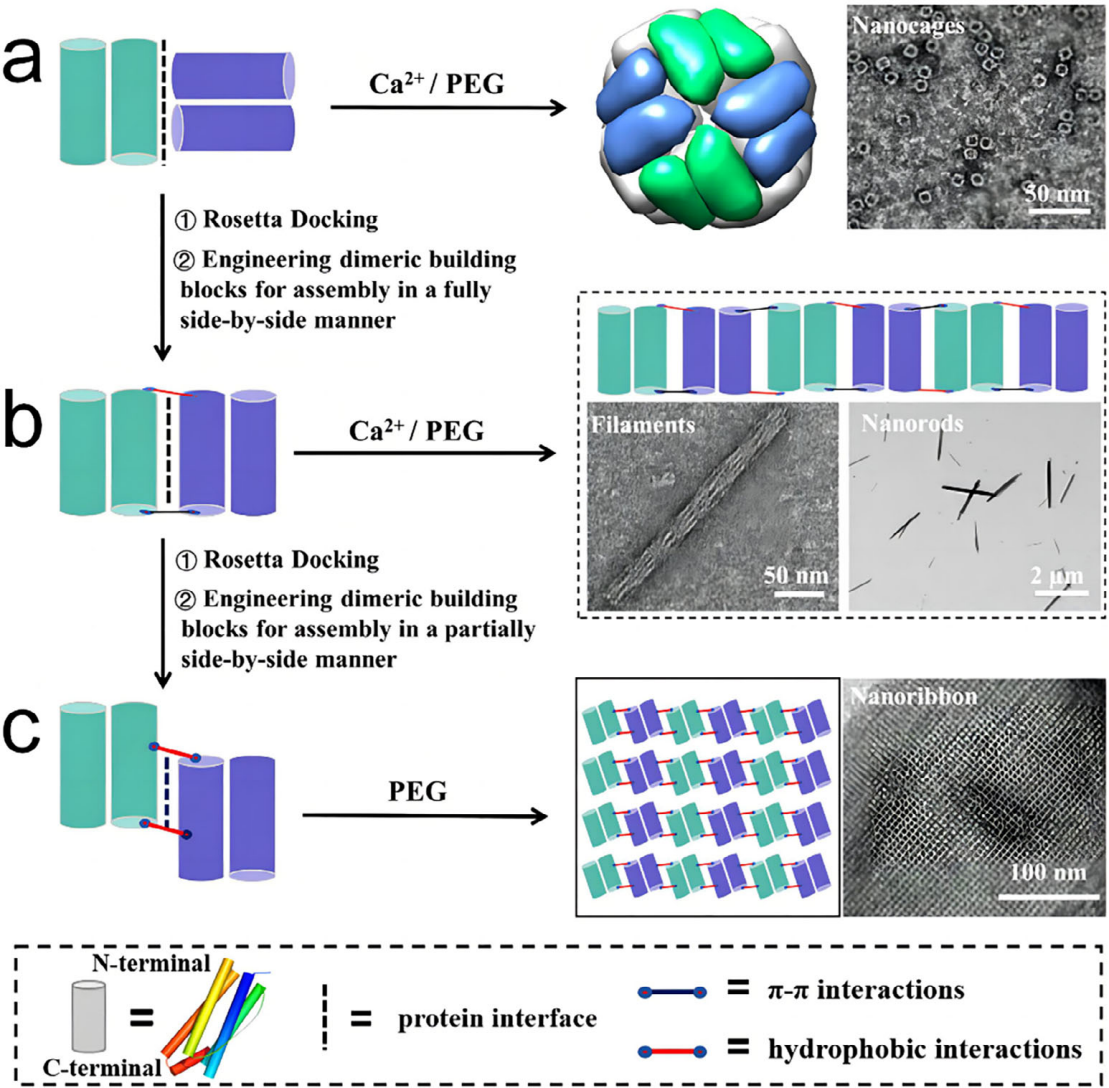
a) Naturally occurring dimeric thermotoga maritima ferritin (TmFtn) molecules interacting in a head‐to‐side manner to form 24‐meric protein nanocages in the presence of calcium ions and PEG. b,c) Rosetta docking and redesign of new PPIs between adjacent dimeric building blocks, transforming the assembly from hollow nanocages to nanofilaments, nanorods, or nanoribbons. Reproduced with permission.^[^ ^64^ ^]^ Copyright 2024, American Chemical Society.

Compared with electrostatic interactions, *π–π* stacking and hydrophobic interactions provide a more robust mechanism for the formation of stable and functional protein assemblies.^[^ ^57^, ^58^, ^59^ ^]^ *π–π* stacking interactions, which typically involve aromatic amino acids such as Phe (phenylalanine), Trp (tryptophan), and Tyr (tyrosine), offer strong and directional binding forces that facilitate the precise alignment of protein molecules into well‐organized structures.^[^ ^60^, ^61^ ^]^ Hydrophobic interactions further stabilize protein crystals by reducing the surface area in contact with water and enhancing the overall rigidity and integrity.^[^ ^62^ ^]^ More importantly, the synergy between these interactions leads to the formation of highly ordered and stable 2D arrays, where protein interfaces are redesigned and precisely positioned to optimize functional interactions.^[^ ^63^ ^]^ For example, Zhang et al. introduced an innovative protein interface redesign strategy that effectively transforms dimeric building blocks from hollow protein nanocages into diverse 1D and 2D nanostructures, including filaments, nanorods, and nanoribbons, mediated by *π–π* stacking and hydrophobic interactions.^[^ ^64^ ^]^ Thermotoga maritima ferritin, a naturally dimeric protein, typically forms 24‐mer hollow nanocages under the influence of calcium ions (**Figure** **3**a). By carefully redesigning the protein interface, the researchers were able to change the interaction from a head‐to‐side configuration to a fully side‐by‐side arrangement between adjacent dimeric units, resulting in the formation of a 1D filament (Figure 3b). This adjustment, combined with the addition of polyethylene glycol (PEG), facilitated the redirection of the self‐assembly process from hollow nanocages to the creation of 2D nanoribbons (Figure 3c). The resulting nanostructures exhibit a wide range of dimensions, from ≈50 to 4 µm. This versatile approach not only enables the controlled transformation of hollow protein nanocages into 1D or 2D nanomaterials, but also opens up new possibilities for constructing intricate nanoarchitectures.

**Figure 4:**
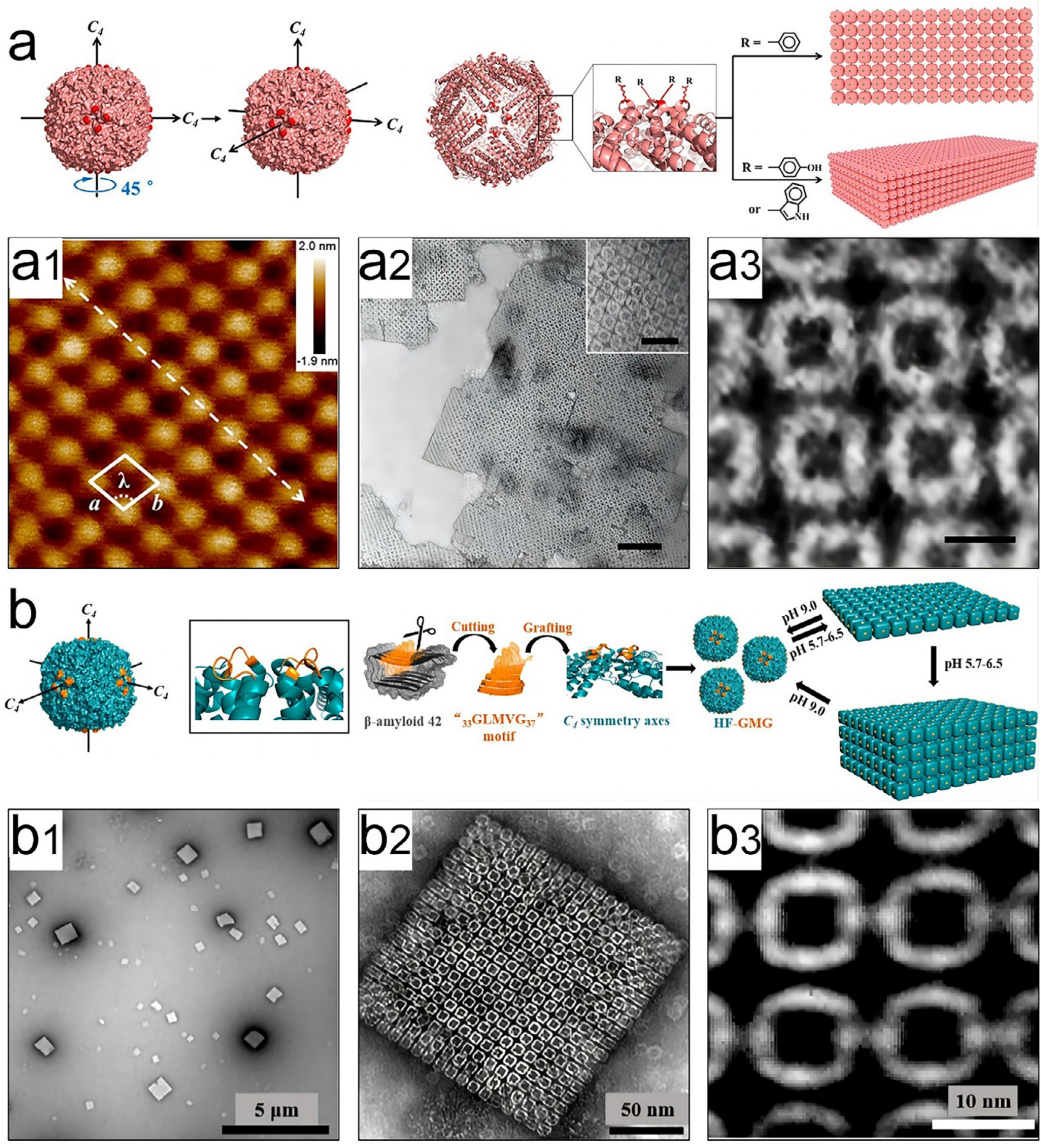
Schematic representation (a), AFM and TEM images (a1–a3) of the 24‐mer human H chain ferritin (rHuHF) superlattice formation. Reproduced with permission.^[^ ^15^ ^]^ Copyright 2018, American Chemical Society. Schematic representation (b) and TEM characterization (b1–b3) of 2D HF‐GMG arrays. Reproduced with permission.^[^ ^65^ ^]^ Copyright 2019, American Chemical Society.

Zhou et al. proposed an engineering strategy for fabricating 2D and 3D protein arrays.^[^ ^15^ ^]^ They demonstrated that substituting the single amino acids residue Glu162, located near the *C*_4_ rotation axes on the outer surface of the 24‐mer human H chain ferritin (rHuHF) nanocage, with Phe, Tyr, and Trp significantly influenced the self‐assembly process. Atomic force microscopy (AFM) and TEM validated that the specific substitution facilitated the formation of both 2D and 3D protein superlattices under varied solution salt concentrations and pH (**Figure** **4**a–a3). In these configurations, the protein cages are precisely aligned along the *C*_4_ axes, ensuring a defined spatial arrangement of neighboring ferritins. Building on these insights, Zheng and colleagues developed an effective method for fabricating 2D or 3D arrays by leveraging the hydrophobic interaction between the amyloidogenic motif GLMVG and the protein nanocages.^[^ ^65^ ^]^ The rHuHF mutants with GLMVG installed on the outer surface of the rHuHF nanocage near its *C*_4_ symmetry channels were named HF‐GMG. Initially, this strategy led to the formation of 2D nanocage arrays (Figure 4b–b3), but over time, highly ordered 3D arrays emerged. Importantly, the formation rate and reversibility of these arrays can be controlled by pH.

As mentioned above, it is also important to consider that *π–π* interactions are sensitive to ionic strength.^[^ ^66^ ^]^ Chen and colleagues chose a 24‐mer rHuHF nanocage as their starting material and mutated the original Asp (aspartic acid) residues near the *C*_3_ symmetry axes to Phe, creating a mutant known as 3FF.^[^ ^60^ ^]^ To investigate the effects of ionic strength on the assembly behavior of 3FF molecules in solution, they maintained the pH at 9.5 and conducted a comprehensive screening of salt concentrations. Under specific conditions (2 µm 3FF, 20 mm N‐cyclohexyl‐3‐aminopropanesulfonic acid, pH 9.5, 800 mm NaCl), the 3FF mutants self‐assembled into 2D equilateral‐hexagon‐like crystalline arrays within one hour. In contrast, wild‐type (WT) HuHF and other mutants failed to form such arrays under the same conditions, highlighting the critical role of the Phe‐mediated *π–π* interactions in the self‐assembly process. To elucidate the detailed atomic structure of the 2D nanocage arrays, they determined the crystal structure at a resolution of 2.72 Å. The structure exhibited a hexagonal packing arrangement. Consistent with their design, each 3FF molecule interacts with eight others along the *C*_3_ axes through Phe‐mediated *π–π* interactions in the crystalline form. These findings offer a novel design strategy for precise control over 2D protein self‐assembly, emphasizing the importance of *π–π* interactions in stabilizing these networks, especially in hydrophobic environments where aromatic residues play a significant role.

### 2.3 Covalent Bonding

**Figure 5:**
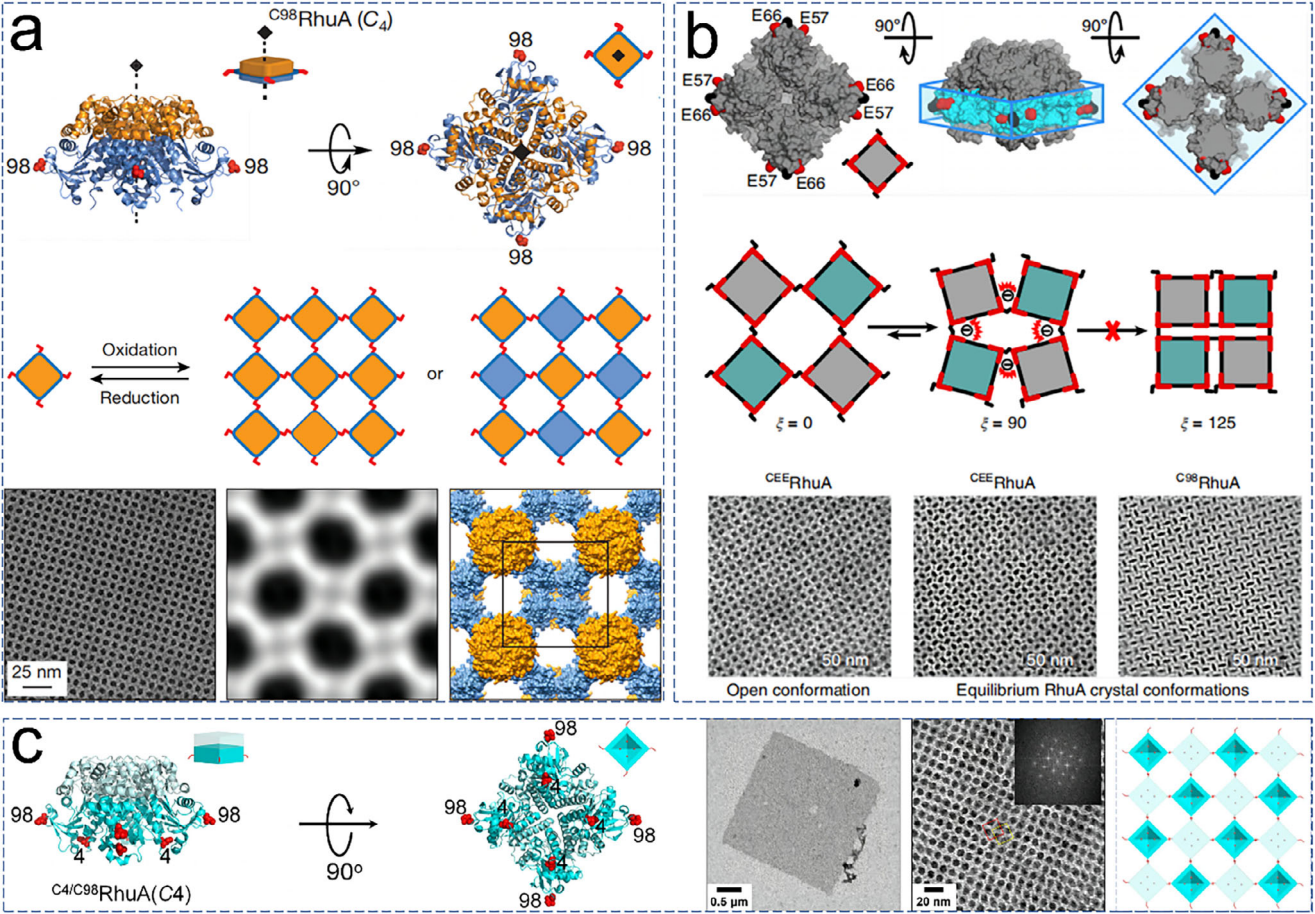
a) Schematic representations of design and self‐assembly of ^C98^RhuA, with TEM images of 2D ^C98^RhuA arrays. Reproduced with permission.^[^ ^41^ ^]^ Copyright 2016, Nature Publishing Group. b) Design and analysis of the ^CEE^RhuA construct, with TEM images showing the open and equilibrium states of 2D crystals. Reproduced with permission.^[^ ^71^ ^]^ Copyright 2018, Nature Publishing Group. c) Design of ^C4/C98^RhuA tetramers and their self‐assembly into 2D arrays, with TEM images. Reproduced with permission.^[^ ^19^ ^]^ Copyright 2019, American Chemical Society.

Covalent bonding plays a crucial role in protein assembly by providing a robust mechanism for linking protein molecules into highly ordered and stable structures.^[^ ^13^ ^]^ Methods such as disulfide bonding (S─S),^[^ ^67^ ^]^ native chemical ligation,^[^ ^68^ ^]^ Tyr dimerization,^[^ ^69^ ^]^ and sulfo‐NHS/EDC coupling^[^ ^70^ ^]^ are widely employed to ensure that assembled proteins maintain their native conformations and functional properties, making them ideal for applications that require high structural integrity and long‐term stability. Historically, a significant proportion of 2D protein assemblies have relied on covalent cross‐linking systems involving Cys (cysteine) residues, which can form reversible S─S bonds under redox control. For example, Suzuki and colleagues engineered a coherently dynamic, auxetic 2D protein array using S–S interactions in a variant of the RhuA (L‐rhamnulose‐1‐phosphate aldolase) protein with a dimension of 7 × 7 × 5 nm.^[^ ^41^ ^]^ By strategically introducing Cys residues at position 98, they established *C*_2_‐symmetric linkages, facilitating the formation of highly ordered 2D ^C98^RhuA arrays with *p*42_1_2 plane group symmetry through controlled oxidation (**Figure** **5**a). This crystal exhibited the largest array size, the lowest defect frequency, and dynamic array behavior. Furthermore, they explored an octameric *D*_4_‐symmetric ^F88/C98^RhuA variant, which also formed 2D arrays. Importantly, these 2D protein arrays were reversible under reducing conditions. Alberstein and colleagues built upon this foundation by creating new 2D ^CEE^RhuA (C98/E57/E66) protein arrays with chemically and mechanically switchable conformational dynamics and variable porosities. They mapped the free‐energy landscape of these arrays using all‐atom molecular dynamics simulations, validating that it is primarily governed by solvent reorganization entropy (Figure 5b).^[^ ^71^ ^]^ Our research group created a new RhuA mutant (^C4/C98^RhuA) with Cys residues at positions 98 and 4. This mutant self‐assembled into 2D crystalline arrays of micrometer dimensions via S─S bond formation (Figure 5c).^[^ ^19^ ^]^ The flexibility of these bonds enabled the formed arrays to expand and contract isotropically while maintaining the overall crystallinity. The Cys residues at position 4 were used to direct the de novo formation of micrometer arrays, highlighting the precision of covalent strategies in fabricating complex 2D assemblies. These methodologies underscored the precise control afforded by covalent strategies in creating intricate 2D protein assemblies.

**Figure 6:**
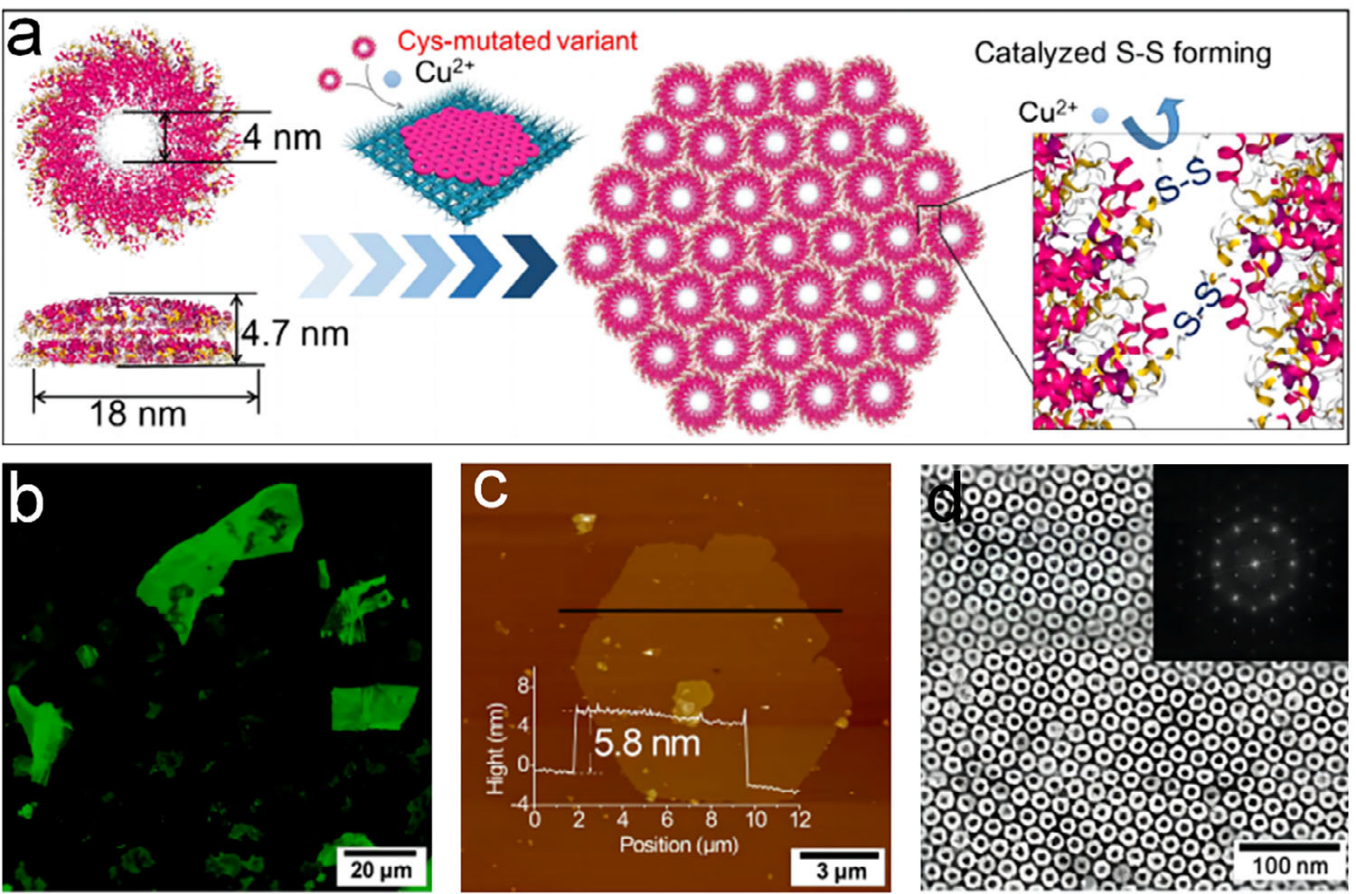
a) Schematic for the formation of single‐layer T103C‐TMV1, 3Cys nanosheets via Cu^2+^‐catalyzed disulfide bond formation. b) Fluorescent images, c) AFM images, and d) TEM images of the 2D TMV crystals, confirming the controlled assembly and structural characteristics. Reproduced with permission.^[^ ^78^ ^]^ Copyright 2018, American Chemical Society.

In addition to RhuA protein, TMV (tobacco mosaic virus) disks also served as exceptional building blocks.^[^ ^72^, ^73^ ^]^ Butler and colleagues first laid the groundwork for understanding the assembly mechanisms of TMV particles.^[^ ^74^ ^]^ Durham's team expanded on this by identifying various aggregation states that can be manipulated to form larger structures.^[^ ^75^ ^]^ With the advent of recombinant DNA technology, Bruckman and colleagues introduced a histidine tag (His‐tag) at the C‐terminus of the TMV coat protein (TMVCP), resulting in the creation of His‐TMVCP.^[^ ^76^ ^]^ This variant self‐assembles into diverse structures, including 2D hexagonally packed disk arrays, stacked disks, helical rods, fibers, and elongated rafts. The presence of the His‐tag significantly altered the self‐assembly behavior compared to the WT‐TMVCP. Besides His groups, our group further developed Cys‐based S─S bonds at the PPI of TMVCP, leading to a variety of TMV assemblies.^[^ ^77^ ^]^ T103C‐TMV1, 3Cys variant, with three Cys mutations (C1, C3 at the periphery, and C103 on the inner surface), exhibited various assembly behaviors depending on pH and ionic strength, forming 2D TMV disk arrays, disk stacks, and 3D TMV tube stacks and bundles. 2D arrays were assembled and observed under optimized conditions (pH 6.0 to 8.0, >200 mm phosphate buffer). Building on this work, Zhang et al. devised a simple strategy to construct more stable, ultra‐large, free‐standing, single‐layer 2D TMV nanosheets (**Figure** **6**a).^[^ ^78^ ^]^ Catalyzed by Cu^2+^, T103C‐TMV1,3Cys, generated 2D ultra‐large single‐layer TMV nanosheets with dimensions in tens of micrometers with ordered 4 nm pores (Figure 6b–d). Based on these features, ultrathin ultrafiltration membranes (thickness ≈ 40 nm) were fabricated using simple vacuum filtration for precise nano‐size separation. This study highlighted the versatility of the TMV disk in forming a diverse range of crystalline arrangements, demonstrating its potential as a generic building block for various nanoarchitectures.

**Figure 7:**
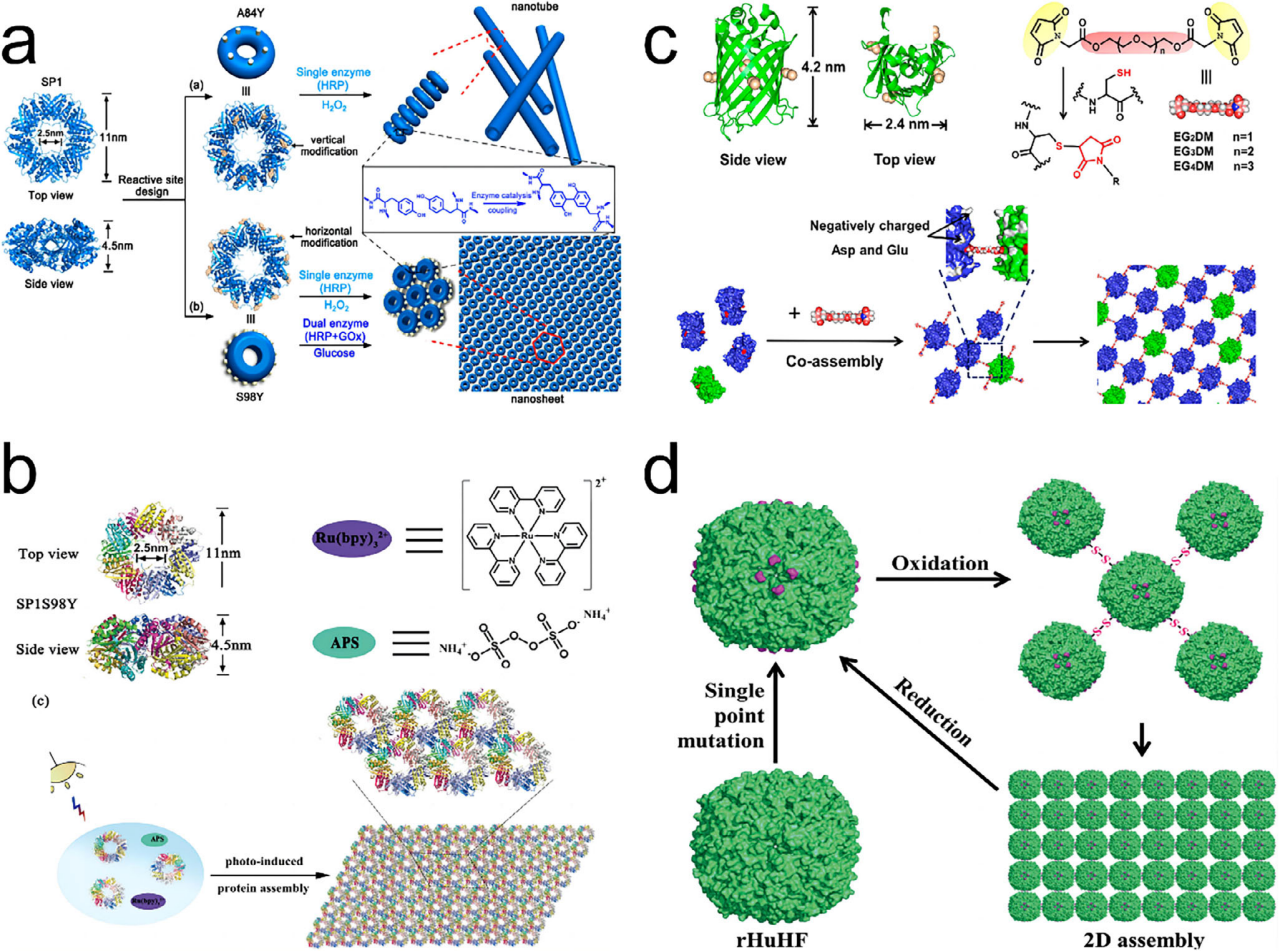
a) Formation of SP1 protein nanotubes or nanosheets via enzyme catalysis. Reproduced with permission.^[^ ^69^ ^]^ Copyright 2017, American Chemical Society. b) Photo‐induced coassembly of S98YSP1, [Ru(bpy)_3_]^2+^, and ammonium persulfate to construct 2D nanosheets. Reproduced with permission.^[^ ^79^ ^]^ Copyright 2018, Royal Society of Chemistry. c) Coassembly of EBFP2‐4C and EGFP‐4C to form 2D nanosheets through covalent‐induced self‐assembly. Reproduced with permission.^[^ ^80^ ^]^ Copyright 2019, American Chemical Society. d) Formation of 2D ferritin nanocage arrays. Reproduced with permission.^[^ ^81^ ^]^ Copyright 2019, Royal Society of Chemistry.

Another notable example of covalent cross‐linking was provided by Zhao and colleagues, who utilized the oxidative coupling of Tyr residues as an alternative strategy to produce 2D protein arrays.^[^ ^69^ ^]^ They chose the ring‐shaped protein SP1 (stable protein one) as a building block. Based on this assembly feature, their team introduced an S98Y mutation at the SP1 ring and performed the assembly. Using an enzyme‐based catalytic system containing horseradish peroxidase (HRP) and hydrogen peroxide (H_2_O_2_), or HRP and glucose oxidase (GOx), S98YSP1 mutants formed hexagonally packed 2D sheets spanning hundreds of nanometers (**Figure** **7**a).^[^ ^69^ ^]^ These sheets could stack into multilayered structures and be controlled by pH changes. To further extend this method, they introduced [Ru(bpy)_3_]^2+^ as a photosensitizer. In the presence of ammonium persulfate, S98YSP1 mutants were rapidly converted into covalently linked 2D nanosheets via the oxidative coupling of Tyr residues (Figure 7b).^[^ ^79^ ^]^ Inspired by these outstanding works, Li et al. used thiol‐reactive linkers and enhanced green fluorescent protein (EGFP) as building blocks. They redesigned EGFP to produce two mutants (EBFP2‐4C and EGFP‐4C) that could coassemble into 2D arrays through covalent‐induced self‐assembly (Figure 7c).^[^ ^80^ ^]^ The fluorescent chromophores EBFP2 (donor) and EGFP (acceptor) were well‐distributed and adopted a fixed orientation in the 2D arrays, endowing them with attractive light‐harvesting capabilities.

Globular proteins are also utilized for covalent cross‐linking. Zhou and his team introduced a disulfide‐mediated reversible self‐assembly strategy for constructing 2D protein nanocage arrays (Figure 7d).^[^ ^81^ ^]^ They site‐specifically mutated E162 in rHuHF to Cys, and named the mutants 4FC. Under solution conditions, 4FC protein nanocages assembled into regular 2D arrays. Importantly, by engineering specific S─S bonds, 2D rHuHF arrays could achieve reversible assembly and disassembly, offering a level of control unattainable with non‐covalent methods alone. Moreover, previous studies also showed that proteins could form long‐range 2D arrays at air‐phospholipid or air–water interfaces.^[^ ^82^, ^83^ ^]^ These studies highlight the critical role of covalent bonds in achieving precise spatial control over protein assemblies.

### 2.4 Metal‐Coordination

**Figure 8:**
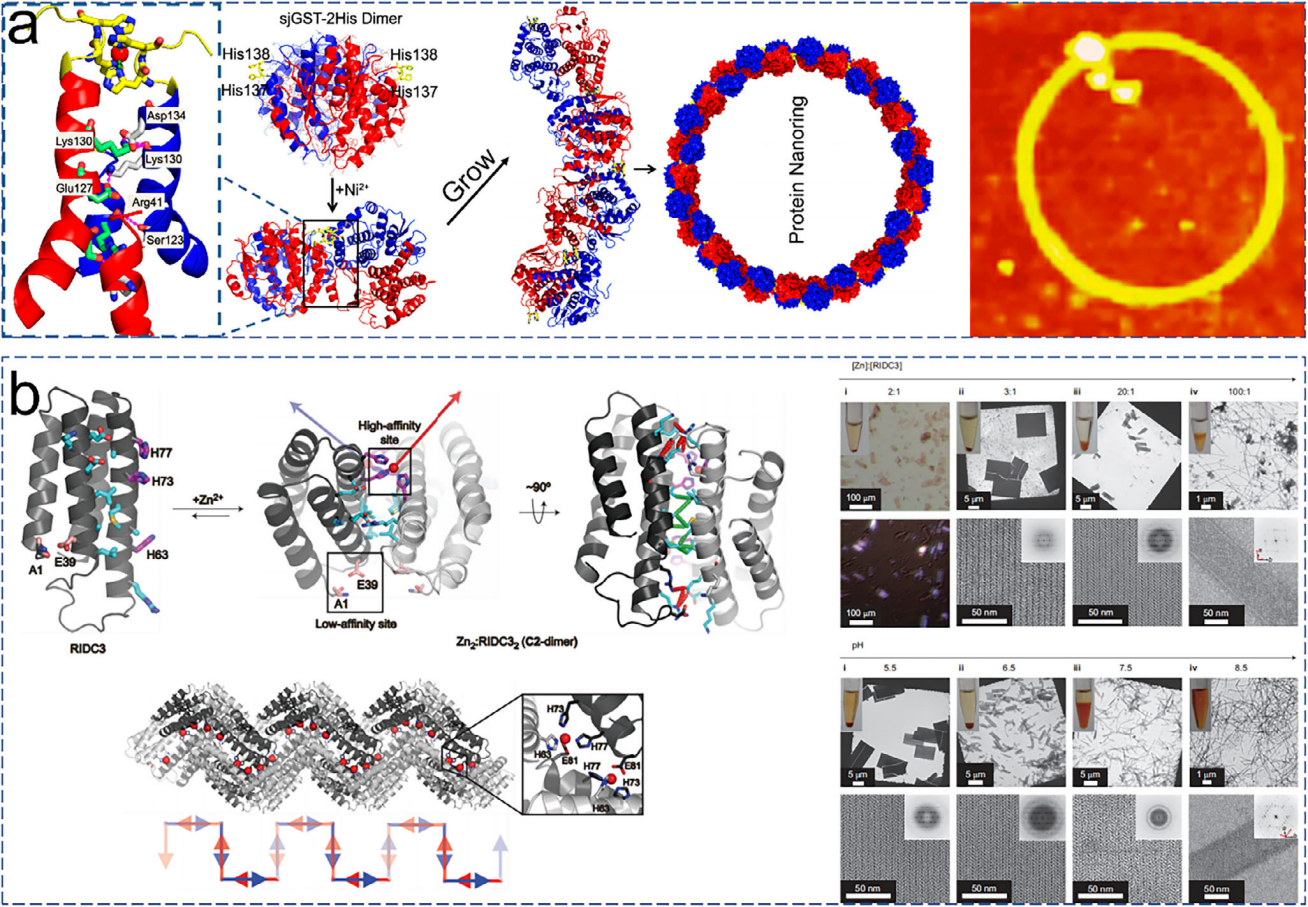
a) Ni^2+^‐induced assembly of 2His‐sjGST into 2D nanorings, with AFM characterization. Reproduced with permission.^[^ ^89^ ^]^ Copyright 2013, American Chemical Society. b) Scheme detailing the morphology changes of self‐assembled RIDC3 arrays under varying [Zn]: [RIDC3] ratios or pH conditions, with TEM images. Reproduced with permission.^[^ ^43^ ^]^ Copyright 2012, Nature Publishing Group.

Metal coordination strategies have rapidly evolved into a pivotal methodology for directing protein assembly.^[^ ^84^, ^85^, ^86^ ^]^ This technique exploits the inherent coordination chemistry of metals such as copper, zinc, and nickel, which can selectively bind to specific amino acid residues, primarily His and Cys, on protein surfaces.^[^ ^87^, ^88^ ^]^ These metal‐mediated interactions enable the construction of stable, well‐defined protein architectures, a capability that has proven invaluable in the development of functional 2D protein arrays. In a landmark study, Bai et al. showcased a sophisticated design approach to finely control the self‐assembly of a dimeric enzyme, glutathione transferase from *Schistosoma japonicum* (sjGST).^[^ ^89^ ^]^ By integrating steric control with metal coordination, they engineered a variant, sjGST‐2His, with two bis‐histidine motifs at its N‐terminus. This modification introduced symmetrical metal‐binding sites on the enzyme surface, aligned with the sjGST dimer's *C*_2_ axis. This strategic design biased the self‐assembly of sjGST into 2D nanorings in the presence of Ni^2+^ (**Figure** **8**a), highlighting the critical role of protein engineering in directing precise assembly behaviors. On the other hand, Brodin et al. explored the kinetic and thermodynamic aspects of 2D protein crystal formation through metal‐mediated interactions.^[^ ^43^ ^]^ Using a metal‐binding variant of cytochrome cb562 (RIDC3), they systematically varied pH and zinc ion concentration to determine the conditions favoring different assembly outcomes. At pH 8.5 and a high [Zn^2+^]: [RIDC3] ratio, nanotubes with diameters ranging from 80 to 15 µm were observed (Figure 8b). In contrast, lower ratios and more acidic conditions yielded 2D protein sheets. Their findings suggested a Zn^2+^‐dependent nucleation and growth mechanism, where small 2D nuclei served as the foundation for both 1D and 2D assemblies. Rapid nucleation under high zinc concentrations or high pH conditions favored the formation of helical nanotubes, whereas slower nucleation at lower concentrations or acidic pH led to the development of large 2D sheets. This study exemplified the tunable nature of metal‐induced assembly, demonstrating its potential to generate complex protein architectures with controlled dimensions and morphologies.

**Figure 9:**
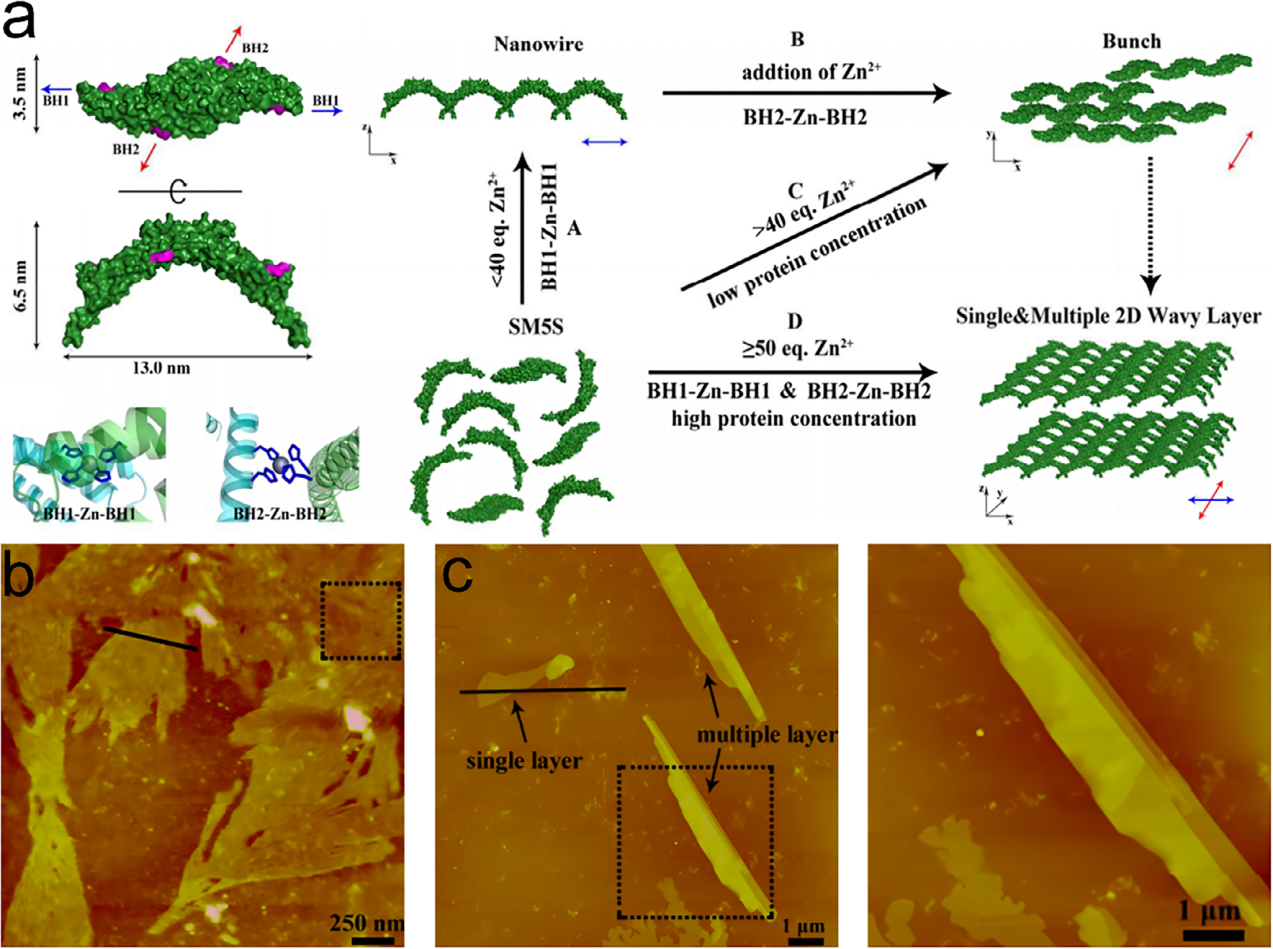
a) SM5S (SMAC‐Mutation5Stop) structure forms linear zigzag assemblies that further aggregate into 2D bundled nanowires, large 2D‐wavy single layers, and multiple layers. AFM images of 2D bundled nanowires (b) and large 2D‐wavy single layers (c). Reproduced with permission.^[^ ^91^ ^]^ Copyright 2013, Royal Society of Chemistry.

Subramanian et al. broadened the scope of metal‐mediated assembly by developing a hybrid DNA‐protein architecture using RIDC3 as a building block.^[^ ^90^ ^]^ They introduced a Cys residue onto RIDC3 to create ^C21^RIDC3 mutants capable of specifically binding short DNA strands. Protein–DNA conjugates and complementary DNA sequences were synthesized and coassembled under varying conditions. Under limited conditions, these conjugates formed ordered arrays. Subsequently, tridentate Zn^2+^ coordination further drove the formation of a corrugated 2D sheet in an antiparallel fashion. This study underscored the complexity and precision achievable through the integration of DNA and metal‐coordination strategies in protein assembly. Qiao et al. provided additional insights into the interplay between metal coordination and protein structure in their work on Zn^2+^‐mediated self‐assembly of a designed V‐shaped protein, SMAC.^[^ ^91^ ^]^ By installing bis‐His motifs on each monomer, they achieved a quadrilateral in‐plane orientation during dimerization. These motifs exhibited different binding affinities for Zn^2+^ ions. At lower Zn^2+^ concentrations, the higher‐affinity motifs (H75/H79) were preferentially saturated, leading to the formation of nanowires. Higher Zn^2+^ concentrations were necessary to occupy the lower‐affinity motifs (H137/H141), which in turn induced the formation of wavy 2D sheets (**Figure** **9**a–c). This work detailed the intricate relationship between metal coordination and protein conformation, emphasizing the potential for precise control over the assembly process and the creation of complex, dynamic nanostructures.

**Figure 10:**
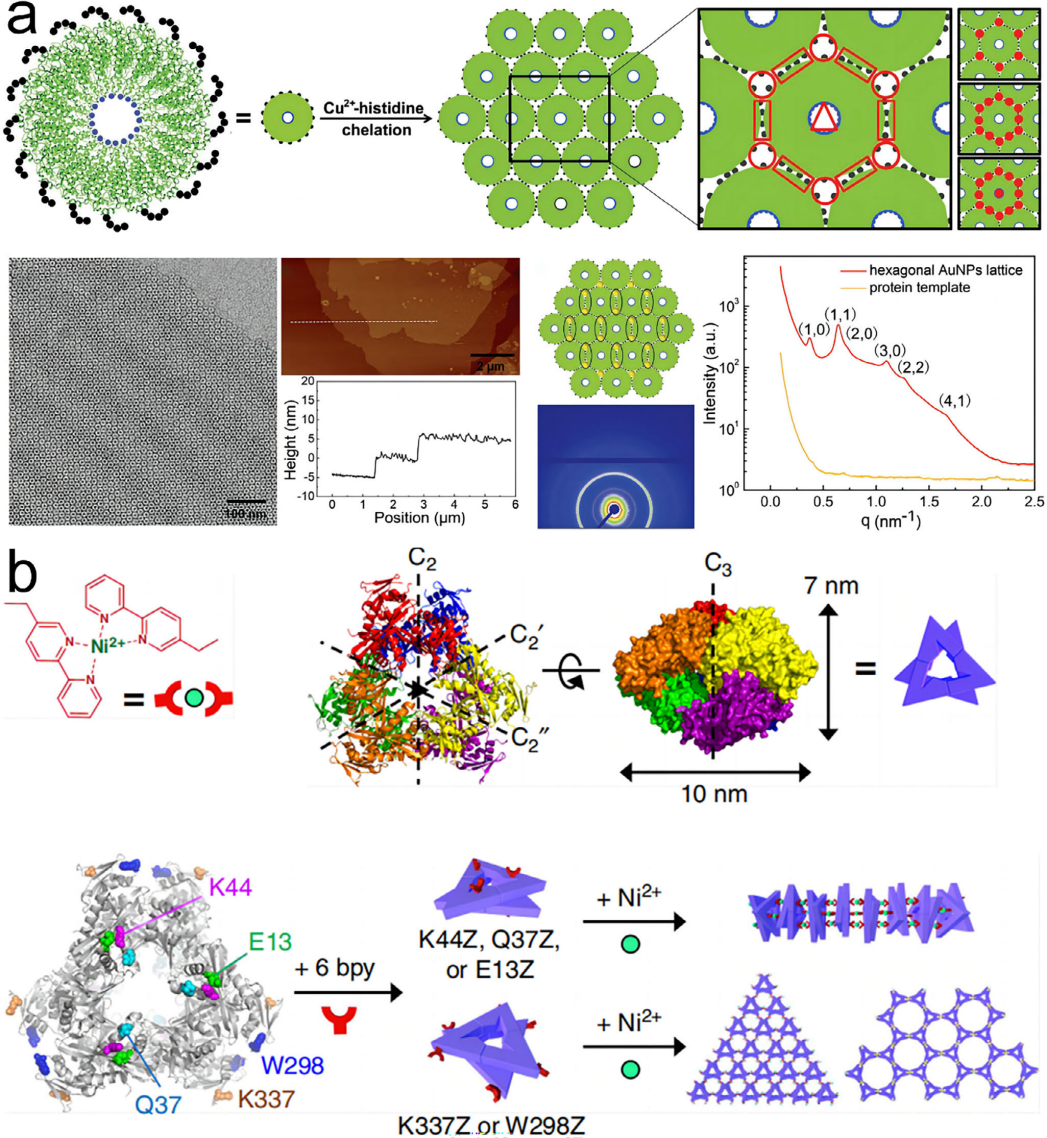
a) Self‐assembly of highly ordered 2D T103C‐TMV‐4His monolayer sheets via Cu^2+^‐His interactions, with TEM, AFM, and SAXS characterizations of the 2D TMV monolayer sheet. Reproduced with permission.^[^ ^92^ ^]^ Copyright 2019, Wiley‐VCH Verlag GmbH and Co. KGaA, Weinheim. b) Scheme for 1D assembly and 2D assembly via [Ni(bpy)_2_]^2+^ formation depending on the specific mutation sites. Reproduced with permission.^[^ ^42^ ^]^ Copyright 2019, Nature Publishing Group.

More recently, our research group developed a simple yet effective strategy for assembling 2D periodic arrays based on Cu^2+^‐His interactions.^[^ ^92^ ^]^ The T103C‐TMV‐4His variant, featuring a Cys residue at position 103 and a C‐terminal 4His‐tag, was constructed. Well‐ordered 2D monolayer sheets were formed on a Cu(OH)_2_ nanowire‐coated mesh,^[^ ^93^ ^]^ validating that Cu^2+^‐His interactions consistently drive the self‐assembly of TMV disks into 2D protein arrays (**Figure** **10**a). TEM, AFM, and Small Angle X‐ray Scattering (SAXS) images showed macroscopic 2D TMV crystals with highly ordered spatial structures, a honeycomb pattern, and sizes spanning several to tens of micrometers. Additionally, Yang et al. proposed a novel approach to achieve diverse protein assemblies driven by metal ions and chelating amino acids.^[^ ^42^ ^]^ They genetically incorporated an unnatural chelating amino acid, bipyridine‐alanine (bpy‐Ala), to facilitate the assembly of 1D and 2D structures (Figure 10b). The selective formation of [Ni(bpy‐Ala)_2_]^2+^ complexes enabled the creation of a wide array of nanostructures, including linear arrays and 2D protein arrays.

### 2.5 Receptor–Ligand Recognition

Receptor–ligand recognition, genetic fusion, and computational design leverage sophisticated molecular recognition mechanisms and inherent biological interactions to promote the spontaneous assembly of proteins.^[^ ^94^, ^95^ ^]^ A key advantage of these strategies is their dependence on well‐documented PPIs that are characterized by high symmetry, specificity, and affinity.^[^ ^1^, ^94^, ^95^ ^]^ These interactions allow for precise control over protein binding and organization, enabling the creation of highly ordered and functional arrays. Furthermore, the integration of computational design methodologies with biological approaches significantly advances innovation and development in this field.^[^ ^96^, ^97^, ^98^ ^]^ This synergy not only increases the functional complexity of the materials but also enhances their adaptability to environmental changes. Additionally, this method avoids the need for harsh chemical conditions or costly reagents, thereby improving sustainability and economic viability.^[^ ^99^, ^100^ ^]^

Receptor–ligand recognition strategies offer precise control over interactions by designing well‐defined, functional structures.^[^ ^101^, ^102^, ^103^, ^104^ ^]^ This approach leverages the specific binding between receptors and ligands, which can be engineered to exhibit high affinity and specificity.^[^ ^105^ ^]^ By strategically designing these interactions, researchers can induce the formation of stable assemblies with predictable orientations and spacings, thereby enabling the fabrication of complex protein architectures.^[^ ^106^, ^107^ ^]^ The precision of receptor–ligand interactions allows for fine‐tuning assembly conditions to optimize the formation of 2D protein arrays. For instance, Ringler et al. pioneered this approach by exploring the self‐assembly of proteins into designed networks.^[^ ^39^ ^]^ They focused on the RhuA protein, a compact homotetramer with *C*_4_ symmetry. Through strategic site‐specific mutations at positions N133C, K261C, and C126S, they introduced Cys residues that were subsequently biotinylated, enabling directional binding to four streptavidin units, each with *C*_2_ symmetry. This resulted in the bRS4 structure, which facilitated the formation of small 2D arrays, typically limited to ≈50 × 50 nm. The spatial confinement observed in these arrays highlights the initial challenges in achieving larger structures. However, in a lipid monolayer interface, researchers found that bR extended the array width up to 200 nm.

**Figure 11:**
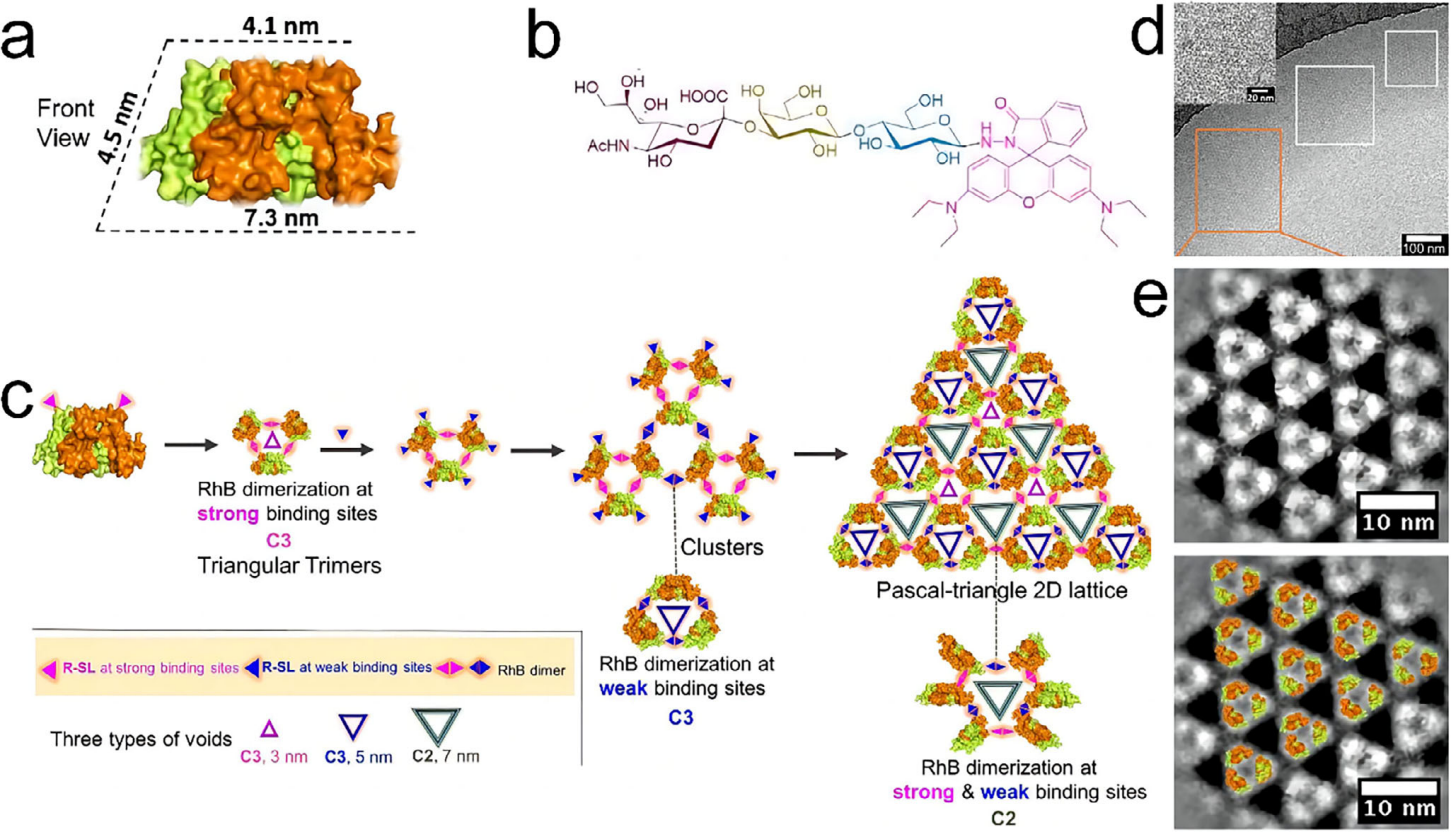
a) Structure of the wheat germ agglutinin (WGA) dimer with carbohydrate‐binding sites at the tetrahedral vertices. b) The molecular structure of inducing ligand (R‐SL) containing α2,3‐sialyllactose and rhodamine B (RhB). c) Proposed mechanism for WGA assembly with RhB. d) Cryo‐EM images of WGA 2D arrays with insets showing enlarged views. e) Overlay of the structural model on cryo‐EM images. Reproduced with permission.^[^ ^109^ ^]^ Copyright 2020, Wiley‐VCH Verlag GmbH and Co. KGaA, Weinheim.

Building on these foundational studies, Kitagishi et al. introduced a novel approach by exploiting heme–heme pocket interactions for protein assembly.^[^ ^108^ ^]^ Their methodology began with the polymerization of cytb562 (H63C) mutants into linear 1D systems. This initial step was then extended to generate 2D branched networks via the interaction of heme–hemoprotein pairs. Liu et al. further fabricated a Pascal triangle lattice of proteins.^[^ ^109^ ^]^ In this study, α2,3‐sialyllactose and rhodamine B (RhB) were covalently conjugated and named R‐SL, which served as the inducing ligand (**Figure** **11**a,b). WGA (wheat germ agglutinin), with four strong binding sites on its top face and four weak binding sites on its bottom face, was used as the building block. Initially, three WGA proteins were bound via RhB dimerization at the top sites, followed by the formation of 2D lattices through R‐SL ligand binding at the bottom sites, ultimately yielding the desired Pascal triangle lattice structure (Figure 11c–e).

**Figure 12:**
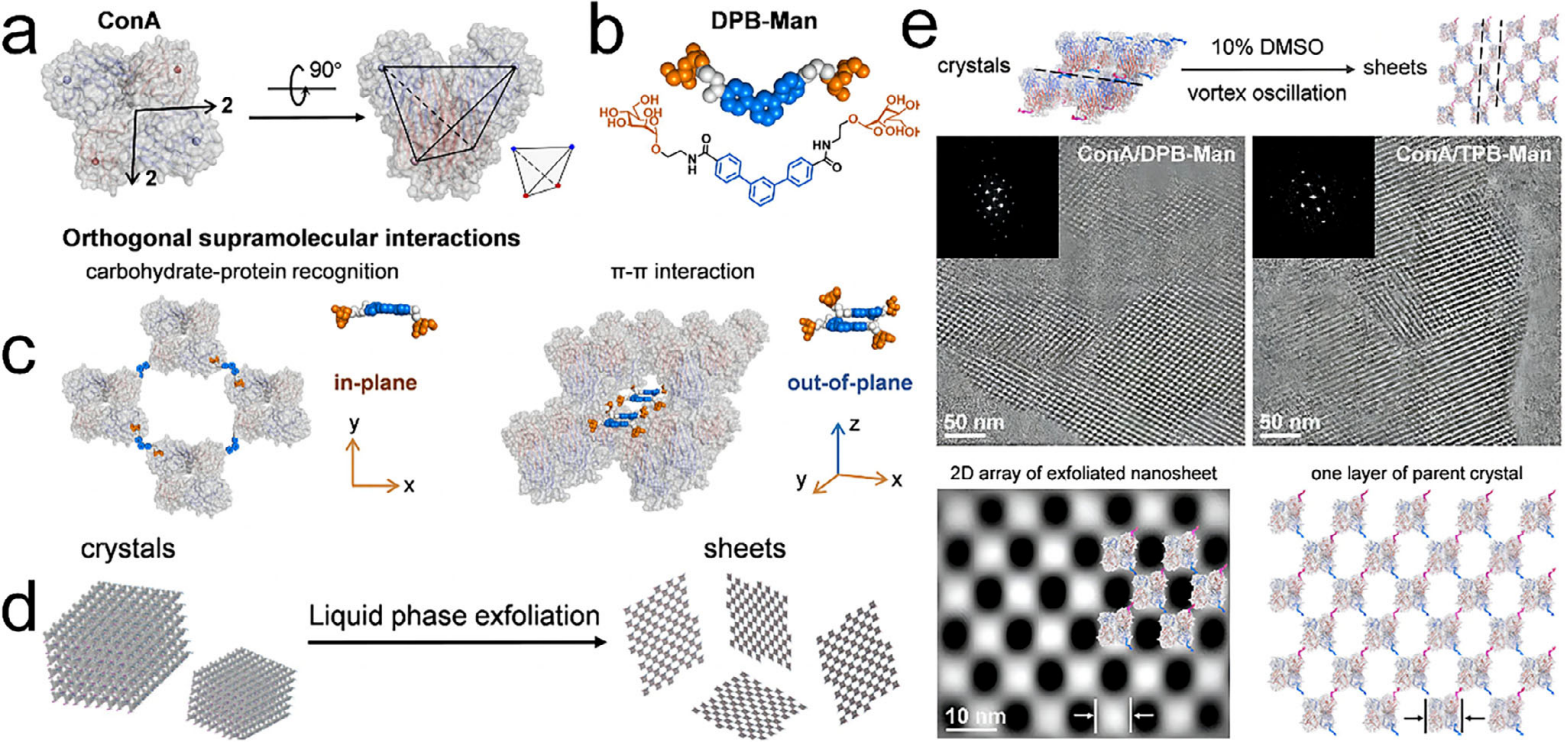
a) Representative views of *D*_2_ symmetric concanavalin A (ConA) with near‐tetrahedral geometry and four carbohydrate‐binding sites. b) Chemical structure and spherical model of ligand DPB‐Man. c) Illustration of layered ConA crystals generated by orthogonal supramolecular interactions. d) Schematic for the liquid phase exfoliation of layered protein crystals, facilitating the formation of 2D nanosheets. e) Cryo‐EM images of ConA/DPB‐Man and ConA/TPB‐Man nanosheets. Reproduced with permission.^[^ ^110^ ^]^ Copyright 2024, American Chemical Society.

Li and colleagues innovatively applied the liquid phase exfoliation technique to concanavalin A (ConA) crystals, successfully generating 2D sheets.^[^ ^110^ ^]^ ConA, a *D*_2_ symmetric protein with a near‐tetrahedral structure and carbohydrate‐binding sites at the tetrahedral vertices served as the fundamental building block (**Figure** **12**a), while a redesigned molecule. DPB‐Man (an aromatic core of dipheylbenzene with two mannoside groups) was used as the ligand (Figure 12b). Under optimized conditions, ConA crystals were formed through orthogonal supramolecular interactions, where the recognition between protein and carbohydrate molecules drove the assembly of 2D layers, while *π–π* stacking interactions were responsible for the stacking of these layers (Figure 12c). To dissociate the *π–π* stacking and produce 2D sheets, the researchers employed a vortex oscillation technique in a 10% (v/v) DMSO aqueous solution and an acidic buffer. This method effectively disrupted the interlayer *π–π* interactions, allowing the exfoliation of the crystal into 2D sheets (Figure 12d). The analysis of 2D class averages revealed that the protein arrangements in the exfoliated nanosheets were consistent with those observed in the layers formed by protein–carbohydrate interactions within the crystals. Significantly, the exfoliated 2D sheets were several orders of magnitude larger than self‐assembled nanosheets, leading to enhanced intrinsic bioactivity of the building blocks. The larger surface area facilitated more efficient receptor clustering, thereby improving biological functionality and reducing the rate of endocytosis.

**Figure 13:**
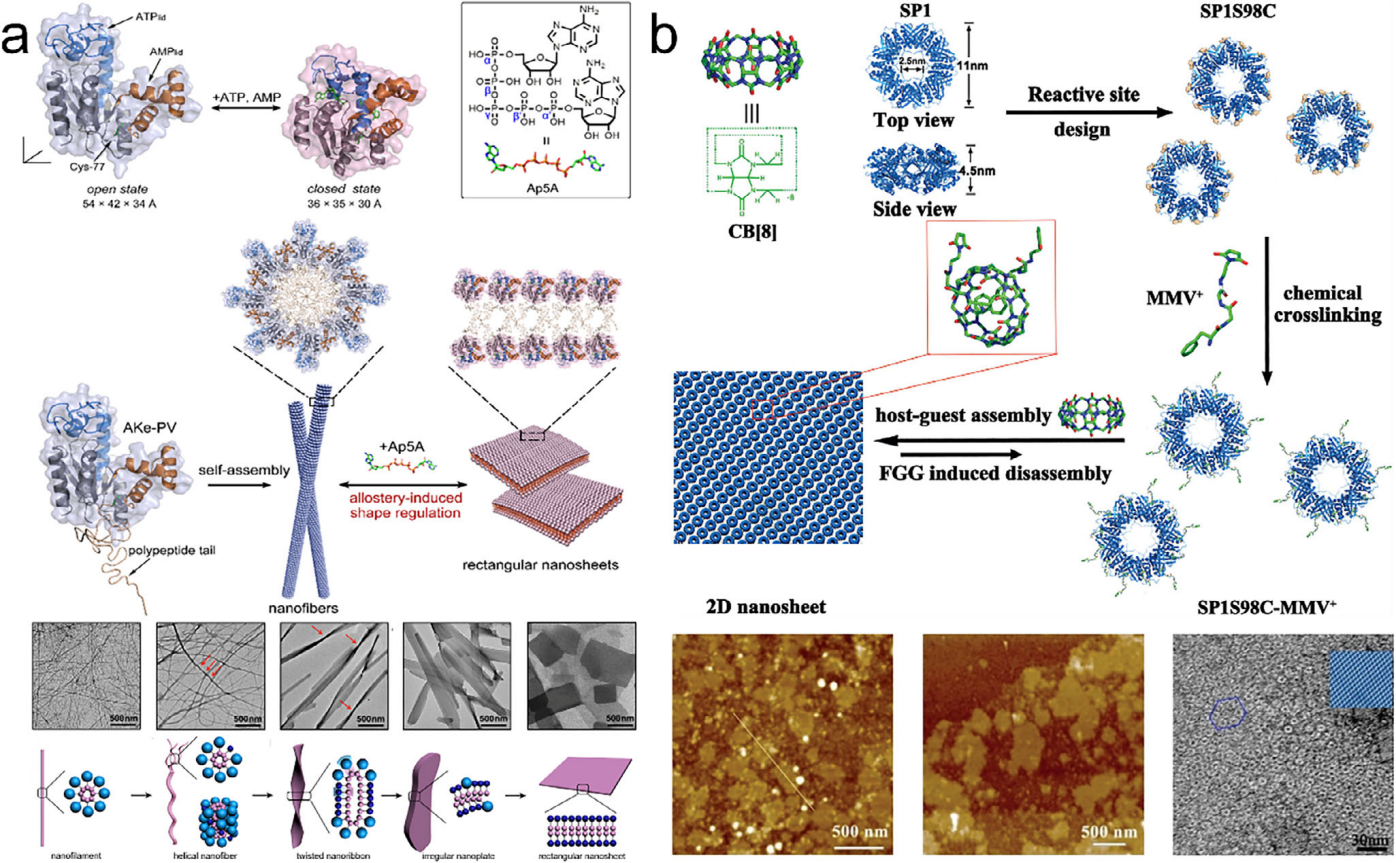
a) Allosteric activation leading to the shape transition of AKe‐based protein amphiphiles from 1D nanofilaments to 2D rectangular nanosheets, with  the corresponding TEM images. Reproduced with permission.^[^ ^111^ ^]^ Copyright 2019, American Chemical Society. b) Host–guest interactions driving the assembly of 2D SP1S98C nanosheets, with AFM and TEM images of the protein nanosheets. Reproduced with permission.^[^ ^112^ ^]^ Copyright 2021, Royal Society of Chemistry.

Xu et al. achieved dynamic manipulation of the shape transformation of protein assemblies.^[^ ^111^ ^]^ Their focus was on exploring the allosteric regulation of adenylate kinase (AKe) assemblies, specifically their transition from 1D nanofilaments to 2D rectangular nanosheets. Unlike structurally stable building blocks, they leveraged the allosteric ligand‐binding properties of AKe to design a ligand‐dependent assembly system capable of switching configurations. AKe's ability to recognize adenosine triphosphate (ATP) and adenosine monophosphate (AMP) induces a conformational shift from an open to a closed state, thereby enabling a phosphor transfer reaction. Ap5A (diadenosine‐5‐pentaphosphate) was used as a ligand capable of mimicking the function of ATP and AMP. In its open state, driven by the hydrophobic effect, the AKe amphiphile self‐assembled into extended 1D fibers. Upon the introduction of Ap5A, these 1D assemblies transformed into well‐ordered 2D sheets (**Figure** **13**a). This transition was accompanied by a significant conformational change in AKe from the open state to the closed state.

Li et al. demonstrated control over the growth of SP1S98C (SP1 with a Cys mutation at the 98th site) into nanosheets by harnessing receptor–ligand interactions, underscoring the versatility and tunable nature of these protein‐based nanostructures.^[^ ^112^ ^]^ By covalently attaching an analog of methyl viologen namely MMV^+^ to its lateral surface, and leveraging the specific recognition capabilities of MMV^+^ to cucurbit[8]uril (CB[8]), the mutant protein SP1S98C was manipulated to grow isotropically into highly ordered 2D nanosheets (Figure 13b). Through meticulous control of assembly conditions, the size of these 2D nanosheets could be accurately regulated. The competitive binding ability of MMV^+^ to CB[8] facilitated the disassembly of these nanosheets into free SP1S98C motifs upon the addition of ferrocene‐terminated glycol (FGG). Notably, the reintroduction of CB[8] led to the reformation of the assemblies, thus establishing a reversible 2D protein assembly‐disassembly system.

**Figure 14:**
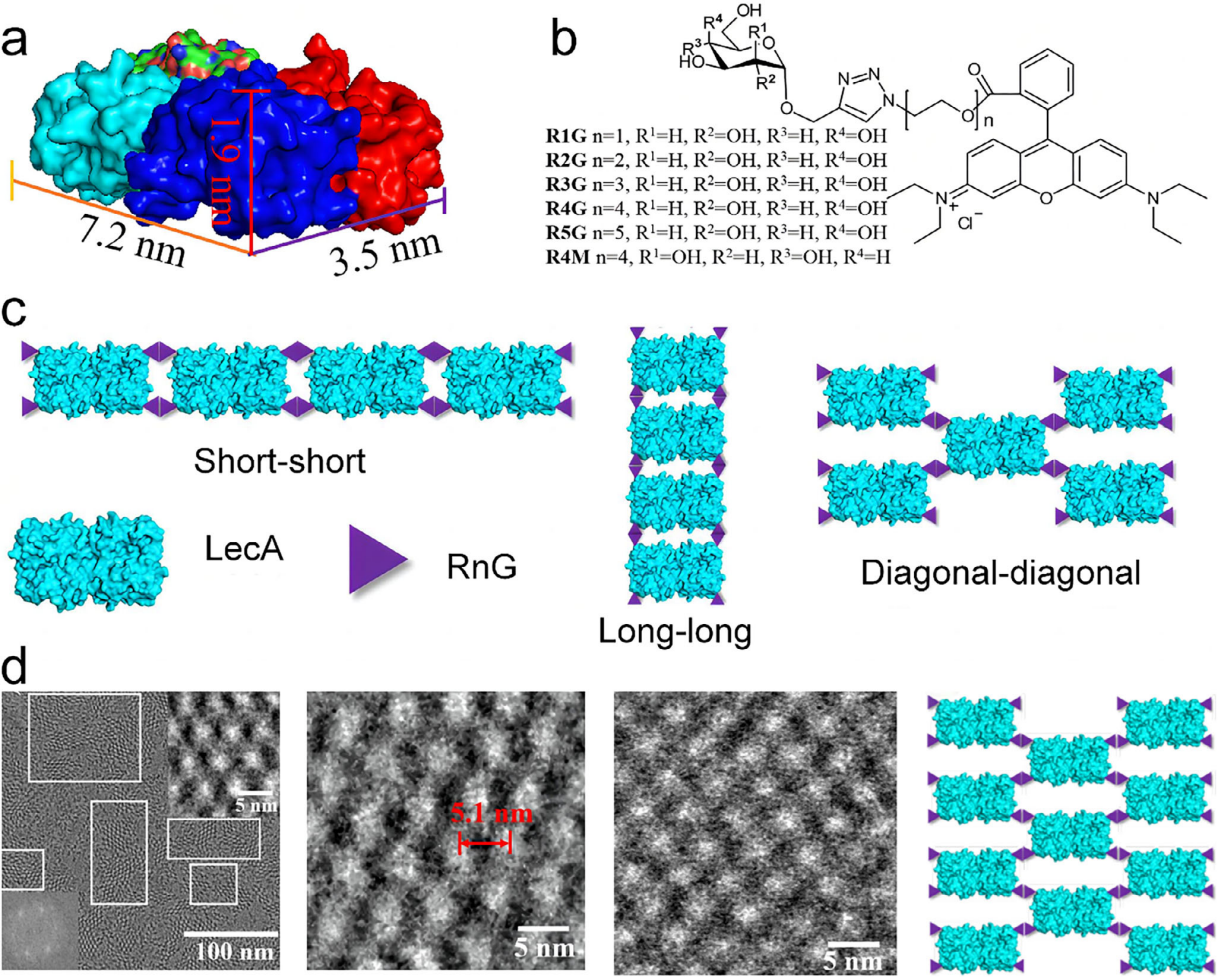
a) Structure of the tetrameric protein lectin A (LecA). b) Chemical structures of ligands RnG (*n* = 1 to 5). c) Three packing patterns of LecA/RnG based on the dimerization of RnG. d) Enlarged cryo‐EM images and schematic representation from LecA/R2G to LecA/RnG 2D lattices. Reproduced with permission.^[^ ^16^ ^]^ Copyright 2017, Wiley‐VCH Verlag GmbH & Co. KGaA, Weinheim.

While these methods have relied on the selective binding of single receptor–ligand interactions within specific proteins, they have also highlighted the inherent limitations in developing 2D array assemblies due to the complexity of such interactions. To overcome these challenges, Sakai et al. introduced the concept of dual supramolecular interactions (protein–sugar interactions and *π–π* stacking) to direct the formation of 3D protein crystals.^[^ ^113^ ^]^ This approach provided insights into how multiple binding motifs could be synergistically employed to achieve complex protein architectures. Yang et al. extended this strategy to create 1D nanoribbons, nanowires, and 2D nanosheets using lectin and sugar interactions, along with *π–π* stacking.^[^ ^16^ ^]^ They selected lectin A (LecA) and inducing ligands RnG (a covalent cross‐link of RhB and ethylene oxide and galactopyranoside) as the assembly building block (**Figure** **14**a,b). By varying the length (*n*) of the ethylene oxide tether in RnG (Figure 14c), they obtained three distinct extended LecA structures: 1D nanoribbons, 2D sheets, and 3D layered structures via dual supramolecular interactions. Among these, 2D sheets were obtained with R2G, R4G, and R5G, leading to a diagonal–diagonal protein packing arrangement (Figure 14d). The authors used a combination of molecular simulations and experimental validation to demonstrate that the tether length of assembly‐inducing ligands could modulate the self‐assembly into multidimensional structures, highlighting the significant influence of ligand properties on assembly morphology.

### 2.6 Genetic Fusion

**Figure 15:**
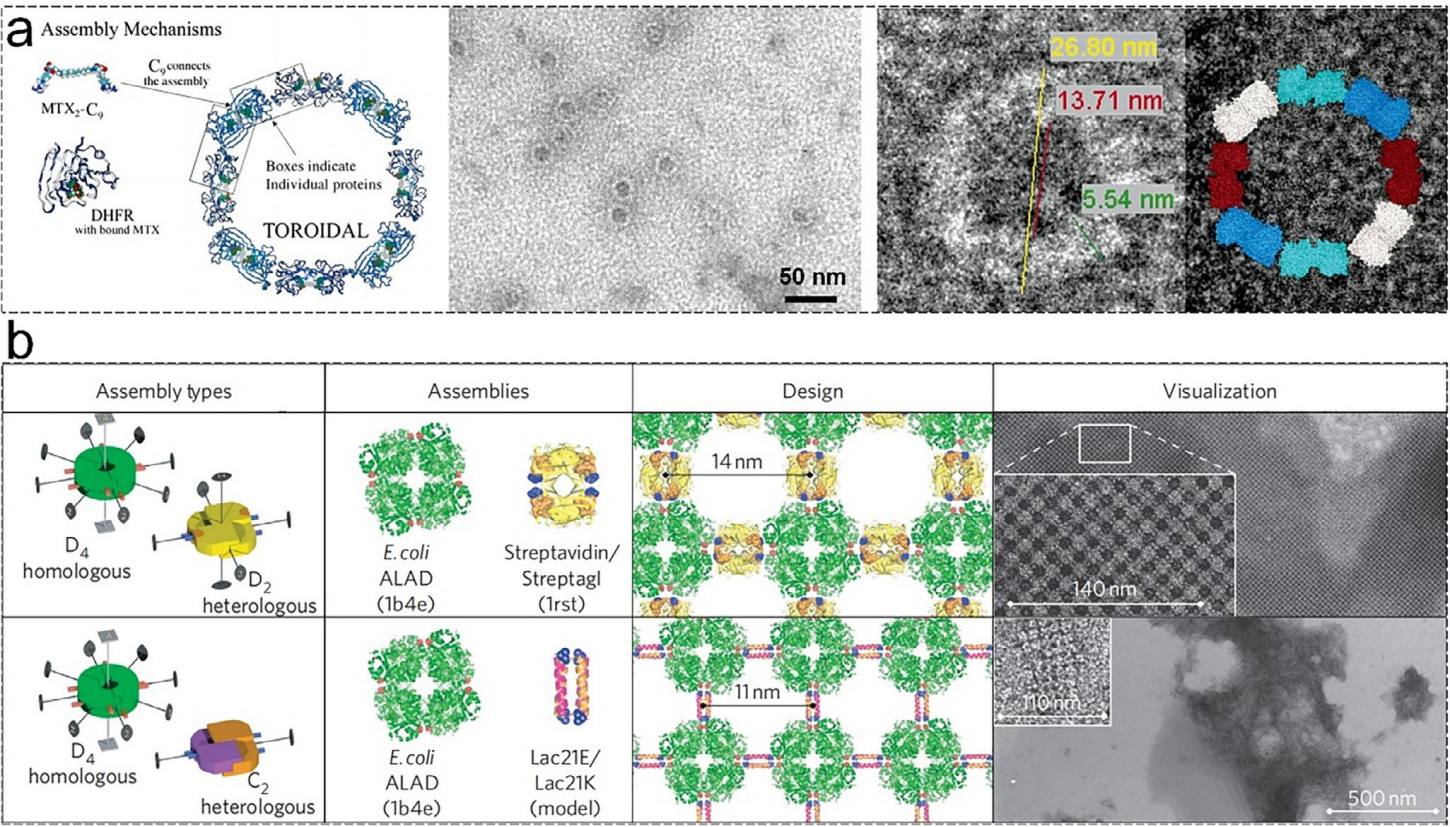
a) Self‐assembly of 2D DHFR arrays along the *C*_2_ symmetry axis shared by the components, enabling precise control over the arrangement and interactions. Reproduced with permission.^[^ ^119^ ^]^ Copyright 2006, American Chemical Society. b) Coassembly of homologous *D*_4_‐symmetric ALAD building blocks with heterologous *D*_2_‐symmetric streptavidin/Streptag I or heterologous *C*_2_‐symmetric Lac21E/Lac21K, demonstrating the versatility of genetic fusion in creating complex protein architectures. Reproduced with permission.^[^ ^38^ ^]^ Copyright 2019. Nature Publishing Group.

Genetic fusion strategies exemplify how structural symmetry can be utilized to construct 2D protein arrays.^[^ ^114^ ^]^ Symmetric proteins, due to their highly symmetric structures and energetically favorable interactions, are particularly well‐suited for such assemblies.^[^ ^115^, ^116^ ^]^ When monomeric proteins are genetically fused to a functional motif, they often exhibit geometric symmetry, enabling the formation of higher‐order symmetrical structures that meet specific physicochemical requirements.^[^ ^117^, ^118^ ^]^ The uniform distribution of functional elements at the nanoscale, achieved through the integration of different functional modules, enhances both the diversity and functionality of these arrays.^[^ ^26^ ^]^ Carlson et al. used dihydrofolate reductase (DHFR) molecules fused by a flexible peptide linker to investigate chemically controlled self‐assembly of 2D protein nanorings (**Figure** **15**a).^[^ ^119^ ^]^ Their study provided foundational insights into the noncovalent interactions that drive protein assembly. Sinclair et al. further demonstrated the potential of manipulating proteins with different rotational symmetries to control structural outcomes.^[^ ^38^ ^]^ They employed *D*_2_‐symmetric streptavidin/streptag I, *D*_4_‐symmetric aminolevulinic acid dehydrogenase (ALAD), and *C*_2_‐symmetric Lac21E/Lac21K proteins to construct two types of ordered 2D arrays through symmetry‐based design principles (Figure 15b). By fusing proteins along shared symmetry axes, they reduced the degrees of freedom, thereby enhancing the predictability and uniformity of the assemblies. ALAD was fused to the Streptag I peptide and coassembled either with streptavidin or with Lac21E/Lac21K. The resulting 2D protein arrays exhibited variable unit cell dimensions, which could be fine‐tuned by altering the length of the linking peptide. This study marked a critical step forward by integrating symmetry considerations into the design of 2D protein arrays.

Building on the concept of rotational symmetry matching, Poulos et al. expanded these ideas by exploring symmetry‐based assembly.^[^ ^120^ ^]^ They genetically engineered a TTT‐FUR fusion protein to ensure precise alignment and interaction, forming ordered 2D structures where the ferric uptake regulator (FUR) domains maintain the connections among polymerized TTT (three Tel‐SAM domains) fibers. This research emphasized the importance of symmetry and genetic design in achieving high‐order 2D protein arrays, building on the principles outlined in previous studies.^[^ ^121^, ^122^, ^123^, ^124^ ^]^ The progression from understanding basic noncovalent interactions to developing comprehensive design rules highlights the maturation of the field.

### 2.7 Computational Design

**Figure 16:**
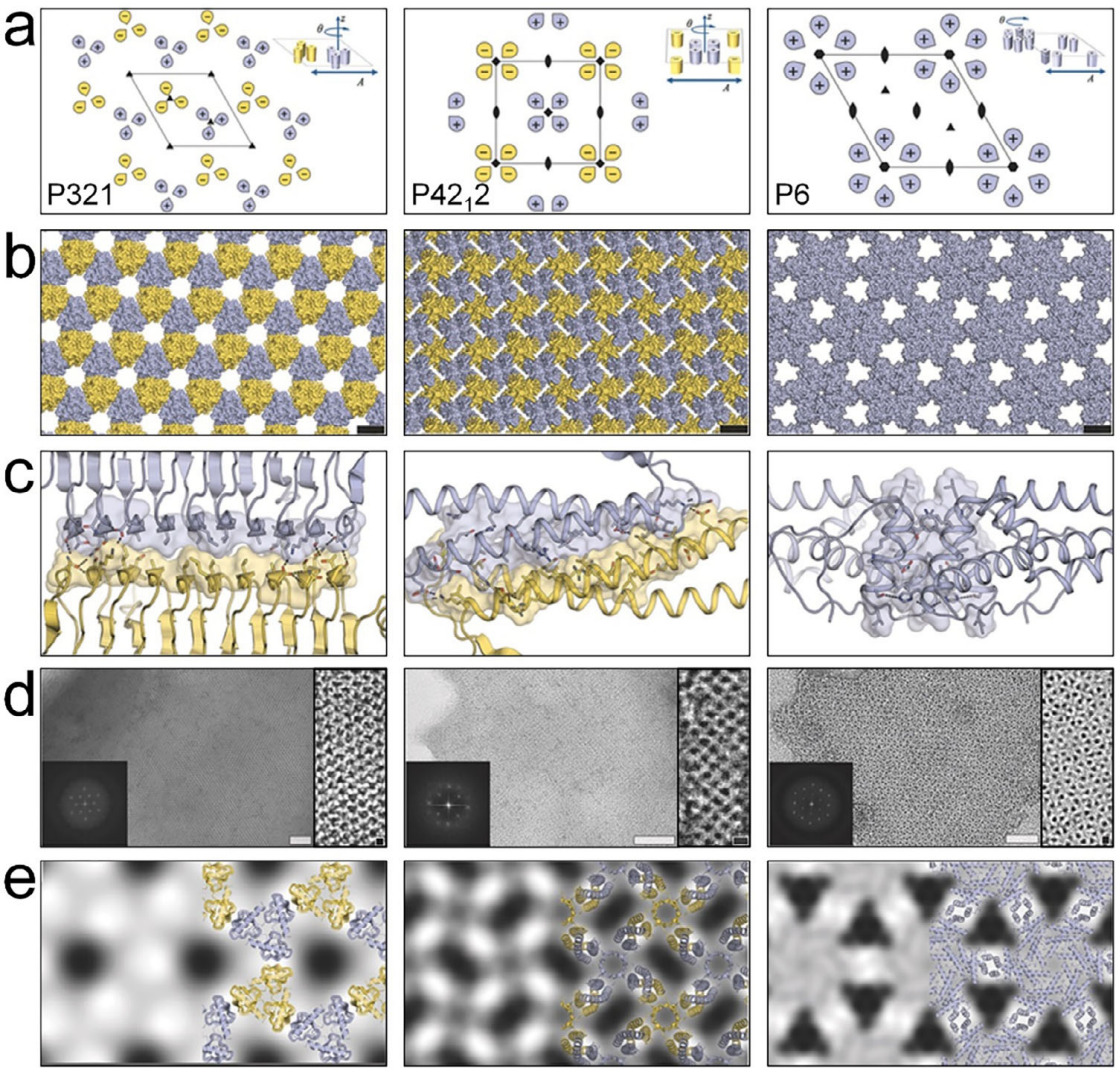
a) Packing configurations of the designed lattices, with the unit cells clearly represented, showcasing the precision in array design. b) Schematic diagrams of the designed 2D protein arrays, highlighting the *P*321, *P*42_1_2, and *P*6 layer group symmetries. c) Detailed designs of the interfaces between protein building blocks, crucial for stabilizing the assembly. d) TEM images of the designed 2D arrays, with insets showing enlarged views and FFT patterns. e) Projection maps calculated from the TEM data. Reproduced with permission.^[^ ^40^ ^]^ Copyright 2015, The American Association for the Advancement of Science.

Computational design strategies have become essential tools in the field of protein assembly, primarily due to their ability to precisely predict and manipulate protein structures and interactions at the molecular level.^[^ ^125^, ^126^, ^127^, ^128^ ^]^ Algorithms and simulations such as Rosetta, Z‐Dock, GRAMM‐X, AlphaFold, and ColabFold enable researchers to predict and optimize the stability, orientation, and interactions of proteins, leading to the formation of well‐defined and functional arrangements.^[^ ^129^, ^130^, ^131^, ^132^ ^]^ These strategies facilitate the systematic incorporation of desired biochemical properties, such as affinity and specificity, into protein assemblies, thereby enhancing the performance of biomaterials.^[^ ^133^, ^134^, ^135^ ^]^ Recent advancements in machine learning algorithms have further demonstrated their effectiveness in modeling complex protein interactions and predicting assembly pathways that were previously unattainable.^[^ ^136^ ^]^ For example, Gonen et al. developed a foundational framework by leveraging noncovalent PPIs to drive the self‐assembly of proteins into well‐ordered 2D protein arrays.^[^ ^40^ ^]^ Using this approach, they systematically arranged protein homo‐oligomers into one of the seventeen 2D layer groups, meticulously sampling the degrees of freedom of the lattice to identify configurations with shape‐complementary interacting surfaces. The interaction energy was subsequently minimized through advanced sequence design calculations. This method successfully produced designs for proteins that self‐assemble into the *P*321, *P*42_1_2, and *P*6 layer groups (**Figure** **16**a–e). The resulting projection maps of micrometer‐scale arrays, both in vitro and in vivo, aligned accurately with the design models, exhibiting the targeted layer group symmetries. Although initial challenges in achieving consistent assembly outcomes due to linker lengths were encountered, this study laid the groundwork for further exploration of protein interface engineering as a method for 2D array formation.

**Figure 17:**
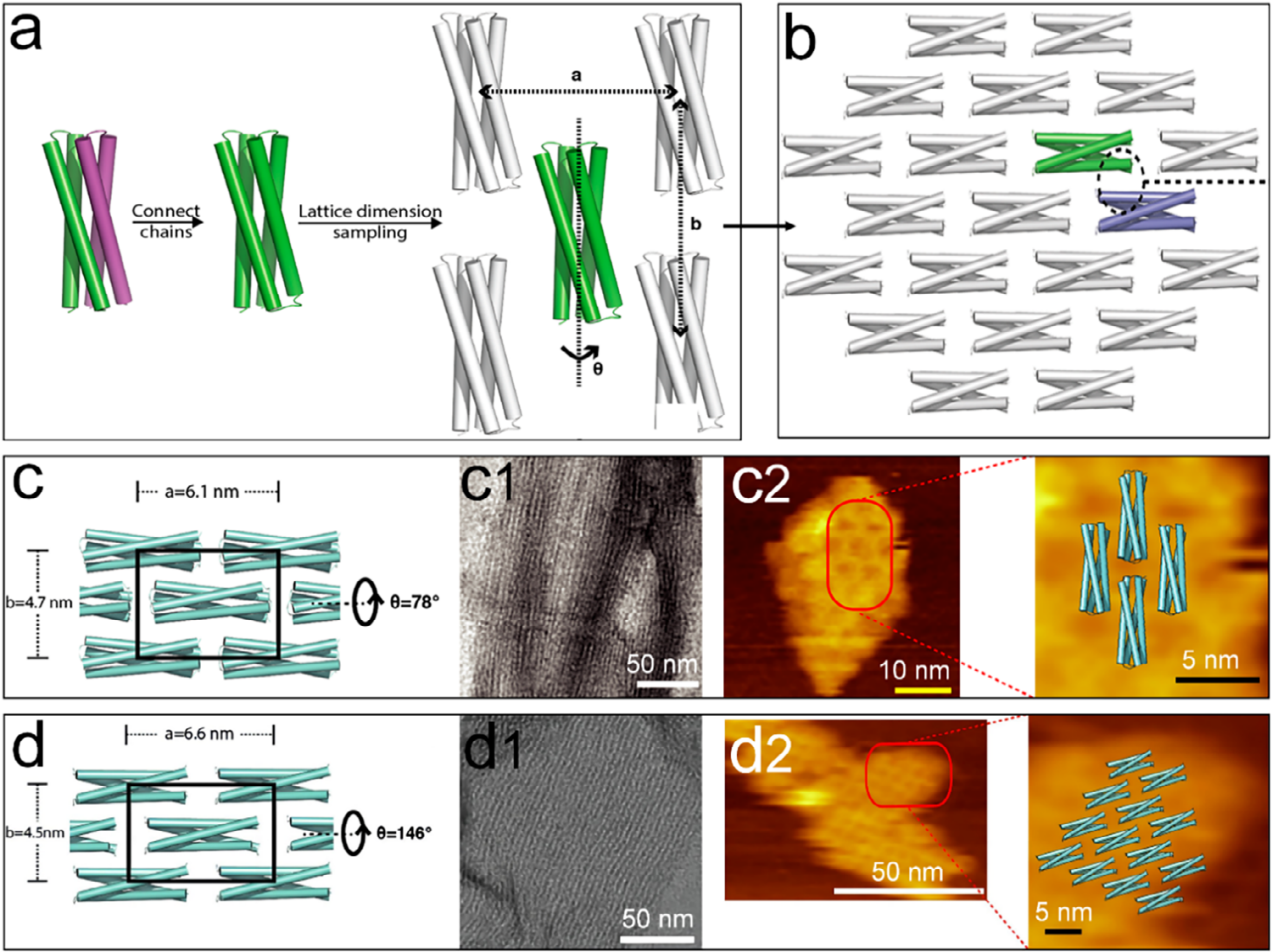
a) A de novo designed homodimer, with the two individual monomers distinguished by green and purple coloration, is engineered as a single continuous chain and subsequently docked within a C12 layer group symmetry characterized by three parameters: a, b, and *θ*. b) The configuration that led to a well‐ordered 2D array. c) The design for 2D protein arrays with exclusive hydrophobic packing (2D‐HP) across the interface, the unit cell was delineated by a black box; c1,c2) TEM and AFM images of 2D‐HP assemblies. d) 2D protein assemblies with a hydrogen bond network (2D‐HBN), the unit cell was demonstrated by a black box; d1,d2) TEM and AFM images of 2D‐HBN assemblies. Reproduced with permission.^[^ ^138^ ^]^ Copyright 2019, American Chemical Society.

Subsequent advancements were made by Matthaei et al., who utilized computational tools to explore the use of fusion multimers and interface mutations in designing 2D arrays.^[^ ^37^ ^]^ S. typhimurium STM4215 protein was rationally selected and designed so that TTM dimer can be formed via a linker in a head‐to‐tail manner. They integrated oligomer fusion with cyclic symmetry and introduced alanine substitutions to mitigate interfacial clashes, facilitating TTM self‐assemble into 2D arrays upon exposure to calcium ions. These 5 nm‐high arrays with *P*3 space group symmetry and a 7.25 nm periodicity can form macroscopic structures exceeding 100 µm in characteristic length. This approach was pivotal in demonstrating the potential of precise mutational design empowered by computations to enhance the assembly properties of proteins. Thomas et al. later conducted a computational redesign of TTM, resulting in calcium‐induced self‐assembly into 2D arrays that could expand to dimensions of dozens of micrometers.^[^ ^137^ ^]^ Chen et al. further advanced the field of 2D protein self‐assembly by pioneering the use of de novo‐designed pseudosymmetric protein building blocks to construct 2D arrays.^[^ ^138^ ^]^ They reconnected a homodimeric helical bundle into a monomeric building block (SC_2L4HC2_23, pseudo‐*C*_2_ symmetry) and subsequently redesigned it using Rosetta (**Figure** **17**a,b). This redesign endowed the building blocks with the necessary pseudosymmetry to enable self‐assembly into 2D‐HP (hydrophobic packing) and 2D‐HBN (hydrogen bond network) arrays characterized by the C12 layer symmetry group (Figure 17c–d2). Two out of ten designed arrays were observed to form micrometer‐scale structures. Notably, this methodology diverges from previous approaches that relied on naturally occurring protein domains, instead leveraging entirely synthetic, pseudosymmetric scaffolds.

**Figure 18:**
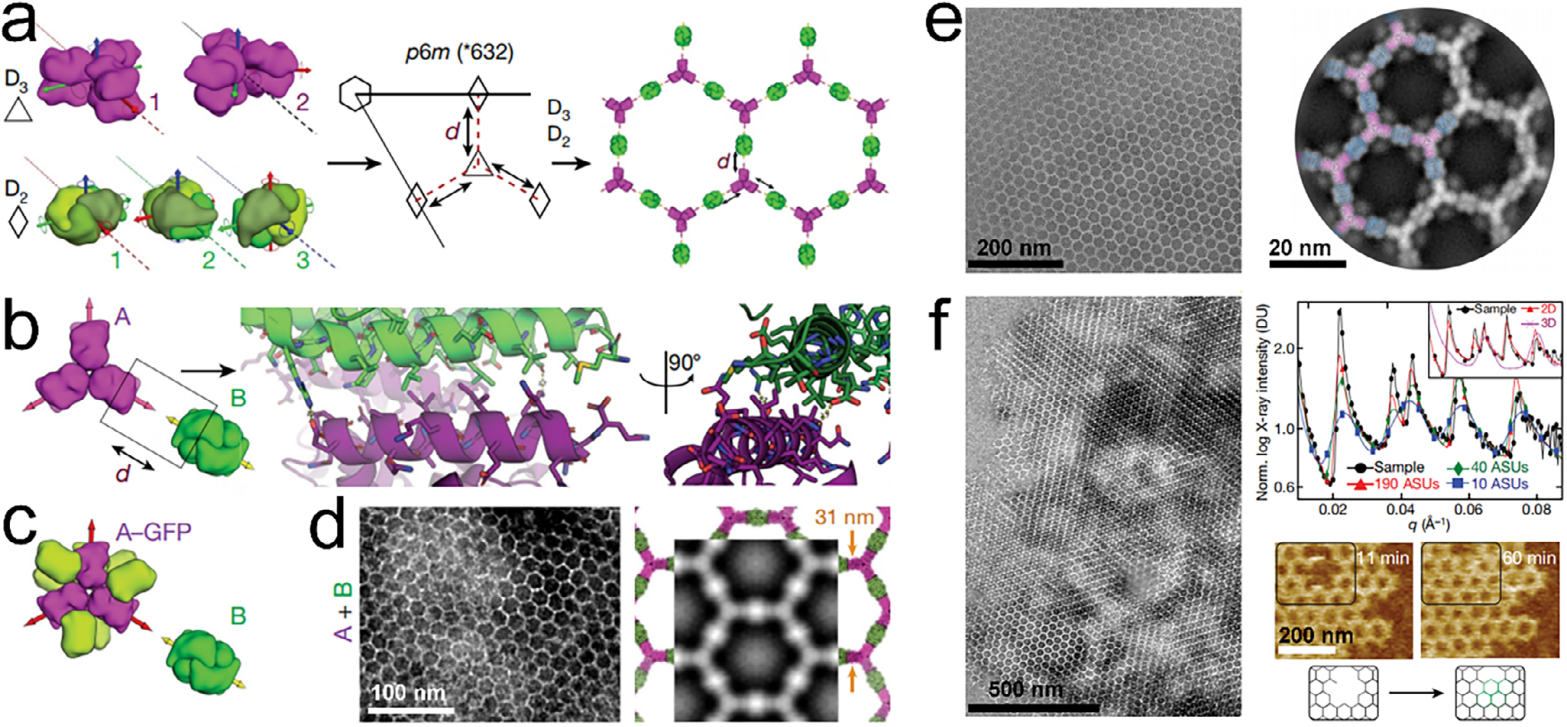
a) Orientation of *D*_3_ and *D*_2_ symmetric protein building blocks to form a heterogeneous *p* *6* *m* protein assembly, showcasing the complexity of mixed‐symmetry designs. b) Interface between the two protein building blocks, essential for maintaining structural coherence. c) Model of A‐GFP. d) TEM images of the *p* *6* *m* arrays, with insets providing detailed views. e) TEM of a monolayer A‐GFP + B array with a computational model overlaid on the averaged density, demonstrating the high degree of structural fidelity. f) Negative‐stain electron microscopy, SAXS profiles, and AFM images of micrometer‐scale arrays, further validating the assembly. Reproduced with permission.^[^ ^139^ ^]^ Copyright 2021, Nature Publishing Group.

Recent advancements in the coassembly of binary protein layers have opened new avenues for the engineering of complex materials with tailored functionalities. Ben‐Sasson et al. computationally designed the rigid interfaces of *D*_3_ and *D*_2_ building blocks, leveraging the inherent advantages of dihedral symmetry to produce 2D arrays with *p* *6* *m* symmetry.^[^ ^139^ ^]^ In this study, to effectively identify *D*_3_ and *D*_2_ homo‐oligomers, they chose dihedral proteins with intrinsic twofold rotation axes as targets to address any possible deviations. Additional degrees of freedom were sampled, and then the two building blocks were aligned with the desired 2D array group, optimizing the spacing and orientation. Using the Rosetta system, the amino acid sequences of the two building blocks were further refined to achieve low‐energy interfaces with a hydrophobic core. Upon completion of these steps, the identified *D*_3_ and *D*_2_ proteins successfully formed 2D arrays with *p* *6* *m* symmetry and micrometer‐scale dimensions, both in vitro and in vivo (**Figure** **18**). These arrays exhibited significant therapeutic potential, particularly when interfaced with cell surfaces, suppressing endocytosis and extending receptor engagement, which could be tunable and relevant for immune evasion. This pioneering work not only establishes a foundation for synthetic cell biology but also highlights the transformative potential of multi‐protein macroscale materials in modulating cellular responses and reshaping both synthetic and living systems.

## 3 The Templated Assembly at Interfaces

Template‐mediated protein assembly plays a crucial role in the self‐assembly process by offering a controllable platform and structural guidance for proteins. First, templates such as DNA,^[^ ^140^, ^141^ ^]^ proteins,^[^ ^31^ ^]^ AuNPs^[^ ^142^, ^143^ ^]^ or solid substrate,^[^ ^53^, ^144^, ^145^, ^146^ ^]^ soft substrate,^[^ ^147^ ^]^ polymer,^[^ ^148^ ^]^ and lipids^[^ ^149^ ^]^ can engage in selective interactions with target proteins through specific binding sites, facilitating highly specific arrangements. This selective binding not only enhances the order of the protein arrays but also allows for precise control over the spatial distribution of proteins at the nanoscale. Second, the physical and chemical properties of the templates can be tailored to promote the arrangement of target proteins under controlled environmental conditions, thereby improving the efficiency and stability of protein self‐assembly. Moreover, the use of templates enables multifunctional integration; for example, DNA‐mediated assembly can integrate various proteins to achieve more complex functionalities.^[^ ^150^ ^]^ This approach not only provides a robust strategy for constructing 2D protein arrays but also opens up new possibilities for applications in biomaterial design and biosensing technology. In the following sections, research progresses are grouped based on the specific type of templates used for driving 2D protein self‐assembly, ranging from hard to soft materials, even at liquid‐liquid interfaces.

### 3.1 Hard Template

#### 3.1.1 Mica

**Figure 19:**
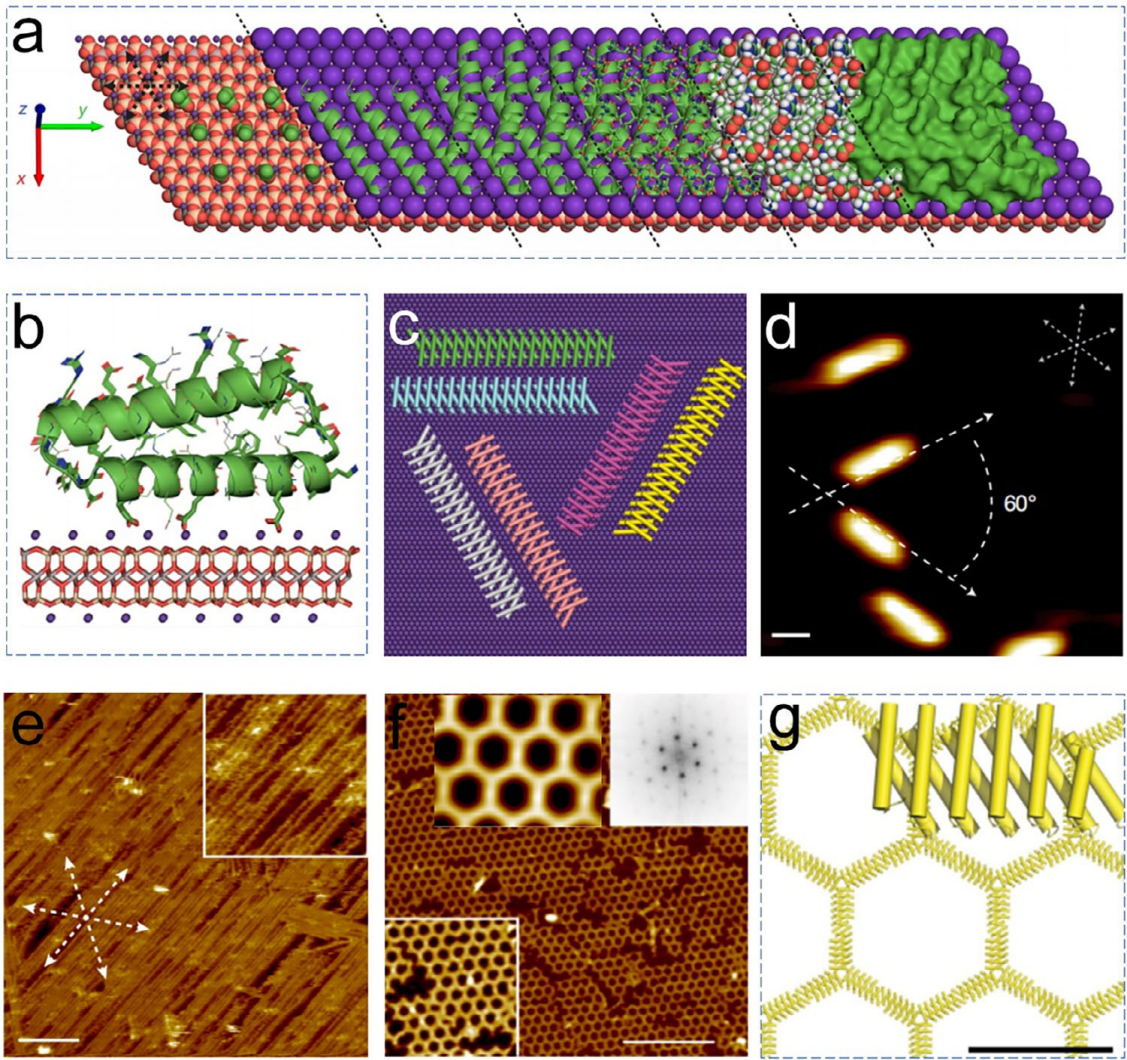
a) Model depicting the lattice‐matching of DHR10‐mica18 protein to mica (001) surface through the K^+^ sublattice. A single 18‐repeat DHR10‐mica18 molecule is visualized from a side perspective and a top‐down view. b) Schematic illustration of the DHR10‐mica18 protein building blocks binding to mica surface via K^+^ sublattice. c) Predicted models of the protein‐mica interface and the orientation of DHR10‐mica18 on a K^+^‐rich mica surface. d) AFM images of the three main orientations of DHR10‐mica18. Assemblies of various variants on mica: e) DHR‐mica6‐NC, and f,g) DHR‐mica6‐H. Reproduced with permission.^[^ ^152^ ^]^ Copyright 2019, Nature Publishing Group.

Solid substrate‐mediated 2D protein assembly has emerged as a critical strategy for constructing protein arrays, thanks to its ability to harness the unique properties of interfaces, such as hydrophilicity, hydrophobicity, and charge.^[^ ^151^ ^]^ The primary advantage of these properties in interfacial‐mediated assembly is their ability to promote specific binding orientations and controlled packing densities of proteins. This approach also enables the creation of dynamic and responsive systems, where external stimuli can modulate protein binding and release, and allows for the integration of multiple protein types within a single array, thereby mimicking complex biological interactions.^[^ ^152^, ^153^ ^]^ For example, Pyles et al. used muscovite mica (001) as the substrate and de novo designed helical repeat protein DHR10 as the assembly protein building blocks.^[^ ^152^ ^]^ The mica surface is characterized by an atomically flat mineral substrate with a pseudohexagonal tessellation of charged cavities that bind to K^+^ ions (**Figure** **19**a). Importantly, the charge state of the substrate can be controlled by adjusting the cation concentration. Leveraging this feature, they designed an inorganic mineral template to guide the assembly of DHR10 (Figure 19b,c). To enhance protein binding to the mineral template, Glu or Ala residues in DHR10 were redesigned to align with the pattern of the K^+^ ion‐containing cavities. By modifying the length of the DHR10 and adjusting the salt concentration to control protein mobility on the template, the researchers could precisely regulate the orientational order and patterning of proteins on the surface (Figure 19d). For instance, at high K^+^ concentrations, 1D wires and well‐ordered 2D protein arrays were formed on the mica sublattice, with structural scales exceeding the millimeter level (Figure 19e–g).

**Figure 20:**
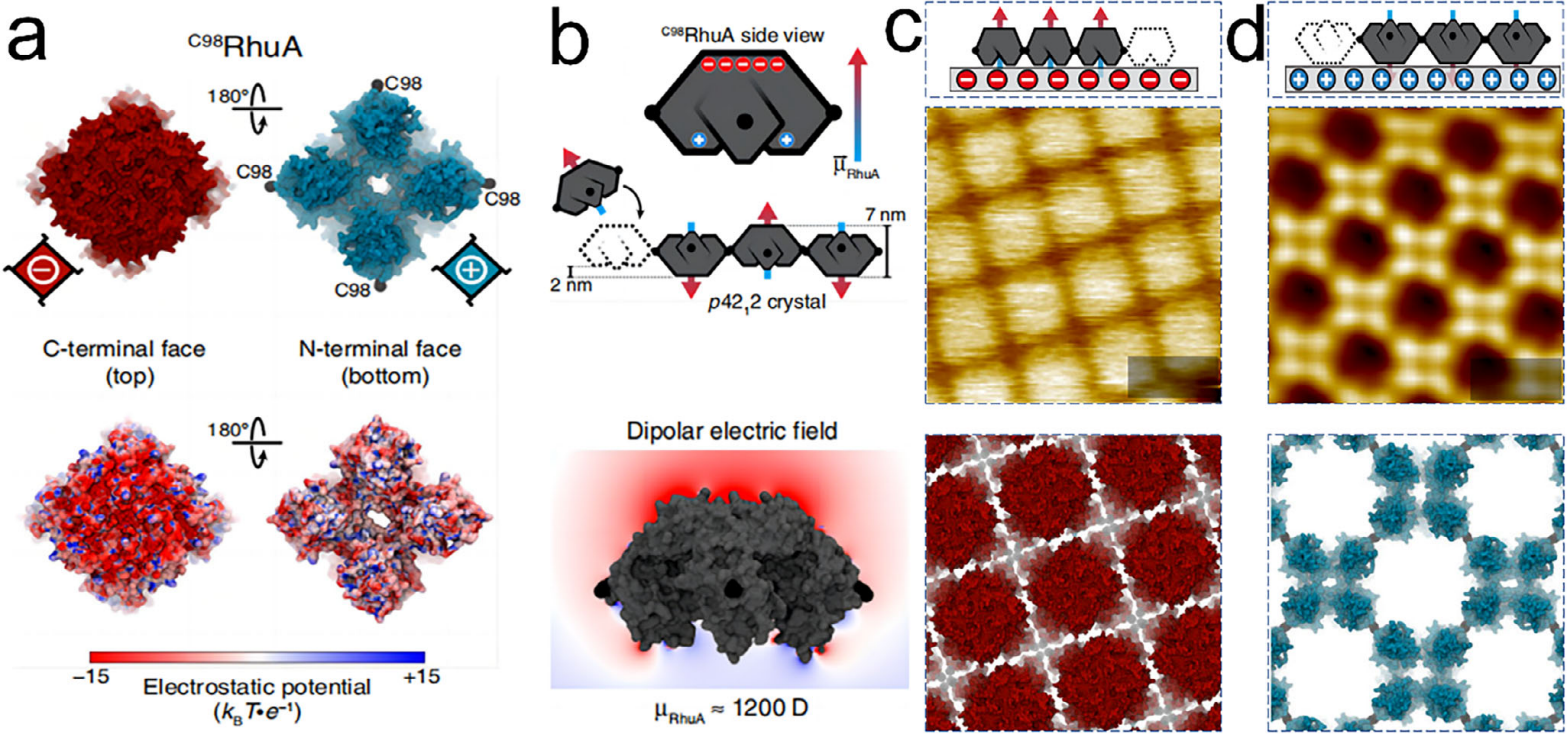
a) Schematic representation and electrostatic potential maps of the ^C98^RhuA C‐, N‐termini surfaces. b) The macrodipole moment and global electric field of ^C98^RhuA. c,d) Structural models of mica‐templated 2D ^C98^RhuA crystals, with AFM images. Reproduced with permission.^[^ ^154^ ^]^ Copyright 2020, Nature Publishing Group.

Employing the same muscovite mica substrate, Zhang and colleagues investigated the assembly behavior of the ^C98^RhuA protein.^[^ ^154^ ^]^ RhuA is characterized by negatively charged residues in its C‐terminal domains and positively charged residues in its N‐terminal domains (**Figure** **20**a), resulting in a significant dipole moment for the protein (Figure 20b). Drawing inspiration from RhuA charge distribution, they manipulated the K^+^ concentration on the mica surface to control the surface‐templated assembly of ^C98^RhuA mutants. At low K^+^ concentrations, only the positively charged surfaces of the ^C98^RhuA adhered to the mica surface, forming 2D arrays with *P*4 symmetry (Figure 20c). Conversely, at high K^+^ concentrations, the negatively charged surface interacted with the muscovite mica, resulting in well‐ordered 2D monolayer *P*4 arrays (Figure 20d). Furthermore, at elevated protein monomer concentrations and 3 m K^+^, the salting‐out effect led to the formation of protein bilayer lattices. The study concluded that the ability to finely tune multiscale interactions provides a robust framework for the rational design of protein assemblies, with profound implications for the development of functional materials and devices.

#### 3.1.2 Gold

**Figure 21:**
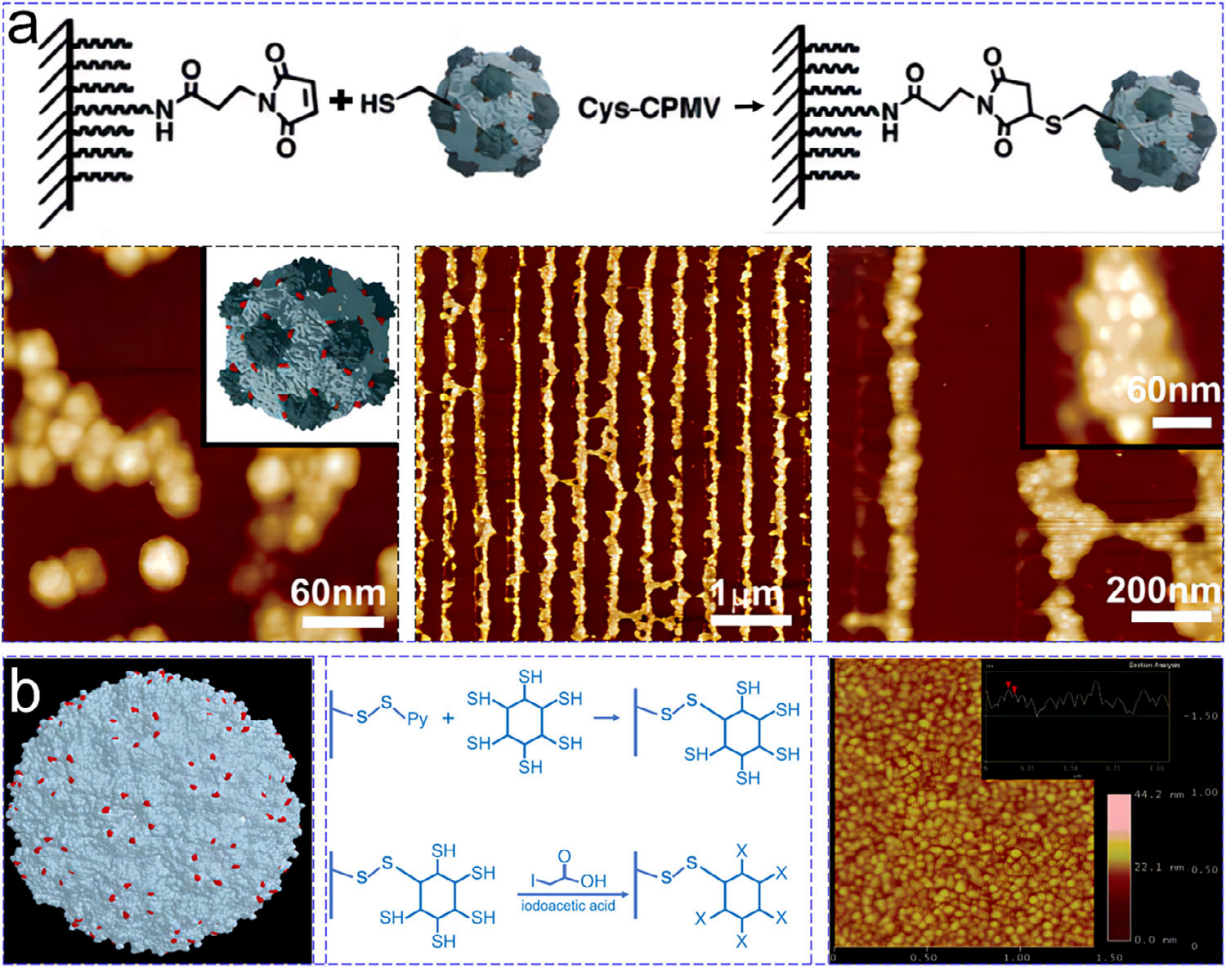
a) Schematic illustration of gold templated 2D assembly of Cys‐modified cowpea mosaic virus (CPMV), with AFM images of 2D assemblies. Reproduced with permission.^[^ ^155^ ^]^ Copyright 2003, American Chemical Society. b) Space‐filling model of cowpea chlorotic mottle virus (CCMV) A163C, highlighting the engineered Cys residues in red, and the passivation of unbound cysteine residues with iodoacetic acid, along with corresponding AFM images. Reproduced with permission.^[^ ^156^ ^]^ Copyright 2003, American Chemical Society.

Gold substrates were employed as templates to steer the assembly of cowpea mosaic virus (CPMV) and cowpea chlorotic mottle virus (CCMV) protein cages. Cheung et al. successfully introduced Cys residues on the exterior surface of CPMV, which bound to the gold substrate surfaces, resulting in the formation of arrays composed of parallel lines, each with a width of 30 nm (**Figure** **21**a).^[^ ^155^ ^]^ Inspired by this approach, Klem et al. genetically modified the CCMV (A163C) protein cage, substituting an alanine residue at position 163 with Cys, allowing the viral cages to attach to gold substrates and form 2D disordered monolayer (Figure 21b).^[^ ^156^ ^]^ Additionally, Masuda et al. demonstrated that 2D arrays of ferritin could be replicated onto metal films using ion‐beam sputtering.^[^ ^157^ ^]^ Other substrates, such as carbonaceous materials, have also been employed as templates for the assembly of a 2D hexagonally close‐packed array.^[^ ^158^ ^]^ These techniques underscore the potential of solid substrates for creating functional nanostructures with enhanced stability and versatility.

### 3.2 Soft Template

#### 3.2.1 DNA

**Figure 22:**
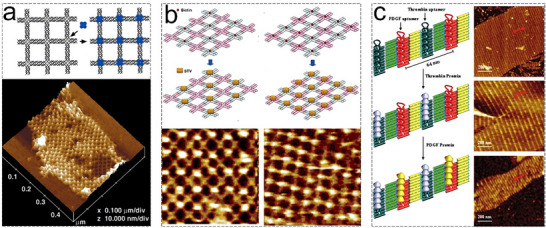
a) DNA nanogrids templated the assembly of streptavidin into protein nanoarrays on DNA lattices. Reproduced with permission.^[^ ^44^ ^]^ Copyright 2003, The American Association for the Advancement of Science. b) Self‐assembly of AB tiles and subsequent binding of streptavidin to A*B and A*B* arrays, shown in AFM images with a scan size of 150 nm × 150 nm. Reproduced with permission.^[^ ^159^ ^]^ Copyright 2005, American Chemical Society. c) Periodic 2D multiprotein nanoarrays based on 2D DNA nanoarrays. Reproduced with permission.^[^ ^161^ ^]^ Copyright 2007, American Chemical Society.

DNA‐based self‐assembly techniques have become fundamental in the creation of complex 2D protein arrays, leveraging the unique programmability of nucleic acids. Cross‐shaped DNA tiles, formed by the self‐assembly of 4 × 4 tiles, can create 2D DNA nanogrids with periodic cavities, making them excellent scaffolds for organizing protein molecules. For instance, Yan and colleagues constructed a 2D DNA nanogrid that served as a template for assembling streptavidin proteins into regular 2D arrays.^[^ ^44^ ^]^ They created a series of 4 × 4 tiles, each with a biotin group at the center, which self‐assembled into 2D nanogrids featuring periodic square biotin groups. Subsequently, the periodic arrangement of streptavidin proteins was achieved by templated self‐assembly onto these DNA tiles. (**Figure** **22**a). However, the challenge lies in programming the self‐assembly of DNA‐templated protein arrays with precise spatial distances and higher pattern complexity. To address this, their team developed two 4 × 4 tiles that self‐assembled into periodic two types of 2D DNA nanogrids.^[^ ^159^ ^]^

Using streptavidin as protein units, these 2D DNA nanogrids showcased precise control over the spacing between individual streptavidin protein molecules through specific interactions between streptavidin and biotin. Two types of 2D streptavidin arrays were obtained (Figure 22b), employing A and B tiles as selectable binding sites that alternately associate through rationally designed sticky ends, self‐assembling into 2D nanogrids with controlled periodicity. Another general approach for templating macromolecules onto DNA tile arrays involves using DNA‐binding proteins that exhibit either sequence‐specific or structure‐specific binding properties. Turberfield and colleagues demonstrated the organization of a bacterial recombination protein, RuvA, into 2D arrays templated by 2D DNA arrays.^[^ ^160^ ^]^ While promising, achieving high‐density and more complex 2D protein nanoarrays remains a significant challenge. Yan and colleagues further assembled 2D nanoarrays of multiple proteins templated by self‐assembled DNA scaffolds, enabling deterministic positioning of proteins on fully addressable DNA nanoarchitecture (Figure 22c).^[^ ^161^ ^]^ Rinker and colleagues expanded on this concept by exploring how self‐assembled DNA nanostructures could be designed for distance‐dependent multivalent ligand–protein binding.^[^ ^162^ ^]^ Moreover, He and co‐workers developed a 2D DNA array for periodic antibody nanoarrays.^[^ ^163^ ^]^ The center of cross‐shape DNA tiles was grafted onto two fluorescein antigen molecules and subsequently self‐assembled into 2D arrays, thereby resulting in the formation of 2D tetragonal antibody arrays.

**Figure 23:**
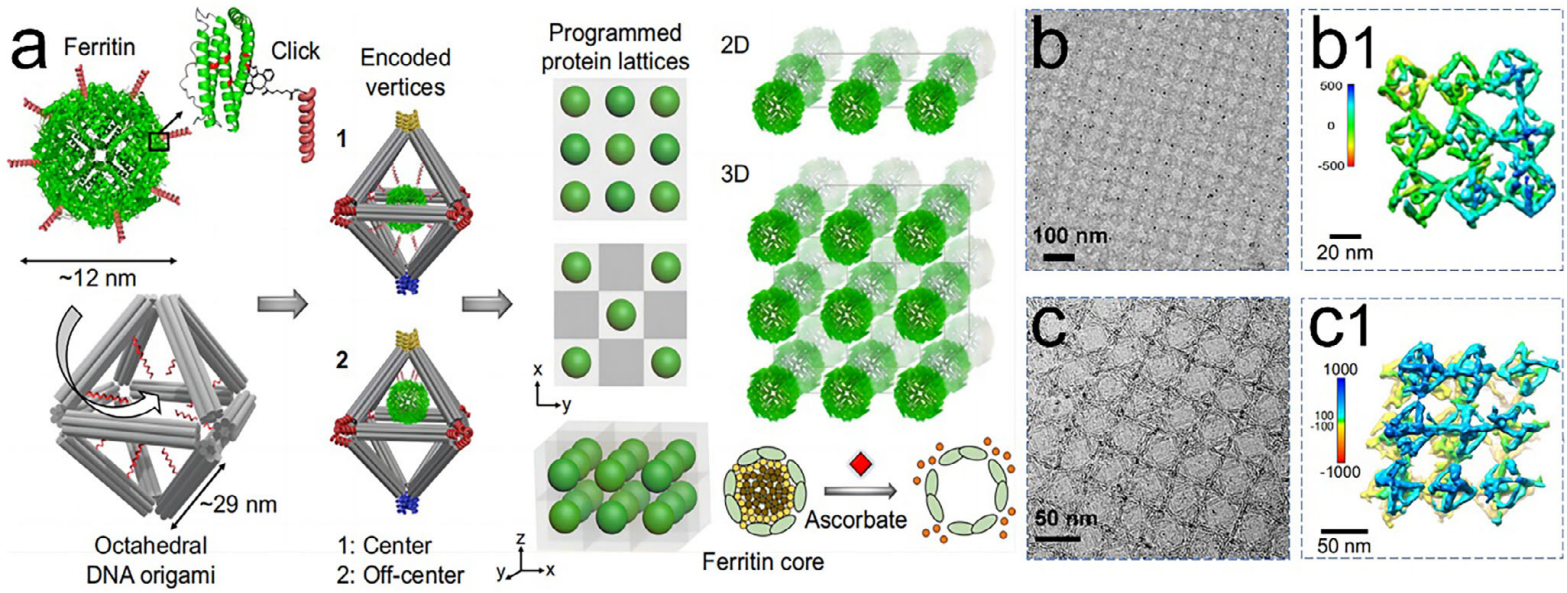
a) Structure of ferritin, and DNA octahedra for integrating ferritin to form protein voxels, together with the schematic illustration of 2D and 3D ferritin crystals. b) TEM image and b1) 3D density maps of single‐layer ferritin/octahedra arrays. c) Cryo‐EM image and c1) 3D density map of a double‐layer array. Reproduced with permission.^[^ ^164^ ^]^ Copyright 2021, Nature Publishing Group.

More recently, Wang et al. introduced a novel approach for the programmable assembly of bioactive 2D and 3D protein arrays by integrating ferritin with DNA frames to create protein voxels.^[^ ^164^ ^]^ These DNA frameworks allow for the controlled placement and encapsulation of guest proteins within each voxel. Ferritins and apoferritins were covalently modified with single‐stranded (ss) DNA to enable efficient encapsulation (≈70%) inside 3D wireframe octahedral origami structures through hybridization with complementary ssDNA at specific sites (**Figure** **23**a). This high‐yield and stable encapsulation was achieved by stepwise surface modification of the proteins, ensuring their targeted placement at designated locations within the framework. The encapsulation positions of ferritin within the frames can be precisely controlled, allowing placement at the center or closer to a vertex. The designed 2D single‐ and double‐layered, ferritin and apoferritin lattices were formed by establishing predefined vertex‐to‐vertex Watson–Crick base pair connections (Figure 23b–c1). These ordered 3D ferritin arrays can be converted into coreless apoferritin arrays while maintaining structural integrity, using ascorbate to reduce and release iron ions from ferritin. The open architecture of the individual frames and assembled frameworks provides a native‐like environment and ensures molecular accessibility for the encapsulated proteins. Notably, the structural stability and biological activity of ferritin, including its ability to release iron ions, are preserved within the assembled arrays.

#### 3.2.2 Protein

**Figure 24:**
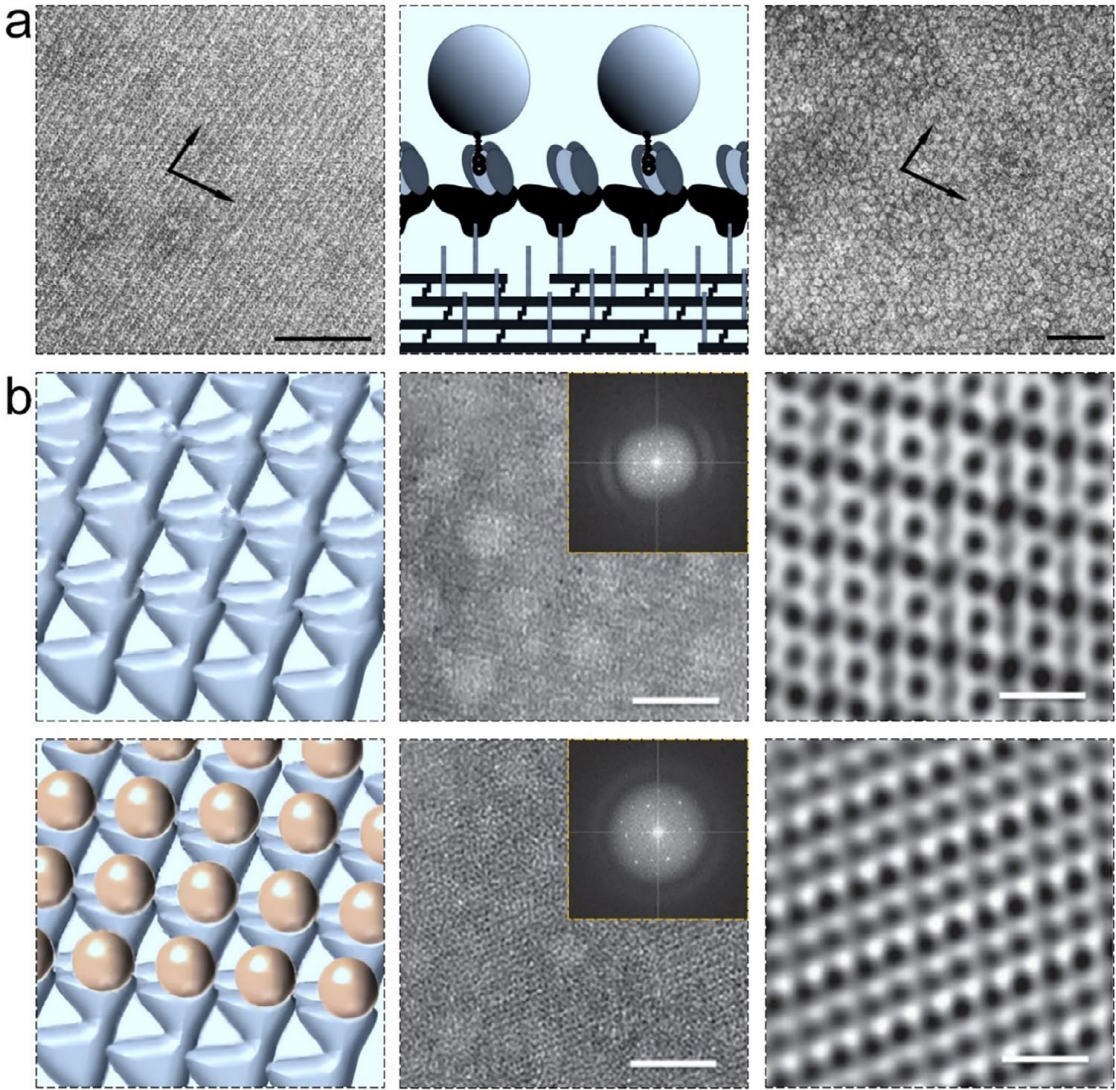
a) 2D S‐layer arrays direct the assembly of biotinylated ferritin into 2D arrays on cell‐wall fragments, with the TEM images showing the 2D ferritin arrays. Reproduced with permission.^[^ ^31^ ^]^ Copyright 2002, National Academy of Sciences. b) 2D S‐layer arrays and templated self‐assembly of methyl parathion hydrolase (MPH) into 2D MPH arrays, together with the TEM images and real map of 2D S‐layer arrays and 2D MPH arrays. Reproduced with permission.^[^ ^166^ ^]^ Copyright 2015, Wiley‐VCH Verlag GmbH and Co. KGaA, Weinheim.

Protein assemblies provide a versatile range of templates for organizing heterologous proteins into multidimensional arrays.^[^ ^165^ ^]^ Notably, S‐layer proteins have a unique ability to self‐assemble into 2D crystalline arrays, making them ideal for high‐density display of large protein fragments.^[^ ^22^ ^]^ Sara and colleagues demonstrated the self‐assembly of S‐layer‐streptavidin fusion proteins into highly ordered and oriented 2D protein arrays.^[^ ^31^ ^]^ These arrays effectively presented the streptavidin on the outer surface, serving as a functional template to pattern biotinylated ferritin into 2D arrays through biotin–streptavidin interactions (**Figure** **24**a).

The exceptional ability of 2D S‐layer proteins to allow periodic arrangement of other biotinylated proteins underscores their versatility. Wang et al. further explored the potential of the S‐layer protein EA1. They genetically fused EA1 with methyl parathion hydrolase (MPH) and successfully self‐assembled the fusion proteins into 2D EA1‐MPH arrays (Figure 24b).^[^ ^166^ ^]^ These dual‐functionalized 2D protein assemblies exhibited both MPH activity and the ability to bind anthrax‐specific antibodies. This dual functionality greatly enhanced enzymatic stability and significantly improved the sensitivity of anthrax detection compared to unassembled complexes. These findings highlight the potential and versatility of S‐layer templates in various applications.

#### 3.2.3 Polymers

**Figure 25:**
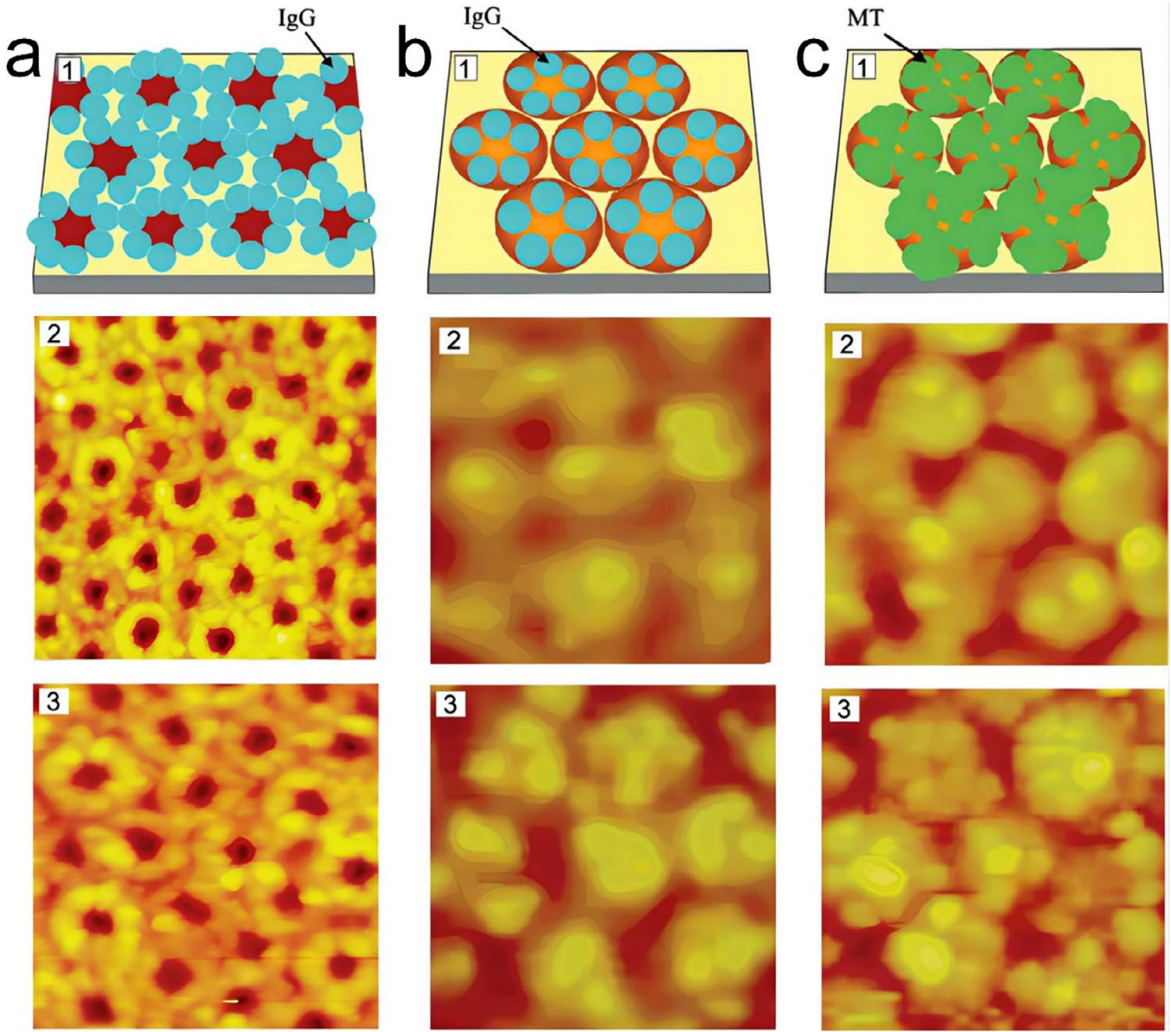
Schematic illustrations and AFM images of the assembly of IgG and mushroom tyrosinase (MT) molecules on PS‐b‐PVP templates. a) IgG molecules absorbed on open PS‐b‐PVP templates. b) IgG molecules assembled on reverted PS‐b‐PVP templates c) MT molecules assembled on reverted PS‐b‐PVP templates. AFM images are 2) 300 × 300 nm and 3) 180 × 180 nm in size. Reproduced with permission.^[^ ^148^ ^]^ Copyright 2007, American Chemical Society.

Amphiphilic diblock copolymers have emerged as a powerful template for protein organization due to their tunable morphologies and precise control over spatial periodicity in protein arrangements. A groundbreaking investigation by Kumar and colleagues established the foundational principles of this approach, demonstrating that nanopatterned films of polystyrene‐block‐poly(methylmethacrylate) facilitated 20S proteasomes adsorbed to the polystyrene domains into 2D disordered protein structures.^[^ ^167^ ^]^ Based on this finding, this strategy has been extended to polystyrene‐block‐poly(4‐vinylpyridine) (PS‐b‐PVP) diblock copolymer. PS‐b‐PVP could self‐assemble into 2D micelles with the structural variety and chemical heterogeneity on silicon substrates.^[^ ^148^ ^]^ Then, the spontaneous assembly of proteins guided by these polymeric templates results in the formation of high‐density 2D protein arrays (**Figure** **25**a–c). These arrays are distinguished by their nanoscopic repeat spacings, which are crucial for a variety of biological and biophysical applications. More importantly, by carefully adjusting the dimensions of the underlying block copolymer templates, the repeat spacing of 2D protein nanoarrays can be finely tuned. Moreover, the self‐assembled proteins within these arrays retain their native conformations and biological activities over an extended period. This stability is of utmost importance as it ensures the reliability and reproducibility of subsequent biological assays. Subsequently, diblock copolymers such as poly(styrene‐block‐isoprene) and poly(butadiene)‐block‐ poly(ethylene oxide) systems have been employed as effective templates for assembling protein into 2D films.^[^ ^168^, ^169^ ^]^

Besides diblock copolymers, liquid–polymer interfaces have emerged as a focal point of extensive research.^[^ ^170^, ^171^ ^]^ Keller et al. conducted a notable study on ultrahigh molecular weight polyethylene (UHWMPE) to direct the assembly of protein molecules. They found that the ordered crystalline lamellae of UHWMPE promote a preferential alignment of single human plasma fibrinogen.^[^ ^172^ ^]^ Specifically, the long axes of these proteins tend to align parallel to the major axis of the lamellae. Once the surface reaches nearly monolayer coverage, fibrinogen molecules formed dense 2D films. This film exhibits a distinct fingerprint‐like pattern that mirrors the underlying nanostructure of the UHMWPE surface. The mechanism behind the formation of 2D films was further explored by obtaining a single‐molecule tracking map of fibrinogen dynamics on nanostructured high‐density polyethylene (HDPE) surfaces.^[^ ^173^ ^]^ The surface occupancy maps generated from this approach enabled the identification of discrete patches of a dense 2D protein layer. Moreover, it was observed that the molecules incorporated into this layer had a long residence time. The study found that the slow anisotropic diffusion of individual fibrinogen proteins within this ordered layer moves significantly faster along the drawing or polymer chain direction of the HDPE films. This phenomenon was attributed to the confinement of individual proteins within a single row of crystalline lamellae. The ability of nanostructured polymer films to template ordered 2D fibrinogen layers holds great significance in the field of biomaterials.

#### 3.2.4 Lipid

The lipid monolayer interface also is a powerful approach for regulating 2D protein assembly.^[^ ^174^, ^175^ ^]^ For example, the Okahata team pioneered an innovative strategy to fabricate 2D GOx films via the precomplexation of hydrophilic proteins with cationic lipids.^[^ ^176^, ^177^ ^]^ Transferred films maintain enzymatic activity, demonstrating the efficacy of this organic solvent‐based processing pathway. Parallel advancements have emerged in the field of S‐layer protein organization. The negatively charged inner surface of S‐layer subunits facilitates their attachment to various lipid layers, enabling the creation of cross‐linkable matrices for functional molecule immobilization.^[^ ^178^ ^]^ Building on the properties of the S‐layer, the Chung group has provided insight into the molecular mechanisms of S‐layer array formation on lipid bilayers.^[^ ^149^ ^]^ Using in situ AFM, they elucidated a multi‐stage crystallization process: initial adsorption of extended monomers, formation of amorphous clusters, phase transition to crystalline clusters composed of compact tetramers, and self‐catalytic growth at crystal edges into 2D S‐layer arrays. These advancements in protein assembly at lipid interfaces have a potential role in nanotechnology and biotechnology.

### 3.3 Liquid–Liquid Interfaces

The liquid–liquid interface has evolved into an effective template to assemble protein into 2D arrays for governing cellular responses. For example, the denaturation of globular proteins drives the spontaneous formation of adaptive 2D nanosheets for cell spreading and proliferation.^[^ ^179^ ^]^ Building on this foundation, Jia et al. systematically demonstrated that the denaturation of serum protein and then interfacial self‐assembly into 2D monolayer nanosheets at the liquid–liquid interfaces, creating mechanically tunable substrates capable of directing human mesenchymal stem cell (hMSC) morphogenesis.^[^ ^180^, ^181^ ^]^ Subsequently, 2D network of protein nanofibrils was also assembled at a liquid–liquid interface and used as an adaptive biomaterial for neuronal differentiation of hMSC through a signaling mechanism involving focal adhesion kinase.^[^ ^182^ ^]^

**Figure 26:**
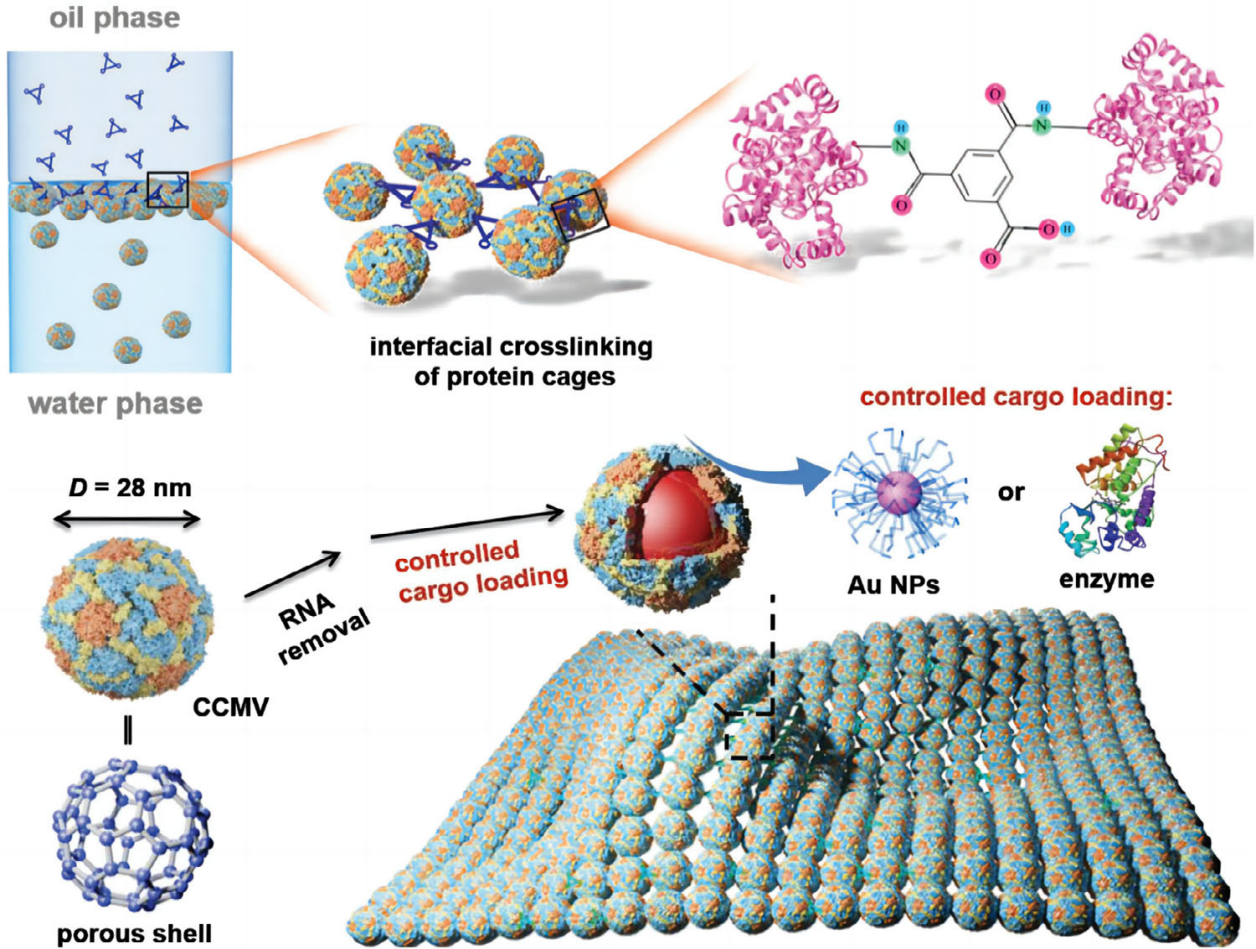
Fabrication of 2D CCMV arrays with customized functionality by interfacial crosslinking. Reproduced with permission.^[^ ^147^ ^]^ Copyright 2018, Wiley‐VCH Verlag GmbH and Co. KGaA, Weinheim.

Parallel developments demonstrate the versatility of liquid–liquid interfaces for engineering functional protein assemblies. Protein cage, CCMV capsids, have been precisely organized into 2D functional films through interfacial crosslinking strategies. Liu et al. engineered tunable CCMV monolayers using trimesoyl chloride‐mediated covalent assembly at oil–water boundaries, preserving the viral particles innate cargo encapsulation capacity (**Figure** **26**).^[^ ^147^ ^]^ This platform enabled the co‐incorporation of AuNPs and HRPs, creating hybrid systems that maintain enzymatic activity while gaining inorganic functionality. Therefore, these interfacial engineering approaches lay a fundamentally novel foundation for investigating the cross‐talk within the cell microenvironment. Meanwhile, these effective means also mirrored the importance of 2D protein arrays in regulating cell behavior.

## 4 2D Protein Arrays as Scaffold

Self‐assembled protein structures can serve as efficient scaffolds for incorporating diverse functional elements, such as metal particles,^[^ ^183^ ^]^ inorganic ions,^[^ ^184^, ^185^, ^186^ ^]^ biomolecules,^[^ ^187^, ^188^ ^]^ and quantum dots (QDs),^[^ ^69^ ^]^ due to their unique structural features and functionalization capabilities, which hold significant potential for various applications.^[^ ^92^ ^]^ Notably, these assemblies, especially 2D protein arrays, offer highly controllable surfaces that allow for precise positioning and arrangement of functional elements through specific interactions.^[^ ^20^ ^]^ The spatial organization is crucial as it directly impacts the functional properties and reactivity of the resulting materials. For example, the versatility and tunability of protein arrays facilitate interactions with various functional components, resulting in composite materials with distinct capabilities. Surface modifications of proteins can further optimize these functional parameters, customizing the materials for specific applications. Furthermore, the self‐assembly properties of proteins facilitate efficient integration with biomolecules, thereby improving the performance of biosensors and biocatalysts. This templated assembly strategy not only enhances the efficiency and stability of the assembled functional elements but also opens up new possibilities for constructing functionally diverse nanostructures. In the following sections, research progresses are described based on the types of nanomaterials that are positioned on the 2D protein arrays.

### 4.1 AuNPs

**Figure 27:**
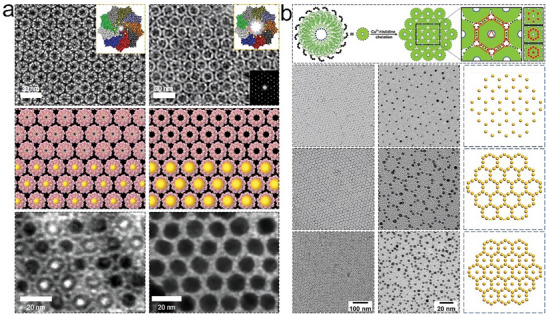
a) 2D AuNP arrays templated by 2D chaperonin crystals. Reproduced with permission.^[^ ^189^ ^]^ Copyright 2002, Nature Publishing Group. b) 2D T103C‐TMV‐4His arrays direct the formation of highly ordered 2D AuNP arrays, demonstrating the precision and control achieved through protein templating. Reproduced with permission.^[^ ^92^ ^]^ Copyright 2019, Wiley‐VCH Verlag GmbH and Co. KGaA, Weinheim.

2D protein arrays, distinguished by their high orderliness and precise geometrical control, serve as efficient templates for guiding the spatial arrangement of AuNPs. This templating capability facilitates the fabrication of highly controllable nanostructures, which are essential for optimizing functional properties and reactivity. In an early foundational study, McMillan et al. introduced a method for creating ordered AuNP arrays using engineered 2D heat shock protein 60 (HSP60) templates.^[^ ^189^ ^]^ They pioneered the use of crystalline 2D protein assemblies as templates for generating ordered NP arrays. Nine subunits of HSP60 self‐assembled into hollow octadecameric double‐ring structures with apical pores ranging from 3 to 9 nm in diameter. By introducing Cys residues in specific positions on the surface of HSP60 monomers, hexagonally packed 2D arrays exhibited periodic thiol groups for anchoring AuNPs, organizing them into 2D AuNP arrays (**Figure** **27**a). The size selectivity of AuNPs was attributed to the precise design of the functional thiol groups. Building on these advancements, Thomas et al. developed self‐assembling 2D protein arrays that served as a versatile platform for constructing AuNP nanostructures.^[^ ^137^ ^]^ They redesigned proteins via a computational design strategy named TTM that forms hexagonal 2D arrays upon Ca^2+^‐trigger. By incorporating a gold‐binding peptide on TTM monomers, they achieved a precise assembly of AuNPs on the protein array surface.

**Figure 28:**
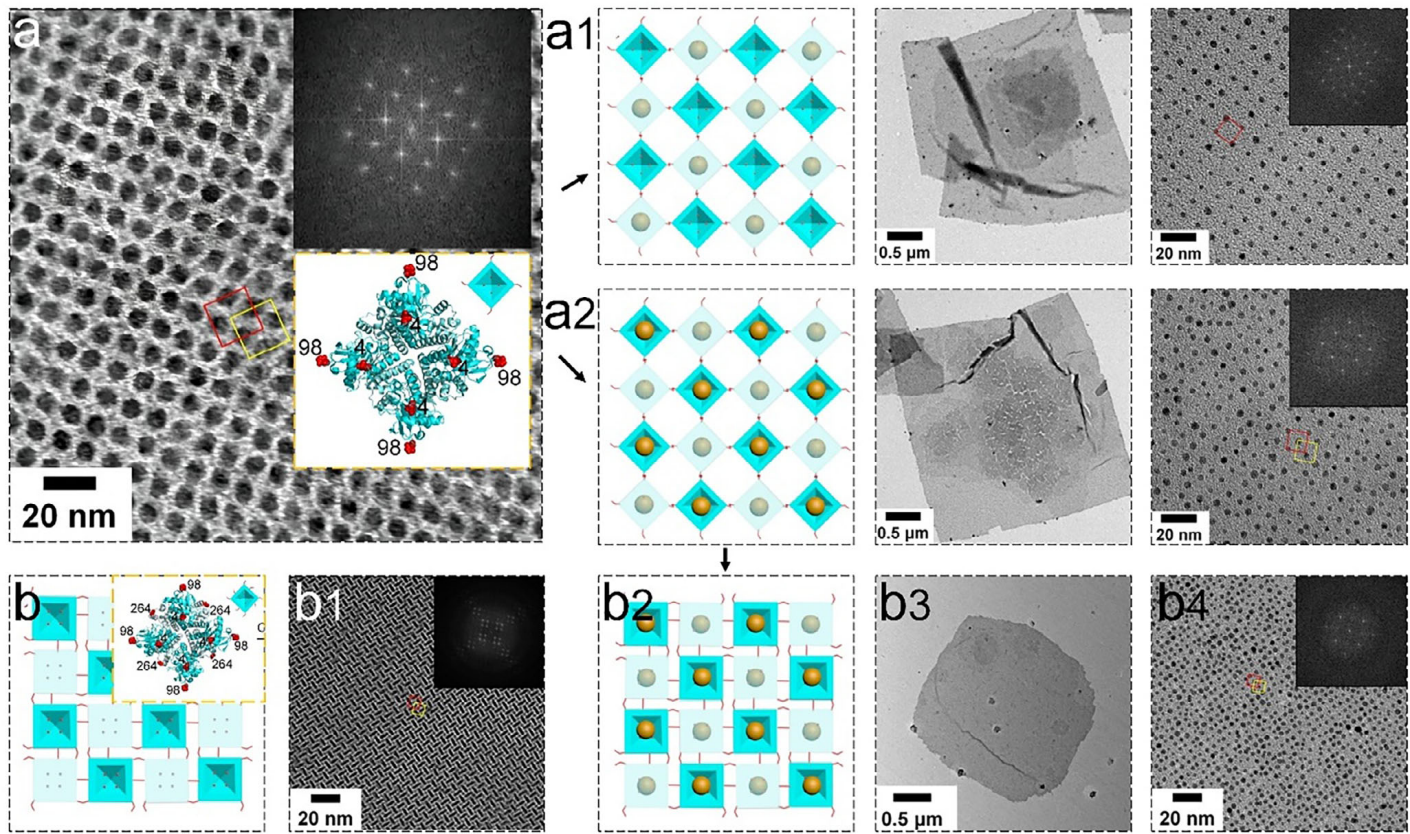
a) TEM characterization of 2D ^C4/C98^RhuA protein arrays. a1,a2) The self‐assembly of 2D monolayer and bilayer AuNP arrays templated by 2D ^C4/C98^RhuA protein crystals. b–b4) Design of ^C4/C98/C264^RhuA tetramers and their self‐assembly into 2D arrays, resulting in 2D bilayer AuNP arrays with smaller unit cells. Reproduced with permission.^[^ ^19^ ^]^ Copyright 2019, American Chemical Society.

Further progress in this field was achieved by our group. Zhang et al. demonstrated the precise self‐assembly of AuNPs into ordered 2D TMV templates.^[^ ^92^ ^]^ T103C‐TMV‐4His were constructed and self‐assembled into 2D TMV monolayer sheets induced by Cu^2+^‐His interactions, which then acted as functional templates for the formation of highly ordered 2D AuNP arrays. Based on varying binding modes between AuNPs and TMV disks (Figure 27b), three different AuNP arrays were produced. The dual‐functional groups on the 2D TMV nanosheets enabled the simultaneous coassembly of AuNPs and QDs, resulting in binary functional NP arrays. To achieve conformational dynamics in AuNP arrays, Du et al. reported the creation of highly ordered and diverse 2D AuNP arrays on *p*42_1_2 ^C4/C98^RhuA lattices.^[^ ^19^ ^]^ They devised a rational design for RhuA building blocks to fabricate dynamic 2D functionalized protein arrays. These arrays were used to direct the precise assembly of AuNPs into highly ordered and diverse nanoarchitectures (**Figure** **28**a–a2). The 2D structures effectively regulated the conformation of AuNP arrays and served as excellent tools to validate the self‐assembly mode and the structural quality of the designed RhuA crystals. Subsequent redesign of the RhuA building blocks allowed for the predictable production of a novel protein lattice with controllably regulated conformational dynamics (Figure 28b–b4). Collectively, these studies underscore the potential of protein engineering to create highly organized and functionally relevant nanostructures. They not only expand the scope of protein‐based NP assembly but also suggest new pathways for developing adaptive nanomaterials.

### 4.2 QDs

**Figure 29:**
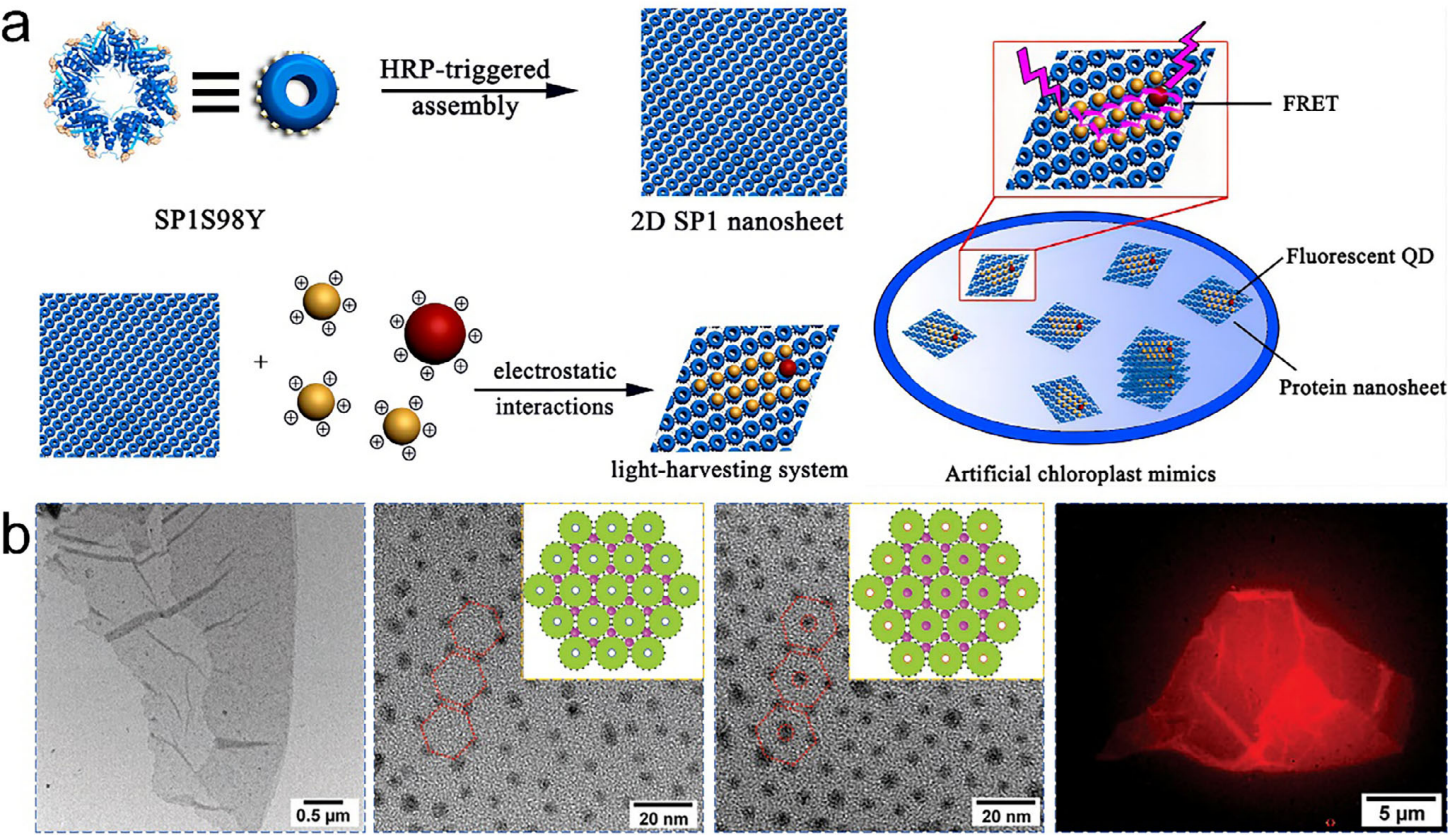
a) 2D CdTe QD arrays templated by 2D SP1 protein arrays, where energy can be transferred via direct FRET in QDs with different sizes, enhancing light harvesting properties. Reproduced with permission.^[^ ^69^ ^]^ Copyright 2017, American Chemical Society. b) Dual‐functional T103C‐TMV‐4His sheets direct the assembly of Ag_2_S QDs into the honeycomb and hexagonal arrays. Reproduced with permission.^[^ ^92^ ^]^ Copyright 2019, Wiley‐VCH Verlag GmbH and Co. KGaA, Weinheim.

2D protein arrays, celebrated for their exceptional self‐assembly capabilities and precise geometric control, have proved to be effective templates for constructing highly ordered QD arrays.^[^ ^189^, ^190^ ^]^ Trent et al. groundbreaking research utilized crystalline 2D protein assemblies to generate ordered CdSe‐ZnS QDs arrays.^[^ ^189^ ^]^ Building on this principle, Miao et al. employed the SP1 dodecamer as an appealing scaffold for QD organization.^[^ ^191^ ^]^ Electrostatic interactions between SP1 protein and CdTe QDs created sandwich nanowires. The integration of QDs of varying sizes within these protein nanowires enabled efficient Förster resonance energy transfer (FRET), suggesting that SP1 protein could be instrumental in assembling QD structures. However, the unidirectional transfer of energy along these nanowires posed a limitation. Zhao et al. presented a design strategy that combines covalent and noncovalent interactions to develop a protein‐based light‐harvesting system that emulates the functionality of chloroplasts.^[^ ^69^ ^]^ Mutant S98YSP1 proteins with Tyr residues can form well‐defined 2D arrays by enzyme‐triggered reactions. To further enhance their functionality, they integrated QDs with optical and electronic properties into the 2D nanosheets (**Figure** **29**a). QDs of different sizes were attached to the surface of 2D SP1 nanosheets via electrostatic interactions. QDs with different sizes serve as donor and acceptor chromophores, facilitating efficient FRET and mimicking the role of chlorophylls in natural light‐harvesting systems. Recent contributions from our group have further propelled the technology forward. Zhang et al. demonstrated the precise self‐assembly of Ag_2_S QDs into ordered 2D templates derived from TMV disks.^[^ ^92^ ^]^ Using 2D T103C‐TMVCP‐4His monolayer sheets as templates, they achieved two types of QDs arrays arranged in honeycomb or hexagonal patterns (Figure 29b). The presence of two distinct functional groups on the TMV nanosheets enabled the simultaneous coassembly of AuNPs and CdSe@ZnS QDs, resulting in binary functional NP arrays. In parallel, Du et al. reported the creation of highly ordered and diverse 2D Ag_2_S QD arrays on *p*42_1_2 ^C4/C98^RhuA lattices.^[^ ^19^ ^]^ These lattices were pivotal in directing the precise assembly of NPs into highly ordered and diverse nanoarchitectures. These advancements not only broaden the scope of protein‐based NP assembly but also pave the way for the development of adaptive nanomaterials that can respond to environmental stimuli.

### 4.3 Other Inorganic NPs

**Figure 30:**
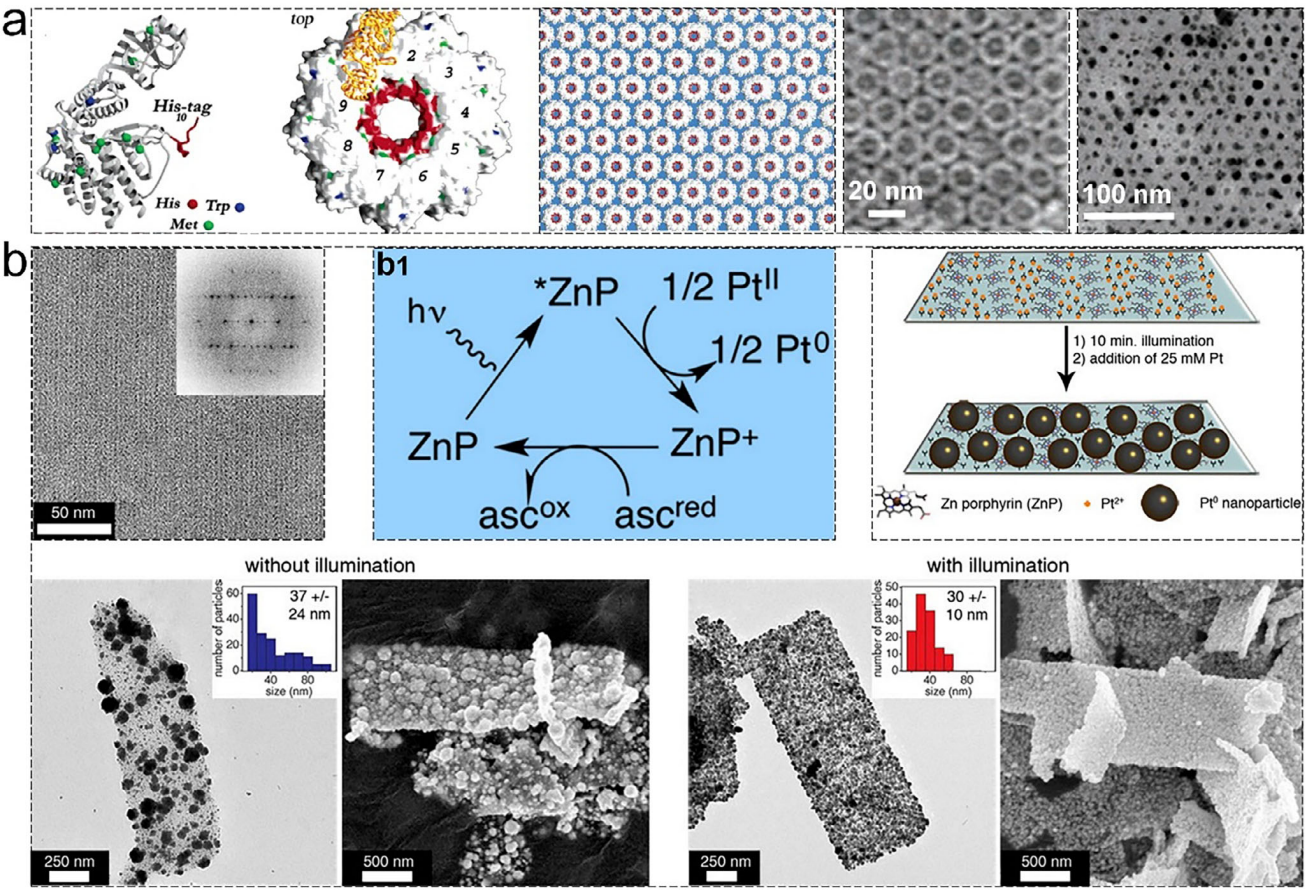
a) Hexagonal packing of chaperonins into 2D crystals for the assembly of Ni‐Pd or Co‐Pd ions into 2D NP arrays. Reproduced with permission.^[^ ^192^ ^]^ Copyright 2005, American Chemical Society. b) Redox‐controlled growth of Pt NPs on 2D RIDC3 surfaces, facilitated by integrated heme centers. Reproduced with permission.^[^ ^20^ ^]^ Copyright 2014, National Academy of Sciences.

2D protein arrays, characterized by a rich array of functional groups on their surfaces, ensure the uniform distribution and stable immobilization of inorganic ions within the arrays. This enhances both the functionality and the application potential of the resulting material. McMillan et al. explored the use of 2D‐designed chaperonin arrays for templating inorganic ions.^[^ ^192^ ^]^ They engineered a heat shock protein TF55β variant (by deleting the apical loop amino acid residues and attaching the His10 peptide), which self‐assembled into chaperonin with a core rich in 180 imidazole groups. These chaperonin proteins were arranged in hexagonal 2D arrays with the periodic His‐rich cores anchoring Ni‐Pd or Co‐Pd ions for the on‐site synthesis of NPs, producing 2D NP arrays (**Figure** **30**a). Building on this concept, Brodin et al. delved into the assembly of highly stable, redox‐active 2D protein assemblies with unique emergent properties.^[^ ^20^ ^]^ The 2D Zn‐RIDC3 arrays can function as chemically active scaffolds to facilitate NP assembly, such as the growth of Pt⁰ nanocrystals (Figure 30b). The Zn‐RIDC3 building block, equipped with a Zn‐porphyrin (ZnP) cofactor, can act as a potent reductant/oxidant under visible light. Consequently, Pt^2+^ ions were reduced to form Pt NPs on 2D Zn‐RIDC3 arrays under light excitation, resulting in 2D PtNP arrays. This study emphasizes the dual role of 2D protein assemblies: they act not only as structural templates but also as active controllers of redox‐driven NP growth, leveraging the specific functional activity of the protein building blocks.

### 4.4 Biomolecules

**Figure 31:**
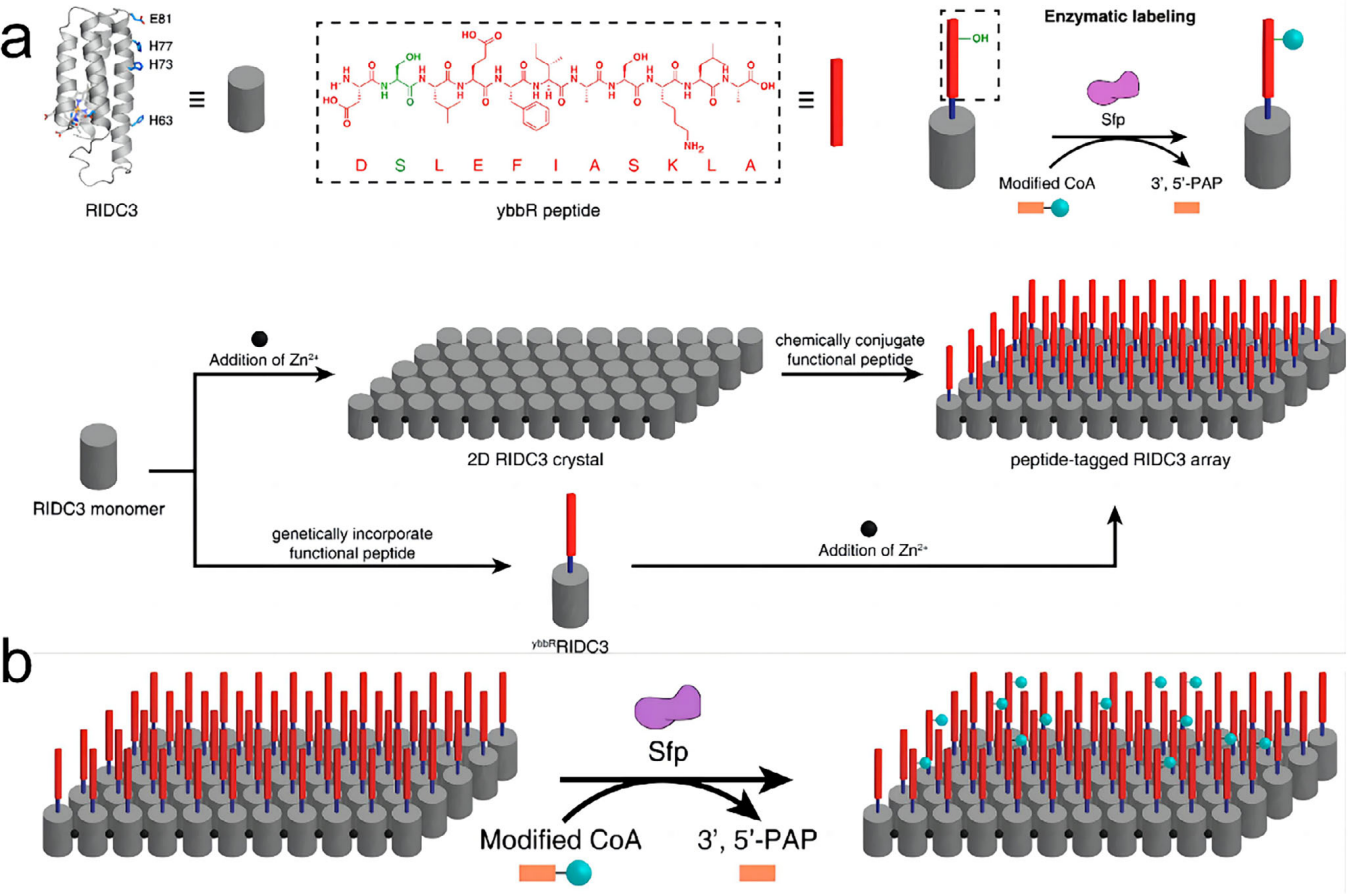
Design and assembly of peptide‐tagged 2D RIDC3 arrays (a) and the subsequent enzymatic labeling (b). Reproduced with permission.^[^ ^21^ ^]^ Copyright 2020, American Chemical Society.

2D protein arrays are particularly attractive scaffolds due to their high‐density display of functional peptides. Subramanian et al. used 2D RIDC3 arrays and ^C98^RhuA arrays as 2D templates, which served as recognition elements to enable enzyme‐mediated modification.^[^ ^21^ ^]^ Functional peptide ybbR was grafted onto a 2D RIDC3 array surface by chemical conjugation or genetic incorporation (**Figure** **31**a). By site‐specific modification of ybbR with modified CoA substrates, CoA‐labeled 2D RIDC3‐ybbR arrays were obtained under the catalysis of PPTase (phosphopantetheinyl transferases) Sfp (Figure 31a,b). Then, GFP‐modified ybbR was installed on the 2D RIDC3‐CoA array surface via Sfp‐mediated conjugation, forming 2D GFP‐modified RIDC3 arrays. Similar results were achieved with 2D ^C98^RhuA arrays. The study showed that enzyme‐directed surface modification could site‐specifically alter the surface of 2D protein arrays. Importantly, the site‐specific modifications achieved by both genetic and chemical strategies that did not disrupt the 2D array structures. This study underscores the potential of chemoenzymatic strategies to construct multicomponent protein systems with hierarchical organization, opening up new avenues for the development of sophisticated biomaterials.

## 5 Function and Applications

2D protein arrays have emerged as versatile platforms due to their unique structural and functional properties, offering significant advantages in materials and health science. These arrays leverage the inherent characteristics of protein molecules to create innovative biomaterials.^[^ ^17^ ^]^ Thus, this section will highlight some recent advancements in the functional applications of 2D protein arrays, focusing on biomedicine (Stimulate T cell proliferation and immune responses, and receptor clustering), photosystems, catalysis, and membrane filtration.

### 5.1 Stimulate T Cell Proliferation and Immune Responses

**Figure 32:**
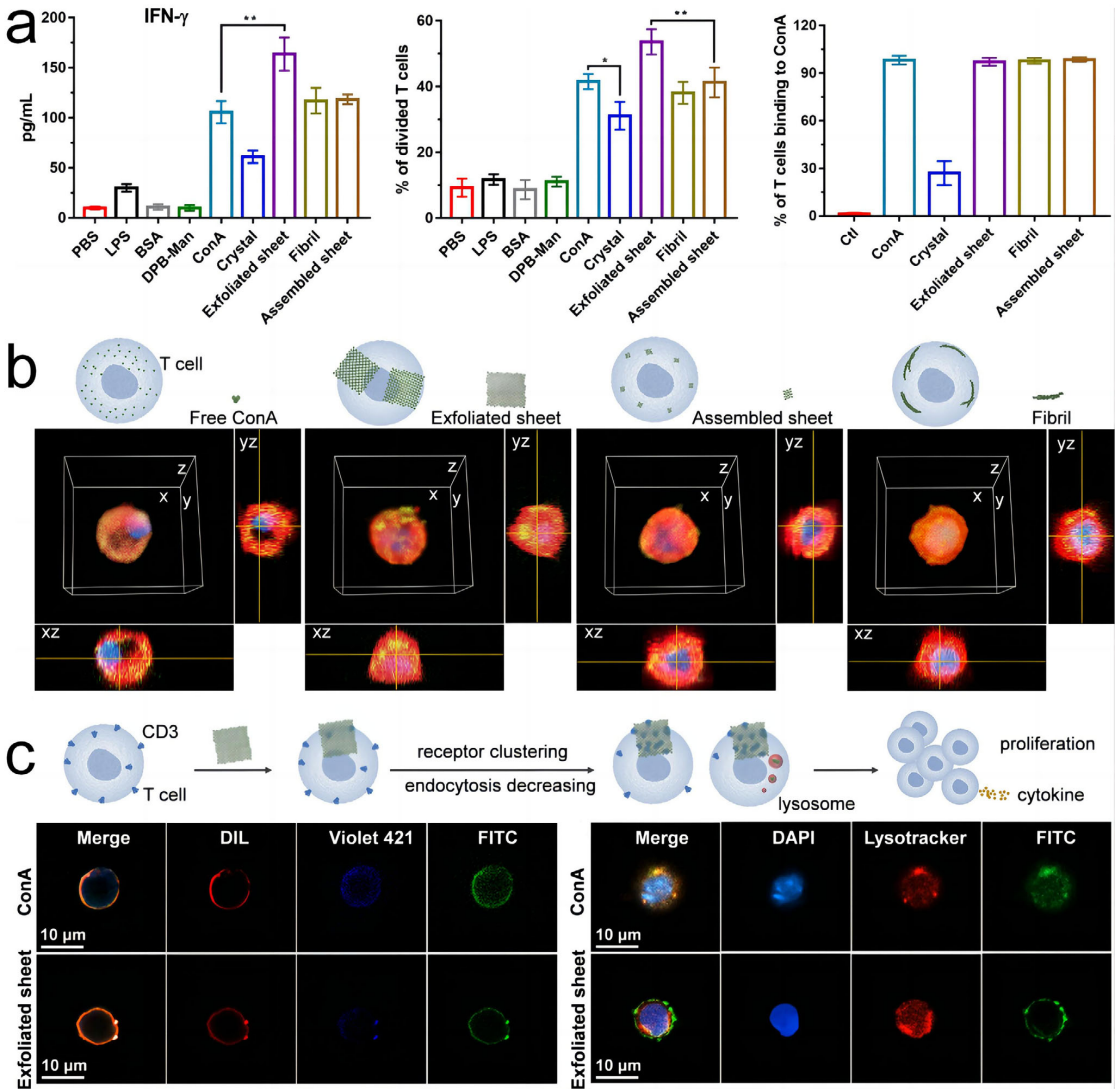
a) IFN‐γ secretion levels measured by ELISA after T cells were incubated with ConA in various morphologies for 72 h. b) 3D imaging of the membrane distribution of ConA in different morphologies. c) Confocal images of CD3 molecules on the cell membrane. Reproduced with permission.^[^ ^110^ ^]^ Copyright 2024, American Chemical Society.

Protein architectures with varying dimensions and sizes have been found to significantly modulate their biological functions.^[^ ^193^ ^]^ For example, the intrinsic bioactive function of ConA containing signaling and immune cell function, the molecular mechanisms inducing autophagy and apoptosis in various cancer cell types, and activate T cell proliferation were explored.^[^ ^194^, ^195^, ^196^ ^]^ Based on this foundation, Li and colleagues focused on the immunostimulatory effects of different ConA assemblies, including 3D parent crystals, 2D exfoliated nanosheets, 1D exfoliated fibrils, and assembled nanosheets on T cell activation and proliferation.^[^ ^110^ ^]^ After T cells were incubated with these ConA architectures for 72 h, 2D exfoliated nanosheets exhibited the most potent immunostimulatory effects (**Figure** **32**a). Specifically, 53.2% of T cells underwent proliferation when being exposed to 2D nanosheets, compared to 41.5% for free ConA, 38.1% for 1D fibrils, and 40.9% for assembled nanosheets. The 3D parent crystals showed the least effectiveness, with only 31.1% of T cells proliferating. The larger size of the 2D exfoliated nanosheets might enhance the clustering of CD3 receptors on the T cell surface (Figure 32c,d), a critical step for T cell activation. Immunofluorescence staining of CD3 molecules further confirmed that larger materials effectively promoted receptor clustering. Endocytosis tracking experiments revealed that 2D nanosheets were more resistant to cellular internalization, allowing them to remain on the cell surface for extended periods, thereby sustaining their immunostimulatory effects. The 2D exfoliated nanosheets exhibited the most robust immunostimulatory effects, significantly increasing cytokines production and stimulating splenocyte proliferation. In contrast, free ConA, 1D fibrils, and assembled nanosheets did not show substantial differences in their ability to stimulate cytokine production and cell proliferation. This work underscores the importance of protein architecture in modulating immune responses and provides a foundation for the development of novel immunostimulatory materials.

### 5.2 Receptor Clustering

**Figure 33:**
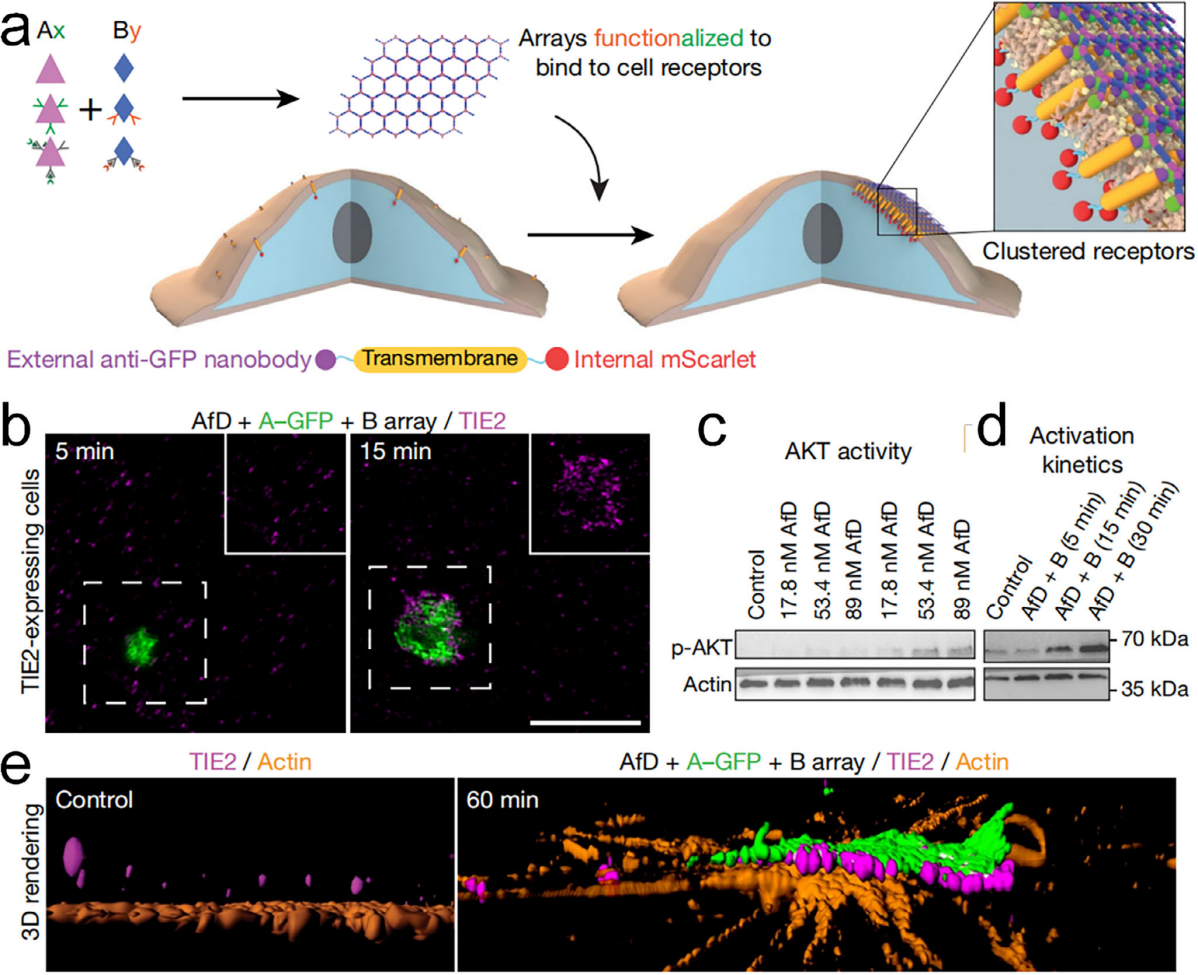
a) Schematic illustration of 2D array functionalization through genetic or post‐translational fusions. b–d) Clustering of TIE2 receptors induced by preformed arrays. e) 3D reconstruction of TIE2 receptor clustering, in the absence of arrays (Control) and 60 min after array introduction. Reproduced with permission.^[^ ^139^ ^]^ Copyright 2021, Nature Publishing Group.

Receptor clustering of cell membranes mediated by designed 2D protein arrays has been explored to mimic the natural organization of receptors, enabling the systematic study of receptor clustering effects on signaling pathways and cellular responses. Ben‐Sasson et al. reported the development of computationally designed, binary 2D protein arrays that interacted with 3T3 cell membranes and controlled TIE2 receptor clustering.^[^ ^139^ ^]^ *D*_2_ and *D*_3_ symmetric building blocks, chosen by systematical screening, self‐assembled with high fidelity into 2D arrays both in vitro and in vivo. Importantly, the 2D arrays allow for genetic and post‐translational modifications, enabling the fusion of other proteins and ligands (such as GFP) to cell receptors (**Figure** **33**a). These arrays can significantly induce extensive clustering of membrane TIE2, drive downstream protein recruitment, and activate signaling pathways (Figure 33b–e). Additionally, the assembled arrays on cell surface were observed to exhibit hexagonal architectures that were consistent with those obtained under in vitro conditions. Subsequently, they modified EGFR (epidermal growth factor receptors) onto the 2D array surface, which suppressed endocytosis due to their large sizes.

### 5.3 Photosystem

**Figure 34:**
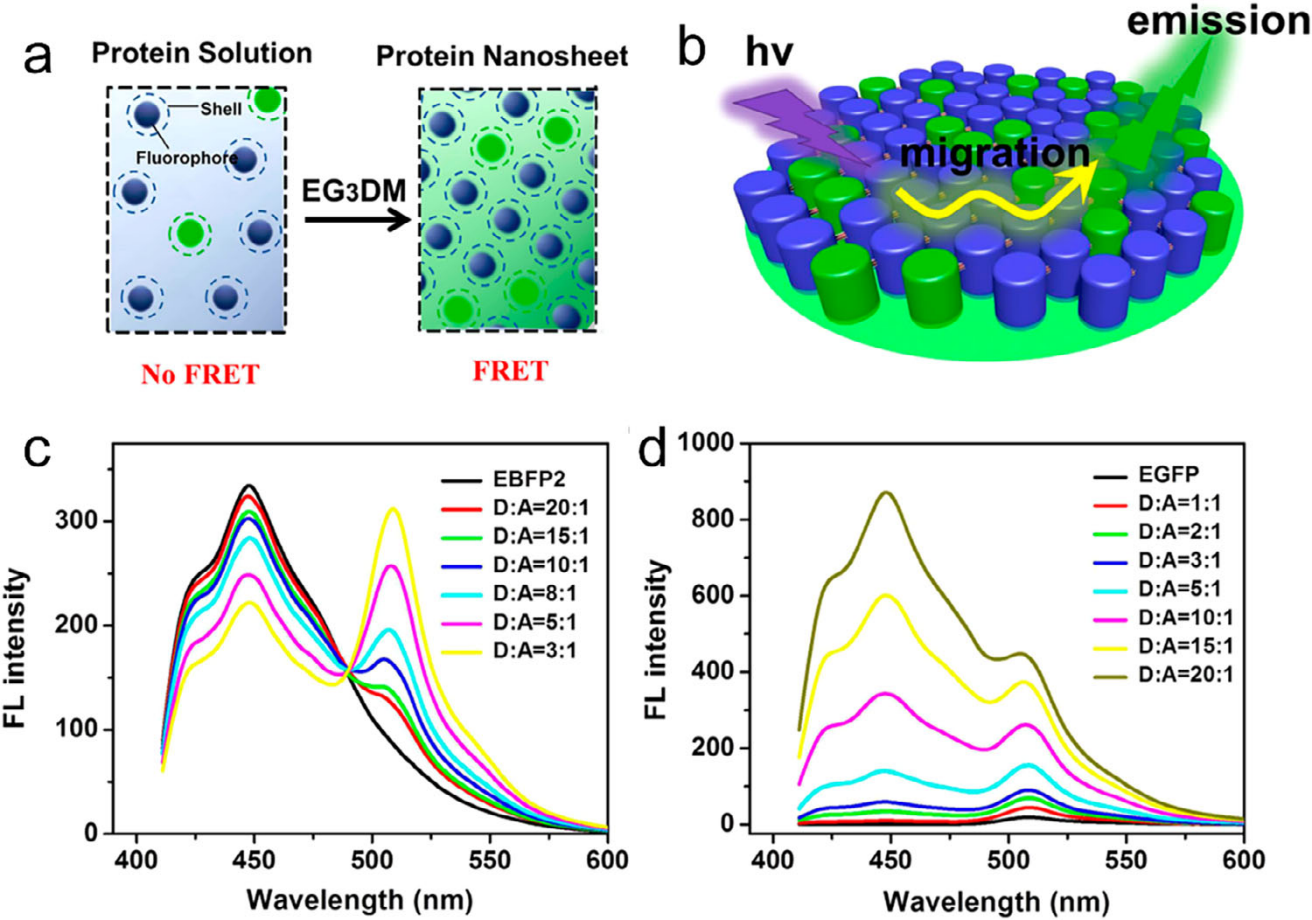
a) Schematic representation of dispersed fluorescent proteins self‐assembled into a densely packed protein nanosheet exhibiting high brightness and prominent FRET. b) Schematic of a light‐harvesting protein nanosheet, where absorbed energy can be transferred to the acceptor through direct FRET or sequential donor‐to‐donor transfers. c) FRET spectra of the coassembly system with varying concentrations of acceptor EGFP‐4C, at a fixed concentration of EBFP2‐4C (2 µm). d) FRET spectra of the coassembly system with varying concentrations of donor EBFP2‐4C, at a fixed concentration of EGFP‐4C (0.22 µm). Reproduced with permission.^[^ ^80^ ^]^ Copyright 2019, American Chemical Society.

Constructing periodic 2D arrays with fluorescent proteins can overcome the limitations of synthetic materials and achieve unexpected results. Inspired by the light‐harvesting systems in plants, Li et al. developed a template‐free method for creating highly ordered monolayered fluorescent protein nanosheets.^[^ ^80^ ^]^ They rationally chose 2D protein materials as foundational scaffolds. Yu et al. devised 2D EGFP‐4C (E4P) arrays to create single‐layer semiconductor‐decorated 2D materials and produced two mutants of the EBFP protein: EBFP2 (donor) and EGFP (acceptor). The two mutants coassembled into 2D protein arrays via covalent bonds. These chromophores in the arrays were well‐distributed and fixed in orientation (**Figure** **34**a,b). Due to their excellent anti‐self‐quenching properties, the 2D arrays exhibited efficient FRET even at high local concentrations (Figure 34c,d). By adjusting the ligands length, the 2D array size could be tuned, leading to the creation of micron‐sized light‐harvesting systems with higher FRET.

Besides efficient FRET, 2D protein arrays can also integrate QDs and photosensitive pigments, leading to the development of powerful artificial chloroplast‐like molecular machines. Zhao et al. utilized the electrostatic interactions between 2D SP1 protein arrays and CdTe QDs to construct 2D QD arrays.^[^ ^69^ ^]^ 2D protein arrays incorporated QDs of different sizes, which acted as donor and acceptor chromophores on the 2D protein array surface, produced a pronounced FRET effect. This mimics the light‐harvesting behavior of thylakoid membranes in natural chloroplasts.

### 5.4 Catalysis

**Figure 35:**
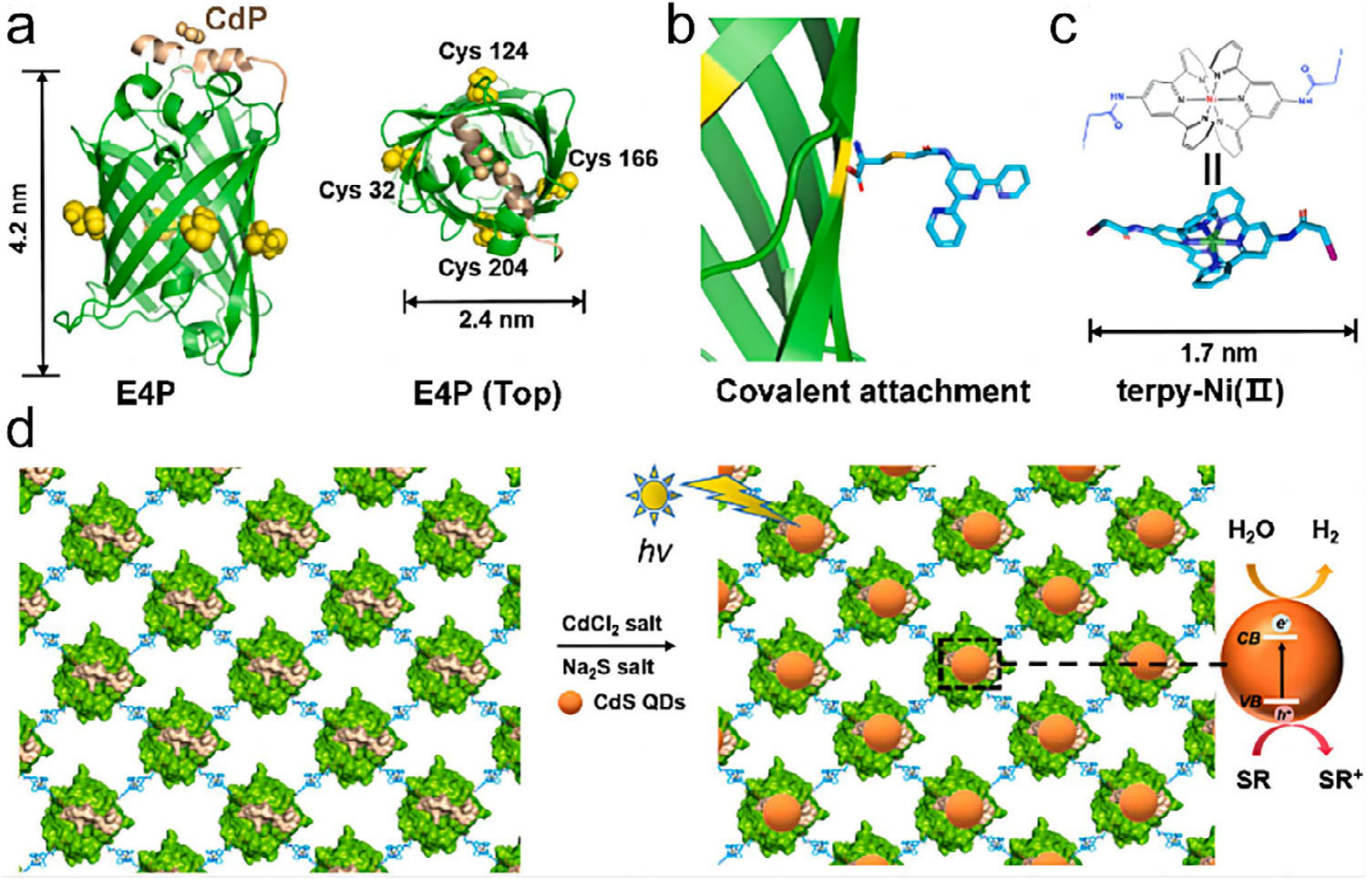
a) Side and top views of E4P, highlighting the Cd‐binding peptide and Cys residues. b) Covalent attachment of terpyridine molecules to the sulfhydryl groups of Cys residues on the E4P surface. c) Formation of dimers through Ni(II) coordination with terpyridine molecules. d) Schematic illustration of the photocatalytic process in E4P‐terpy‐Ni@CdS QD biohybrids. Reproduced with permission.^[^ ^197^ ^]^ Copyright 2024, Elsevier B.V.

Stable photocatalytic hydrogen production systems have been advanced by utilizing 2D protein materials as scaffolds. Yu et al. devised 2D EGFP‐4C (E4P) arrays to create single‐layer semiconductor‐decorated 2D materials under mild conditions, resulting in enhanced light‐driven hydrogen generation.^[^ ^197^ ^]^ They engineered the green fluorescent protein EGFP to produce EGFP‐4C (E4P) mutants incorporating Cd‐binding peptide (**Figure** **35**a). Terpyridine molecules were grafted onto the sulfhydryl groups of the mutants (Figure 35b). The mutants are subsequently self‐assembled into 2D protein arrays via terpyridine‐Ni (II) coordination (Figure 35c). The resultant 2D protein arrays displayed high‐density Cd‐binding peptides, enabling precise control over the assembly of CdS QDs on the protein surface. 2D 4.3 nm CdS QDs nanosheets were successfully produced, showing significant photocatalytic efficiency (Figure 35d). These achievements underscore the potential for stable and highly efficient hydrogen production, emphasizing the adaptability and biocompatibility of protein scaffolds in photocatalysis.

### 5.5 Membrane Filtration

**Figure 36:**
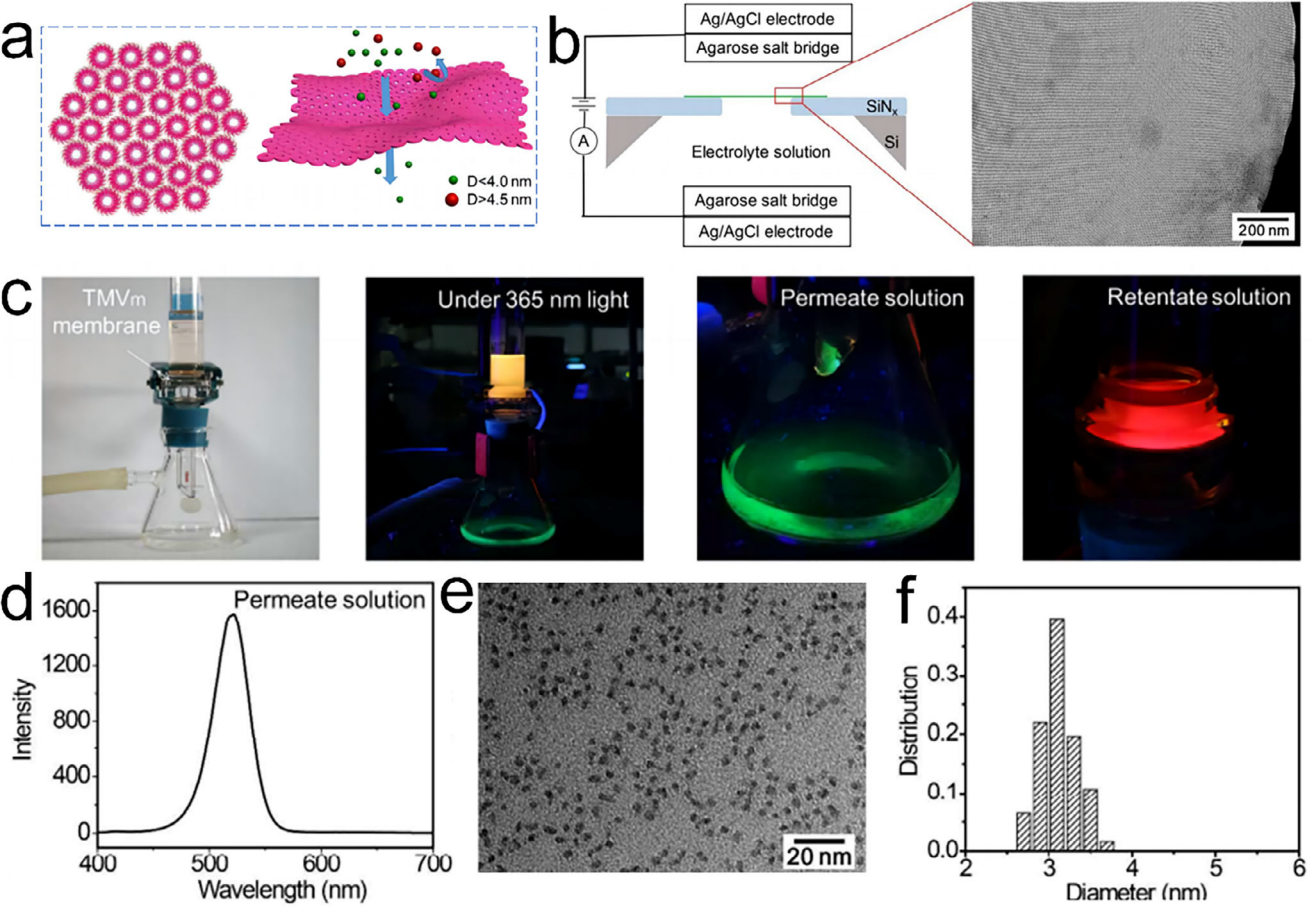
a) Schematic representation of the formation and separation of 2D TMV nanosheets. b) Experimental setup for the separation process. c) The separation installation and the separation of a mixed solution of CdSe@ZnS QDs. d) Fluorescence spectra and e) TEM images of the permeate solution. f) Corresponding size distribution analysis. Reproduced with permission.^[^ ^78^ ^]^ Copyright 2018, American Chemical Society.

In filtration and separation processes, 2D protein arrays have demonstrated remarkable potential, driven by their capability to offer selective permeability and a large surface area conducive to molecular interactions. Our group designed a free‐standing, porous 2D single‐layer ultra‐large TMV nanosheet with ordered 4 nm pores.^[^ ^198^, ^199^ ^]^ which were then used to prepare ultrathin ultrafiltration membranes (**Figure** **36**a,b).^[^ ^78^ ^]^ These membranes (thickness ≈ 40 nm) exhibited precise separation capability for QDs of varying sizes (Figure 36c–f). The ordered nanoporous structure and ultra‐thin 2D TMV arrays promise rapid and accurate selective separation of biomacromolecules and NPs with different sizes.

## 6 Conclusion and Outlook

The advent of 2D protein arrays as a focal point in research herald significant advancements in both fundamental science and practical applications, particularly in the realms of biomaterials and nanotechnology. This review intends to summarize the supramolecular methodologies used to construct 2D protein structures. Key strategies have meticulously been analyzed to elucidate how they influence the order and orientation of 2D protein self‐assembly. These approaches demonstrate the power of rational design and precise site‐specific modifications in guiding protein interactions, which is essential for the development of large‐scale, highly ordered 2D architectures. The ability to engineer these sophisticated assemblies extends beyond mere structural innovation; it opens new avenues for creating biomaterials that mimic natural systems with programmable properties.

Proteins offer unparalleled versatility in self‐assembly and coassembly processes, providing a rich toolkit for the creation of intricate 2D biomaterials that closely resemble those found in nature. Unlike traditional chemical molecules, the diverse and complex nature of protein monomers and oligomers enables the design of multifunctional surfaces with atomic‐level precision. This precision is critical for applications of 2D protein arrays such as catalysis and biomedicine, where finely tuned interactions with biological entities are essential. Moreover, proteins exhibit unique properties at the atomic scale, allowing for selective modification with functional groups, molecules, and NPs to achieve specific attributes of 2D protein arrays for a wide range of applications. Therefore, the field of 2D protein assembly has become a vibrant area of multidisciplinary research, offering unique advantages for both understanding natural assembly processes and addressing challenges in materials and health sciences.

Despite notable progress in the assembly strategies and applications of 2D protein arrays, several avenues remain to be explored in future research. First, most existing 2D protein arrays are based on naturally occurring proteins, limiting their structural diversity and functional versatility to the inherent properties of these natural proteins. However, with advancements in artificial intelligence tools such as AlphaFold, it is now possible to design 2D protein arrays de novo, offering an unprecedented opportunity to create non‐natural 2D protein arrays with more complex structures and functions. This shift from mimicking nature to surpassing it could significantly expand the scope of 2D protein research. Second, while current 2D protein arrays are primarily used to assemble single functional units, the assembly of multiple functional units in an ordered manner remains a challenge. Future research should focus on the development of controlled assembly techniques that enable the precise, spatially ordered arrangement of diverse functional units within 2D protein arrays. Achieving this will allow for the fine‐tuning of physical, chemical, and biological interactions between units, potentially enhancing the functional capabilities of 2D protein arrays and broadening their applications in areas such as biocatalysis and biomedicine. Finally, the majority of 2D protein technologies remain confined to laboratory‐scale research, with limitations in terms of yield, uniformity, and scalability. No large‐scale production methods have been reported to date. To facilitate the practical application of 2D protein arrays, it is essential to develop strategies for the controlled assembly of 2D protein arrays that are structurally uniform, stable, and of high quality. Innovations in mass production techniques will be crucial for translating the potential of 2D protein arrays into real‐world applications.

In conclusion, the pursuit of highly ordered 2D protein arrays with multifunctional capabilities represents both a significant challenge and a unique opportunity. The integration of diverse scientific disciplines will not only deepen our understanding of natural assembly processes but also drive the creation of advanced materials with broad applications. Continuous progress in this domain promises to unlock new frontiers in materials science and medicine, positioning 2D protein arrays as a cornerstone of future technological and healthcare advancements.

## Conflict of Interest

The authors declare no conflict of interest.
